# Targeting RAD51-BRCA2 Interaction to Enhance Synthetic Lethality with Olaparib in Pancreatic Cancer: Development of a Novel Phenyl Furan-Quinoline-Carboxylic Acid Series

**DOI:** 10.1021/acsmedchemlett.5c00711

**Published:** 2026-01-26

**Authors:** Giovanni Ferrandi, Greta Bagnolini, Laura Poppi, Mirco Masi, Viola Previtali, Angela Andonaia, Giulia Varignani, Marina Veronesi, Francesca De Franco, Federico Falchi, Giuseppina Di Stefano, Stefania Girotto, Marinella Roberti, Andrea Cavalli

**Affiliations:** ∇ Department of Pharmacy and Biotechnology, 9296 University of Bologna, 40126 Bologna, Italy; ‡ Computational and Chemical Biology, 121451 Istituto Italiano di Tecnologia, 16163 Genoa, Italy; § TES Pharma S.r.l., I-06073 Corciano, Perugia, Italy; ∥ Department of Medical and Surgical Sciences, 9296 University of Bologna,40126 Bologna, Italy; ⊥ Structural Biophysics Facility, 121451 Istituto Italiano di Tecnologia, 16163 Genoa, Italy; # Centre Européen de Calcul Atomique et Moléculaire (CECAM), Ecole Polytechnique Fédérale de Lausanne (EPFL), 1015 Lausanne, Switzerland

**Keywords:** Synthetic lethality, RAD51-BRCA2, protein−protein interaction inhibitors, small molecules, pancreatic cancer

## Abstract

Synthetic lethality has proven to be a tactical paradigm to design synergistic anticancer drug combinations. In this context, we leveraged BRCA2 and PARP as a synthetic lethal target pair to consolidate the use of small molecule inhibitors of RAD51-BRCA2 protein–protein interaction as inducers of the BRCAness phenotype that sensitizes *BRCA2*-functional cancer cells to PARP inhibitors. Starting from compound **1**, a phenyl furan-carboxyquinoline, we developed a series of analogues, leading to derivative **19**. This compound effectively inhibits RAD51-BRCA2 interaction, impairs homologous recombination, and synergizes with olaparib in BxPC-3 pancreatic cancer cells, inducing synthetic lethality in both 2D and 3D spheroids. Additionally, **19** showed efficacy in human pancreatic cancer cells and no toxicity in normal pancreatic cells, positioning it as an early tool compound and a starting point for further optimization.

## Graphical abstract

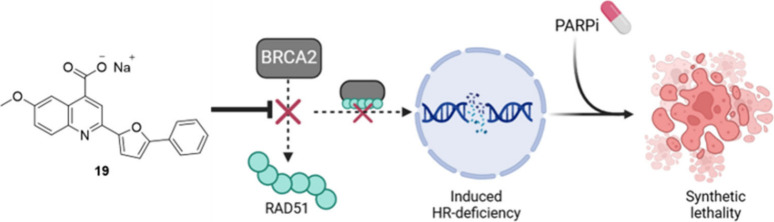

Synthetic lethality (SL) was initially proposed to target tumors with specific genetic mutations and selectively induce cell death in tumor cells by targeting synthetic lethal partners.^1,2^ This approach marked a significant advance in precision medicine, offering the potential to target cancer cells while sparing healthy ones. However, the “one-mutation-one-drug” paradigm faces considerable limitations, including acquired resistance, commonly experienced in targeted monotherapies, and a narrow population of eligible patients due to the reliance on specific mutations.

In recent years, the concept of SL has been expanded beyond single gene pairs to synthetic lethal interactions (SLIs),^3^ encompassing functionally connected pathways that can be exploited through synergistic drug combinations. In principle, this paradigm shift enables the pharmacological recreation of SL effects without requiring pre-existing genetic alterations, limiting resistance and broadening patient eligibility. Since it was originally named by our group “fully-small-molecules-induced SL”,^4^ the number of such synergistic combinations has been significantly expanding, in particular by targeting DNA Damage Response (DDR) pathways, hyperactivated in many cancers.^5^ By exploiting the clinical success of PARP inhibitors (PARPi), medicinal chemistry has focused on small molecules targeting DDR proteins that induce homologous recombination deficiency (HRD), a phenotype that may be synthetic lethal with PARP inhibition in cancer cells.^6^

To this aim, in the last years, we have focused on targeting RAD51-BRCA2 protein–protein interaction (PPI) in pancreatic cancer.^4,7,8^ RAD51-BRCA2 PPI is crucial for recruiting RAD51 to repair DNA double-strand breaks *via* homologous recombination (HR). RAD51-BRCA2 PPI is structurally characterized by two pocket-like hot spots on the RAD51 surface, named Zone I (lodging BRC4’s FxxA motif) and Zone II (lodging BRC4’s LDFE motif).^9^ Inhibiting this interaction results in HRD, which is synthetically lethal with PARP inhibition. Through various medicinal chemistry strategies, including virtual screening (VS),^4,8,10^ dynamic combinatorial chemistry (DCC),^11^ and ^19^F-NMR fragment-based screening^7^ followed by chemical optimization, we developed different classes of RAD51-BRCA2 PPI inhibitors. So far, the best result was obtained starting from a VS campaign based on high-throughput docking targeting Zone II, using the cocrystal structure of RAD51-BRC4 (PDB ID: 1N0W; Section S1).^9^ This structure, which captures the human RAD51-BRCA2 interaction, is considered the most suitable template for developing PPI inhibitors compared to other available RAD51 structures.^12–17^

Among the 42 compounds selected, purchased, and tested *via* ELISA, we identified two potential hit compounds. The first top hit dihydropyrazoline **ARN22064** yielded **ARN24089** (named **35d**,^4^ Figure S1), a RAD51-BRCA2 PPI inhibitor that induces SL when combined with olaparib (ola), the only PARPi approved for pancreatic cancer so far.^18^ **ARN24089** has recently enabled the exploration of “within-pathway” SL, which exploits lethal interactions between targets within the same pathway.^19^ However, the poor solubility of **ARN24089** affected its progression to *in vivo* studies. Nonetheless, the VS approach proved to be effective in obtaining RAD51-BRCA2 PPI inhibitors.

Therefore, for this study, we explored the second-best candidate from the same VS campaign, **ARN22142** (**1**, Figure 1A, Table 1). **1** is a dicarboxylic acid with a phenyl furan-quinoline scaffold, previously synthesized by Horak et al.^20^ Upon resynthesis (Scheme 1), **1** inhibited RAD51-BRC4 interaction in ELISA with an EC_50_ of 12.7 ± 1.7 μM and an E_max_ exceeding 90% (Table 1), calculated by using **CAM833** as reference inhibitor.^21^ In BRCA2-proficient BxPC-3 pancreatic cancer cells, **1** only moderately inhibited HR, with a maximum 35% inhibition at 50 μM (Table 1, Figure S2A). Consistently, the cotreatment with ola yielded only an additive effect (Figure S2B), as indicated by the Interaction Index (I. Index) 0.94 at 50 μM (Table 1). Nonetheless, **1** showed high kinetic solubility in PBS (234 ± 9 μM), making it a suitable starting point for our medicinal chemistry campaign.

**Figure 1:**
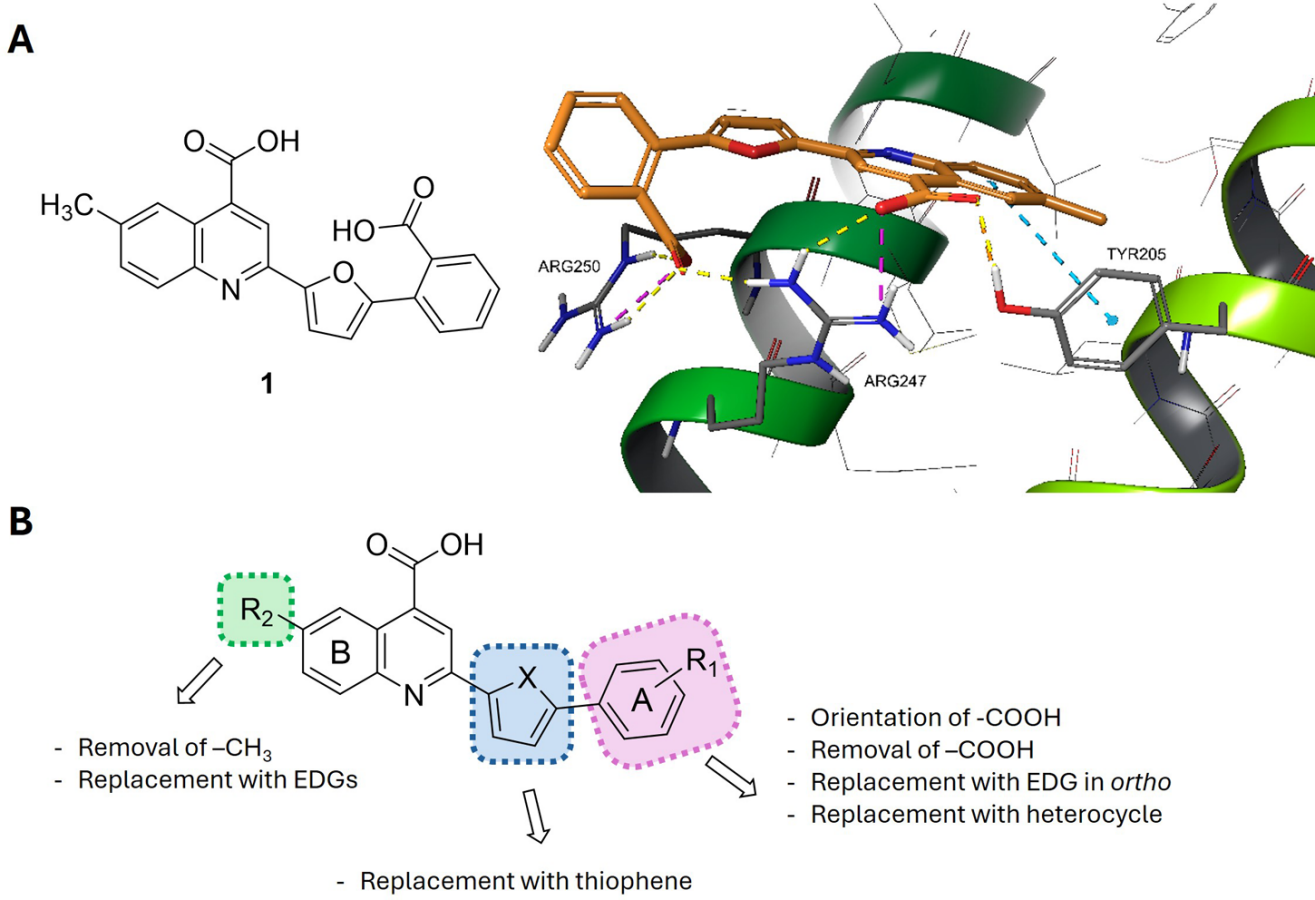
**A**) Structure of hit **1** and proposed docking of **1** into the LFDE binding site (Zone II) of RAD51 (PDB ID 1N0W); **B**) SAR overview of phenylfuran-carboxyquinoline derivatives.

**Scheme 1:**
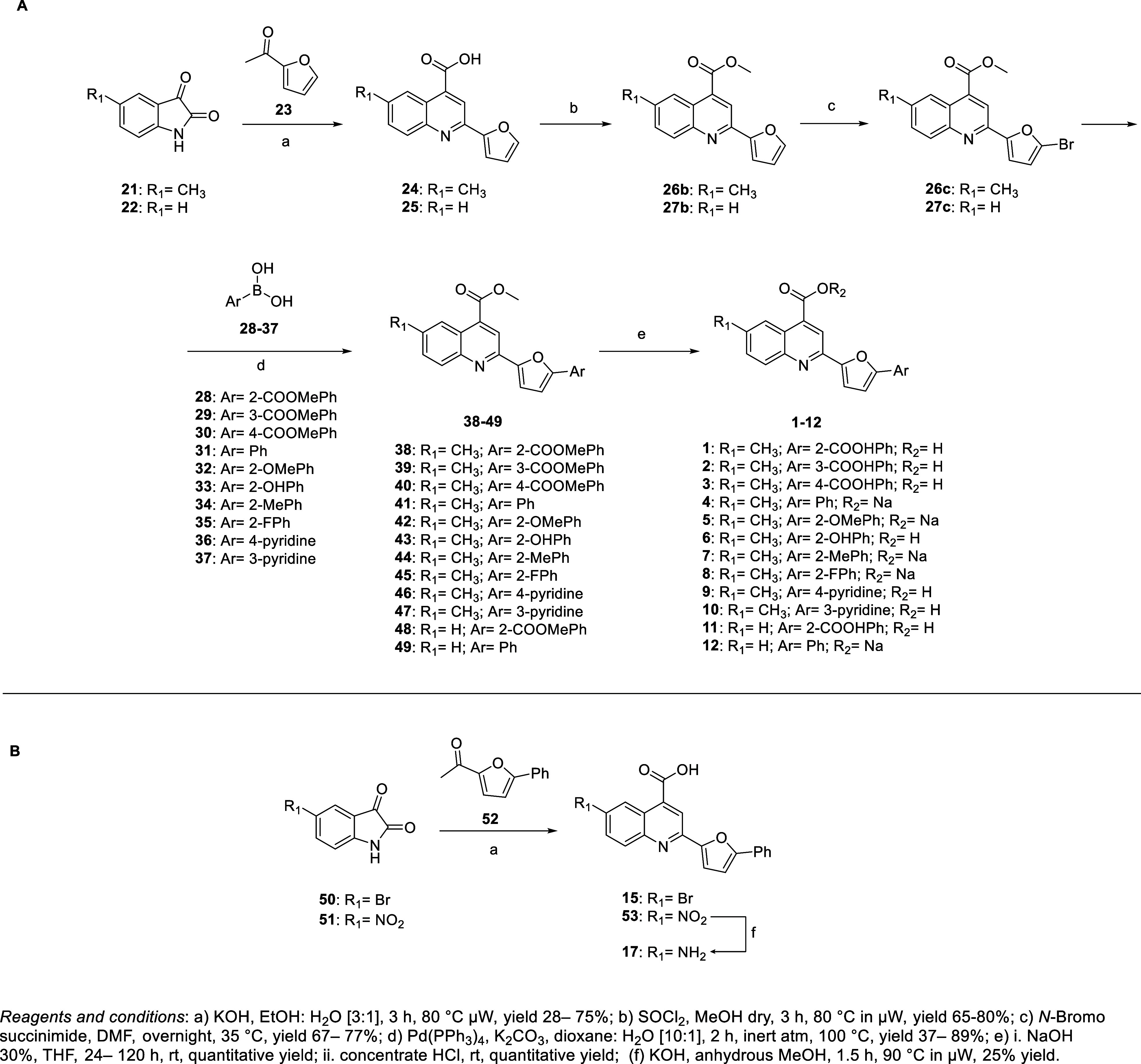
Synthetic Strategy *via* Pfitzinger Reaction for Compounds (A) **1**–**12** and (B) **15**, **17**

**Table 1:**
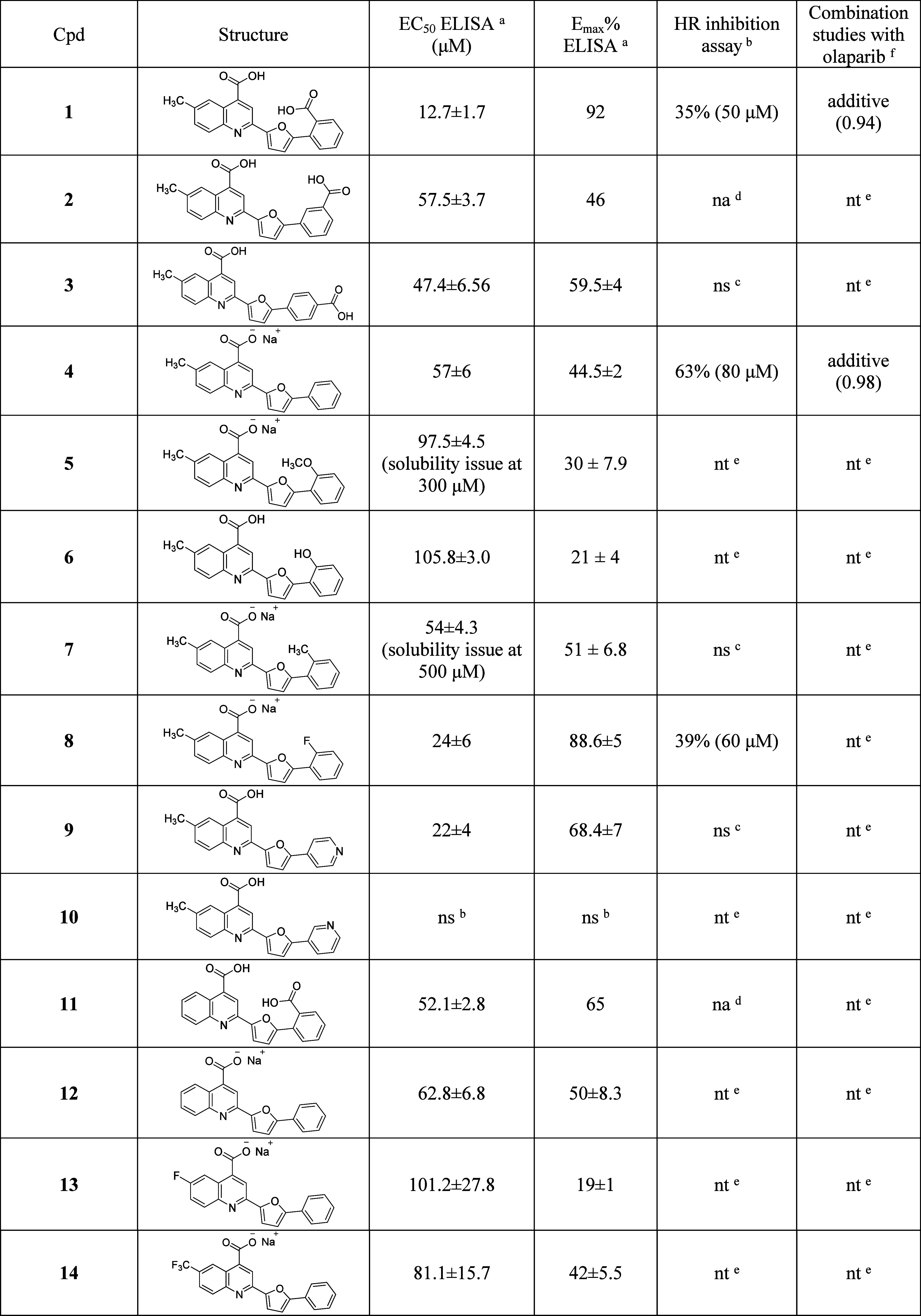

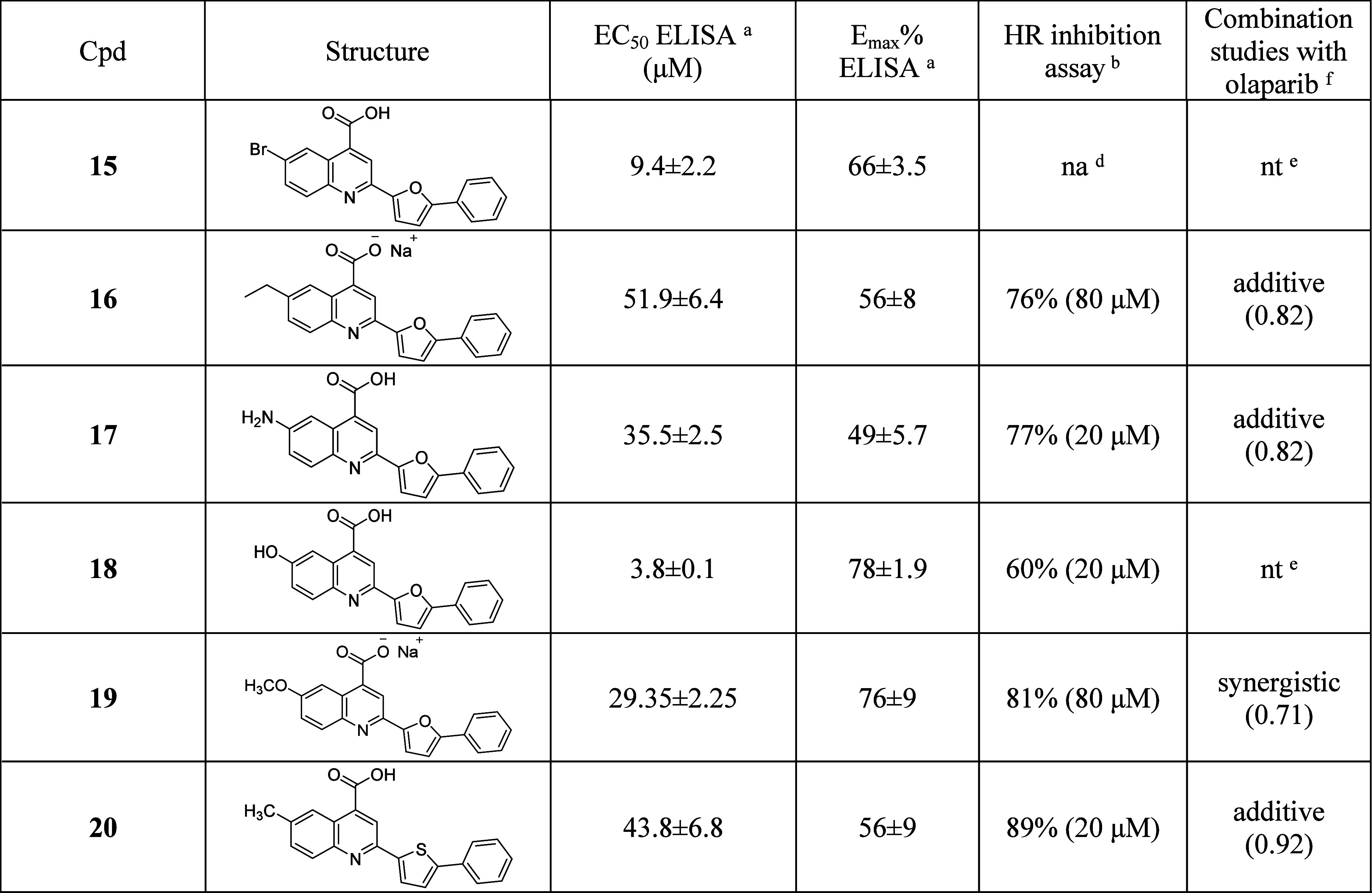
Structures and Preliminary Screening of Hit Compound 1 and Its Derivatives **2**–**20**

a ELISA assay results (Section S3).

b HR Quick Assay (HR-QA).

c Not soluble or solubility issues.

d Not active.

e Not tested (out of cutoff set at 60 μM or not soluble in ELISA).

f Combination effect with olaparib in BxPC-3 cells.

To guide the chemical exploration of **1**, we studied its binding mode on Zone II (Figure 1A). Our docking hypothesis suggests that the quinoline reaches the inner part of the pocket, where it establishes a net of H-bonds with Arg247 and Tyr205 and an ionic interaction with Arg247 by means of the carboxylic group, plus an additional π/π interaction with Tyr205. Additionally, the second carboxylic group appears to form an H-bond and a guanidinium-carboxylate interaction with Arg250, plus an additional H-bond with Arg247. Overall, the furan and the unsubstituted portion of the phenyl ring result in the most solvent-exposed area.

We first investigated the role of the ortho carboxylic group on the phenyl ring (pink, Figure 1B) to determine its essentiality for PPI inhibitory activity. First, we reoriented the group to *meta* and *para* positions that yielded compounds **2** and **3**, to assess whether it could affect the interactions with basic residues Arg247 and Arg250 (Table 1). Then we removed the group entirely, generating derivative **4**. Next, we explored substitutions at the *ortho* position with small electron-donating groups (EDGs): methoxy as a hydrogen-bond acceptor (**5**), hydroxyl as a donor (**6**), methyl (**7**), and fluorine (**8**) (Table 1). To improve solubility, we also replaced the phenyl ring with pyridine, placing the nitrogen at both the *meta* and *para* positions (**9**–**10**, Table 1).

In the second round, we focused on the quinoline moiety (green, Figure 1B), which contributes mainly through π–π stacking with Tyr205 (Figure 1A). The 6-methyl group appeared nonessential, suggesting it could be modified for further optimization. We first removed the methyl group (**11**), both alone and in combination with removal of the phenyl carboxylic group (**12**). Then we replaced 6-methyl with diverse substituents varying in size, polarity, and functional groups: fluorine, methoxy, trifluoromethyl, ethyl, bromine, hydroxyl, and amine (**13–19**), all combined with an unsubstituted phenyl ring (Table 1). Finally, we replaced the furan core with thiophene (**20**, Table 1; blue, Figure 1B) to evaluate the importance of the central scaffold.

To synthesize compound **1** and analogs modified in the phenyl ring region, **2**–**12**, we performed a Pfitzinger reaction with the appropriate commercially available isatins (**21**, **22**) and acetylfuran **23** to yield quinoline intermediates (**24**, **25**), which were converted to methyl esters (**26b**, **27b**) and brominated to yield intermediates (**26c**, **27c**). The subsequent Suzuki coupling with appropriate boronic acids (**28**–**37**) gave **38**–**49**, which were hydrolyzed with sodium hydroxide aqueous solution to yield the final **1**–**12**. Final compounds **4**–**5**, **7**–**8**, and **12** were obtained as sodium salts, while others were acidified to yield undissociated acids (Scheme 1A). The Pfitzinger reaction was also used to synthesize **15** and **17** (Scheme 1B). To improve selectivity and yield, we optimized the synthesis by preparing the phenylfuranyl-ketone **52** (reported in Scheme S1) for reaction with commercially available isatins (**50**, **51**). Compound **15** was synthesized in one step from the Pfitzinger reaction of 5-bromoisatin **50** and ketone **52**, while **17** was synthesized from 5-nitroisatin **51** and ketone **52**, followed by reduction of the nitro intermediate **53** to the amine derivative **17** (Scheme 1B).

To obtain additional analogues modified at position 6 of the quinoline, we employed the Doebner reaction, following the optimized protocol by Komatsu et al.^22^ This approach was chosen to address the limited availability of substituted isatins. Using this strategy, we synthesized derivatives **13**–**14**, **16**, and **18**–**20** (Scheme 2). For **13**–**14**, **16**, and **19**, substituted anilines (**54**–**57**), furfural **58**, boron-diethyl ether trifluoride **59** and pyruvic acid **60** underwent a Mannich-type reaction to form intermediates (**61**–**64**) (Scheme 2A). In turn, **61**–**64** were esterified to form **65b**–**68b**, which were then brominated to **65c**–**68c**, suitable for Suzuki coupling with phenyl boronic acid **31** to yield **69**–**72**. Final compounds **13**–**14**, **16**, and **19** were obtained as sodium salts by basic hydrolysis of the corresponding methyl esters (Scheme 2A). Analogue **18** was synthesized *via* the Doebner reaction between commercially available aniline **73** and intermediate **74** (reported in Scheme S2) (Scheme 2B). Finally, the thiophene derivative **20** was synthesized through a Doebner reaction of *para*-toluidine **75** with aldehyde **76** to yield **77** (Scheme 2C), which underwent Suzuki coupling to give **20** (Scheme 2C).

**Scheme 2:**
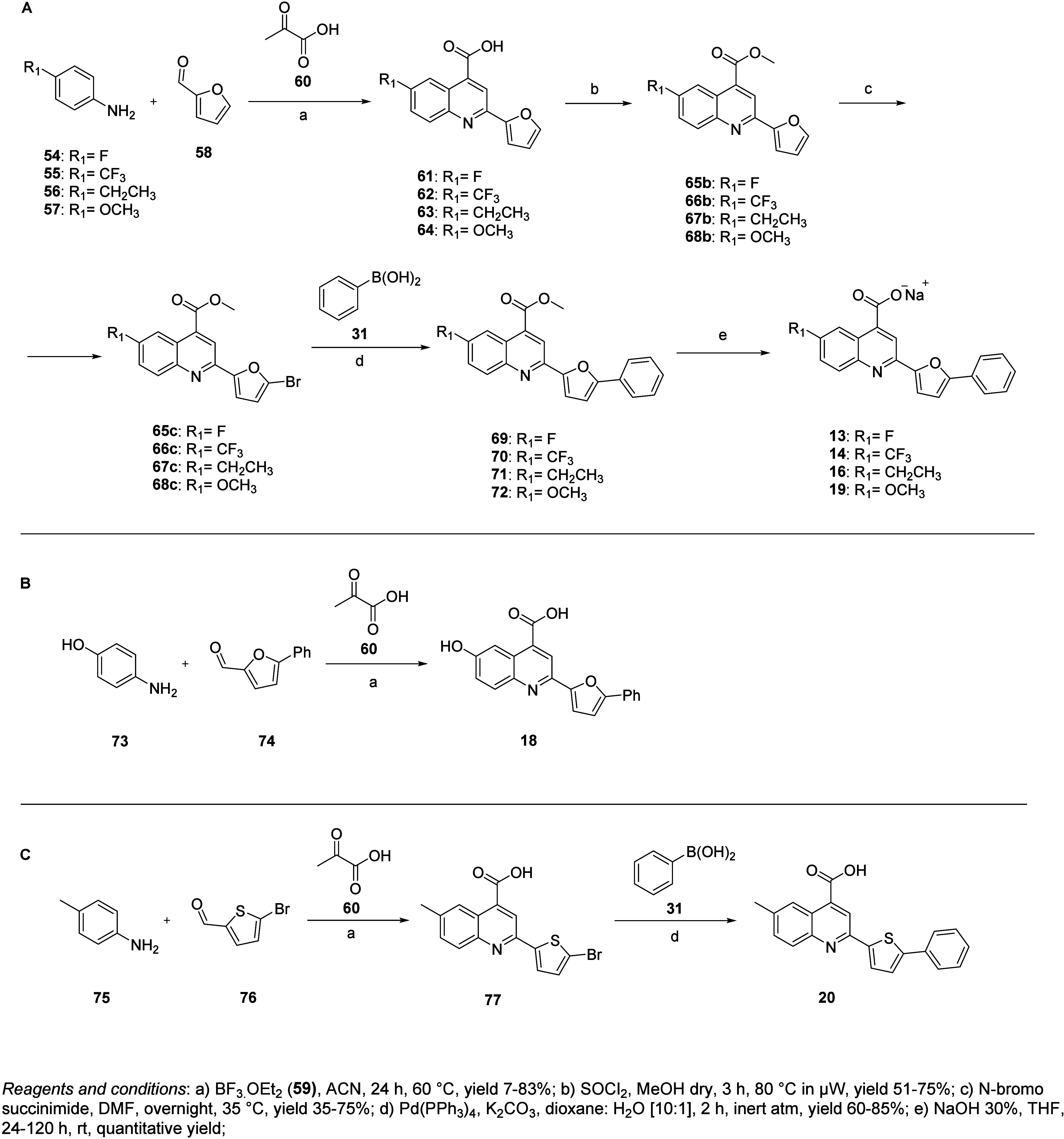
Synthetic Strategy *via* Doebner Reaction for Compounds (A) **13**–**14**, **16** and **19**, (B) **18** and (C) **20**

All new derivatives were first screened in a competitive biochemical ELISA assay (Section S3) and compared to the parent compound **1** (Table 1). This assay is effective in evaluating the ability of new compounds to compete with BRC4 for RAD51.^8^ Compounds showing EC_50_ values below 60 μM progressed to the cell-based HR Quick Assay (HR-QA), a tool for the rapid screening of HR inhibition activity. As a model for cell-based experiments, we selected BxPC-3 cells, which derive from human adenocarcinoma and express functional BRCA2 and high levels of RAD51. Compounds showing >50% HR inhibition were tested for synergism with PARPi ola in BxPC-3 cell viability assays (Table 1). This funnel was purposely designed to allow the identification of compounds able to impair cellular HR through direct RAD51-BRCA2 PPI inhibition.

Initial modifications reorienting carboxylic acid (**2**, **3**) considerably reduced activity or had solubility issues. Conversely, removal of carboxylic acid (**4**) improved HR inhibition (63% at 80 μM), suggesting improved biological activity, but showed only additive effects with ola (Figure S3). EDGs (**5**–**7**) or pyridine substitutions (**9**, **10**) were poorly tolerated or insoluble, while fluorinated derivative **8** retained ELISA activity but had limited HR inhibition (Figure S4). These preliminary results suggested the nonessentiality of the carboxylic group on the phenyl ring A. Subsequently, we removed the methyl group **11** and then we coupled it with the removal of the carboxylic acid of the phenyl ring (**12**). Comparing the activity of **11** and **12** to **4**, we did not observe substantial differences in PPI inhibition, with **12** being just above the EC_50_ cutoff. With these considerations in hand, derivative **4** emerged as the most promising early hit and opened to the development of a set of monocarboxylic acid derivatives. In this second round, the structural tuning focused on position 6 of the quinoline (**13**–**19**). Halogen-containing groups were differently tolerated: indeed, while ELISA activity decreased for fluorine-containing analogues (**13**, **14**), bromine analogue **15** resulted in the second most potent inhibitor of the series. However, **15** lost the race, failing to inhibit cellular HR. The elongation of methyl to ethyl moiety led to **16**, comparable to **4** in ELISA and slightly more potent in inhibiting HR, but additive in cotreatment with ola (Figure S5). The replacement with small EDGs, such as HBA/HBD, always gave micromolar PPI inhibitors (**17**–**19**), including the most potent ELISA inhibitor **18**. In cell-based assays, the primary amine derivative **17** inhibited HR at a lower concentration than previous analogues but was only additive when combined with ola (Figure S6). PPI inhibitor **18**, bearing a hydroxyl group, showed remarkable HR inhibition but no dose-dependent trend, suggesting eventual in-cell off-targets (Figure S7). Methoxy derivative **19**, combined good ELISA activity and the strongest HR inhibition (81% at 80 μM), also showing dose-dependent synergy with ola. Lastly, the substitution of the furan central core with thiophene gave micromolar PPI inhibitor **20**, which strongly inhibited HR but reduced viability alone and with ola, suggesting off-target effects (Figure S8).

Overall, preliminary screening identified compound **19** as the best-in-class PPI inhibitor, effectively inhibiting HR and synergizing with PARPi to reduce cell viability in BxPC-3 cells. Docking studies of **19** into the LFDE pocket of RAD51 suggested a reversed binding pose compared to compound **1**, likely due to the absence of one carboxyl group. As shown in Figure 2A, **19** reorientates to form hydrogen bonds with Arg250 and Arg247 by using its quinolinic ring carboxyl group. It also retains π-π interactions with Tyr205 and introduces an additional π-cation interaction with Arg252 *via* the unsubstituted phenyl ring (Figure 2A). Notably, calculations from computational point mutational studies revealed that the key residues involved in the protein–ligand interaction are Arg250, Met251, and Arg254, with Ser208 and Arg247 playing a lesser role. Indeed, mutations at these positions tend to weaken ligand binding to the protein. Conversely, substitutions such as Asp257→Trp, Leu204→Arg, and Ser208→Arg may potentially enhance the binding affinity (Table S1).

**Figure 2:**
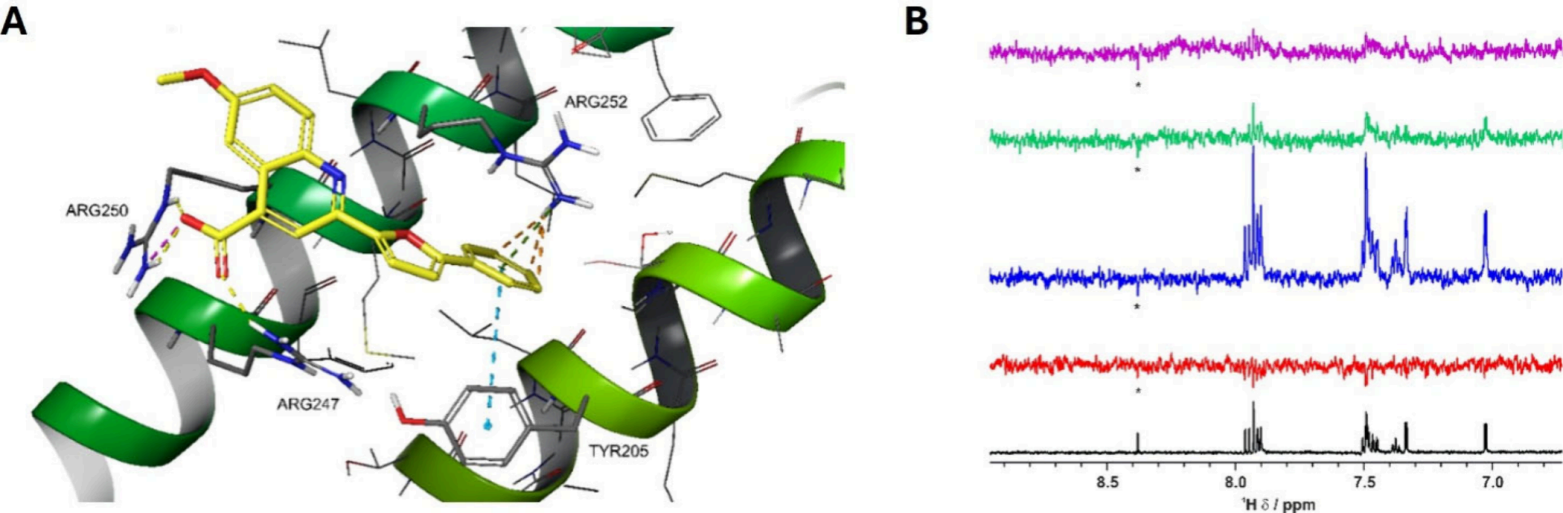
**A**) **19** docked into the LFDE binding site (Zone II) of the RAD51 crystal structure (PDB ID 1N0W); **B**) ^1^H 1D spectrum of compound **19** (50 μM, black). WaterLOGSY spectrum of compound **19** in the absence (red) and in the presence of 1 μM RAD51 (blue), and in the presence of 1 μM RAD51 plus 2 μM (green) or 5 μM (violet) BRC4.

Microscale thermophoresis (MST) on recombinant human RAD51 determined a dissociation constant (K_d_) for the RAD51-**19** interaction of 75.14 μM (Figure S9), aligning with its micromolar ELISA EC_50_. Furthermore, NMR analyses confirmed that **19** binds to RAD51 and is displaced upon the addition of BRC4, suggesting that its binding site lies at the PPI interface (Figure 2B). Encouraged also by a high kinetic solubility (>250 μM in PBS buffer), we selected **19** for further biological studies to characterize its cellular mechanism of action.

First, **19** showed a clear dose–response trend in a multiple-dose HR-QA (IC_50_ = 21.74 ± 1.81 μM) (Figure 3A). To further confirm its impact on HR, we performed the m-Clover Lamin A assay (mCl-HR) at 10–60 μM in HEK-293 cells, chosen for their high transfection efficiency and proliferation rate.^23^ While ∼20% of untreated cells were mClover-positive, 40 μM **19** reduced to ∼4%, indicating ∼74% HR inhibition, aligning with HR-QA and further supporting **19** as an HR inhibitor (Figure 3B–C).

**Figure 3:**
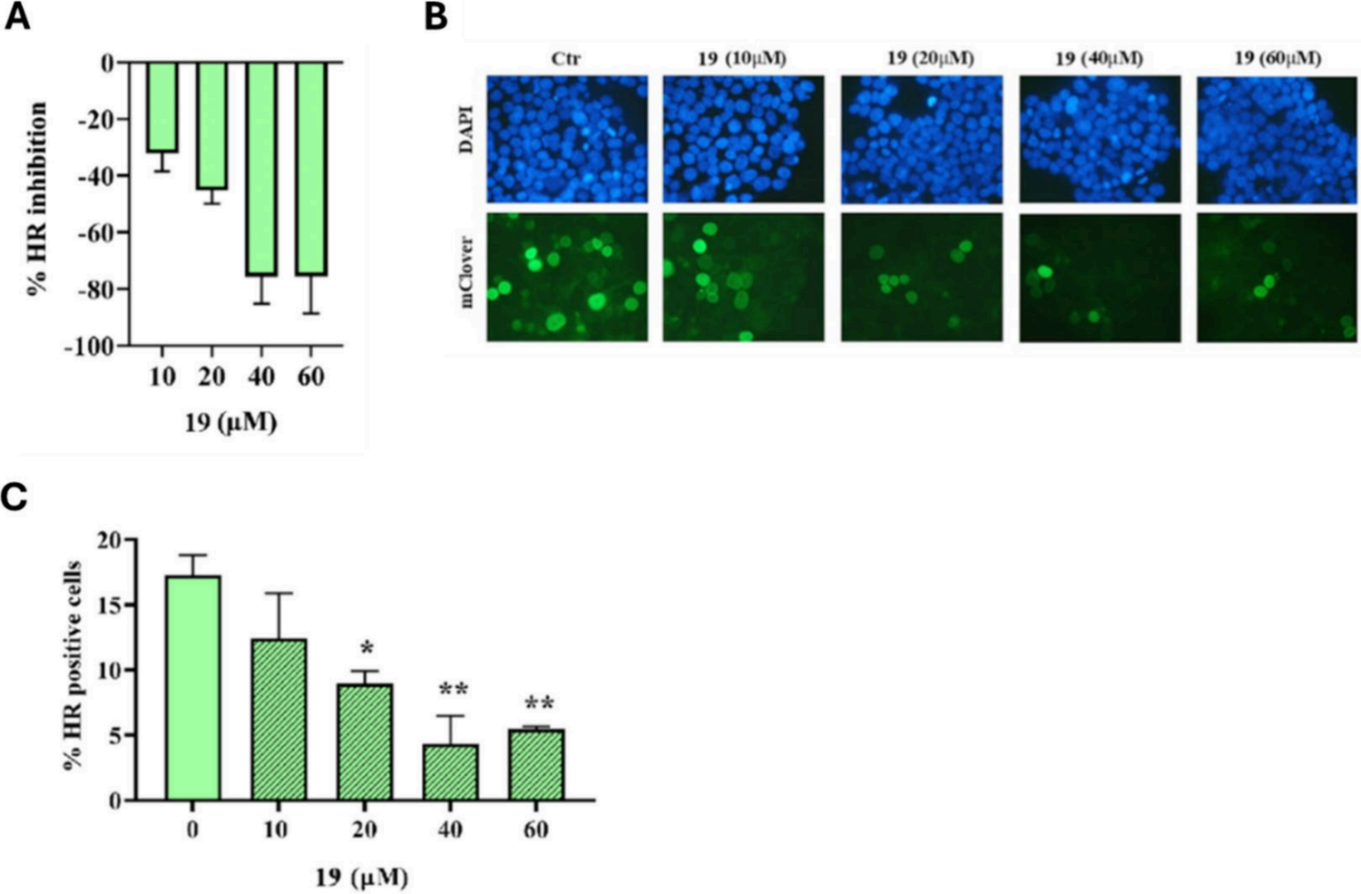
Evaluation of HR inhibition by increasing the doses of **19**. **A**) HR Quick Assay (HR-QA); **B**) Representative images of fluorescent mClover^+^ HEK-293 cells, untreated controls (Ctr), and DAPI-stained nuclei; **C**) Percentage of HR-proficient (mClover^+^) cells in untreated and **19**-treated samples.

Since BRCA2-mediated RAD51 recruitment to the nucleus is essential for DNA repair by HR,^24–26^ we evaluated whether **19** disrupts RAD51 foci formation after DNA damage. Upon cisplatin (CPL) treatment, **19** significantly reduced RAD51 foci formation in BxPC-3 cells, supporting the proposed mechanism of action (Figure S10A–B).

To test whether compound **19** could pharmacologically induce SL, we evaluated the effect of its combination with ola. Since **19** inhibits HR and ola blocks SSB repair, their combined effect is expected to severely disrupt DNA repair and trigger apoptosis. We first assessed DNA damage using immunofluorescence to detect H2AX phosphorylation (γ-H2AX) and micronuclei, established markers of genotoxic stress. The **19**/ola combination significantly increased γ-H2AX levels after 72 h compared to ola alone (Figure S10C–D). Similarly, micronuclei formation nearly doubled when combining **19**/ola (Figure S10E–F). These findings supported the hypothesis that **19** enhances the genotoxic impact of PARPi and that the simultaneous disruption of HR and SSB repair results in SL. Furthermore, the **19**/ola combination significantly decreased colony formation, while **19** alone did not affect clonogenicity, highlighting the enhanced therapeutic potential of their coadministration (Figure S11).

**19**/ola combination was tested in different pancreatic cancer models, BxPC-3, HPAC and CAPAN-1 cells. Compared to BxPC-3, HPAC cells do not harbor TP53 mutations,^27,28^ while CAPAN-1 are BRCA2-mutated cells and lack RAD51/BRCA2-dependent HR. According to a 6-day treatment, **19** significantly enhanced the antiproliferative effect of ola in both BxPC-3 (I. Index 0.77) and HPAC (I. Index 0.72) (Figure 4A–B), suggesting the approach may be effective across various PDAC conditions. The I. Index indicated a near-synergistic effect of the combination (Figure S12).^4,29^ In contrast, in CAPAN-1 cells, which do not operate RAD51/BRCA2-mediated HR due to BRCA2 mutation, ola alone had a stronger effect on cell viability as expected, and **19** did not further enhance its effect (I. Index > 0.8, Figure 4C, S12), supporting the specificity of **19** for targeting RAD51/BRCA2 PPI. Importantly, the **19**/ola combination had no significant effect on normal pancreatic epithelial H-6037 cells, indicating a promising level of selectivity toward cancer cells over normal cells (Figure 4D).

**Figure 4:**
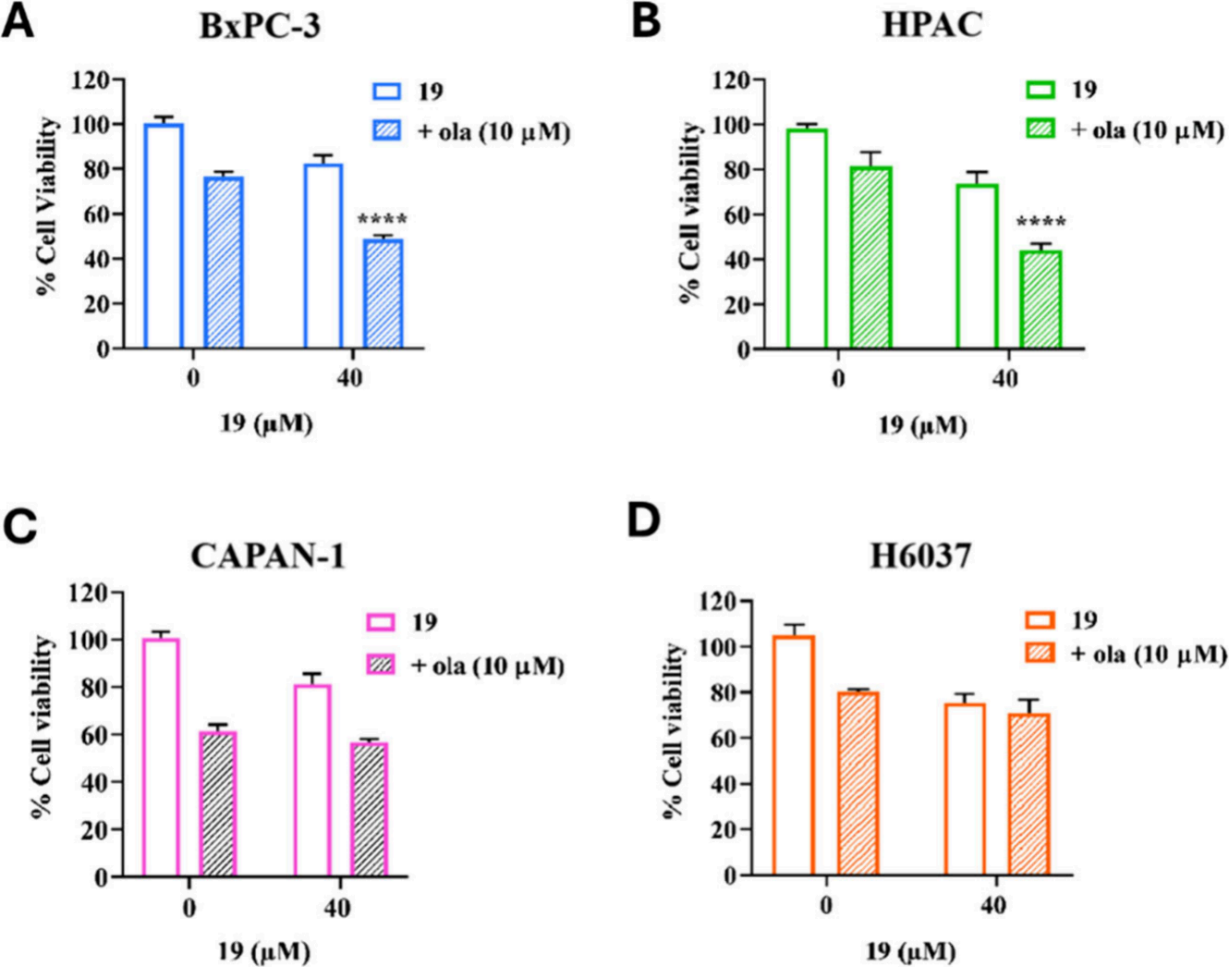
Cell viability of 6-day treatment with **19**, ola, or combination: BxPC-3 (**A**), HPAC (**B**), CAPAN-1 (**C**) and H6037 (**D**).

Following the cell viability results, we verified whether in BxPC-3 and HPAC cells^30^ the **19**/ola combination was effective in inducing cell death. Cells were treated with **19**/ola for 6 days in the presence either of Z-VAD-FMK, a pan-caspase inhibitor expected to prevent cell death by apoptosis (Figure 5A, D), or Necrostatin-1, a necroptosis inhibitor (Figure 5B, E). In both cell cultures, only Z-VAD-FMK was found to significantly restore cell viability, compared to untreated cells, while no statistically significant effect was observed in cells exposed to Necrostatin-1. A further confirmation of apoptotic cell death was searched by the immunoblotting detection of BAX/BCL-2 ratios.^31,32^ As shown in Figure 5C, F, a significantly increased BAX/BCL2 ratio was observed in both cell lines, further supporting the induction of SL by **19**/ola cotreatment.

**Figure 5:**
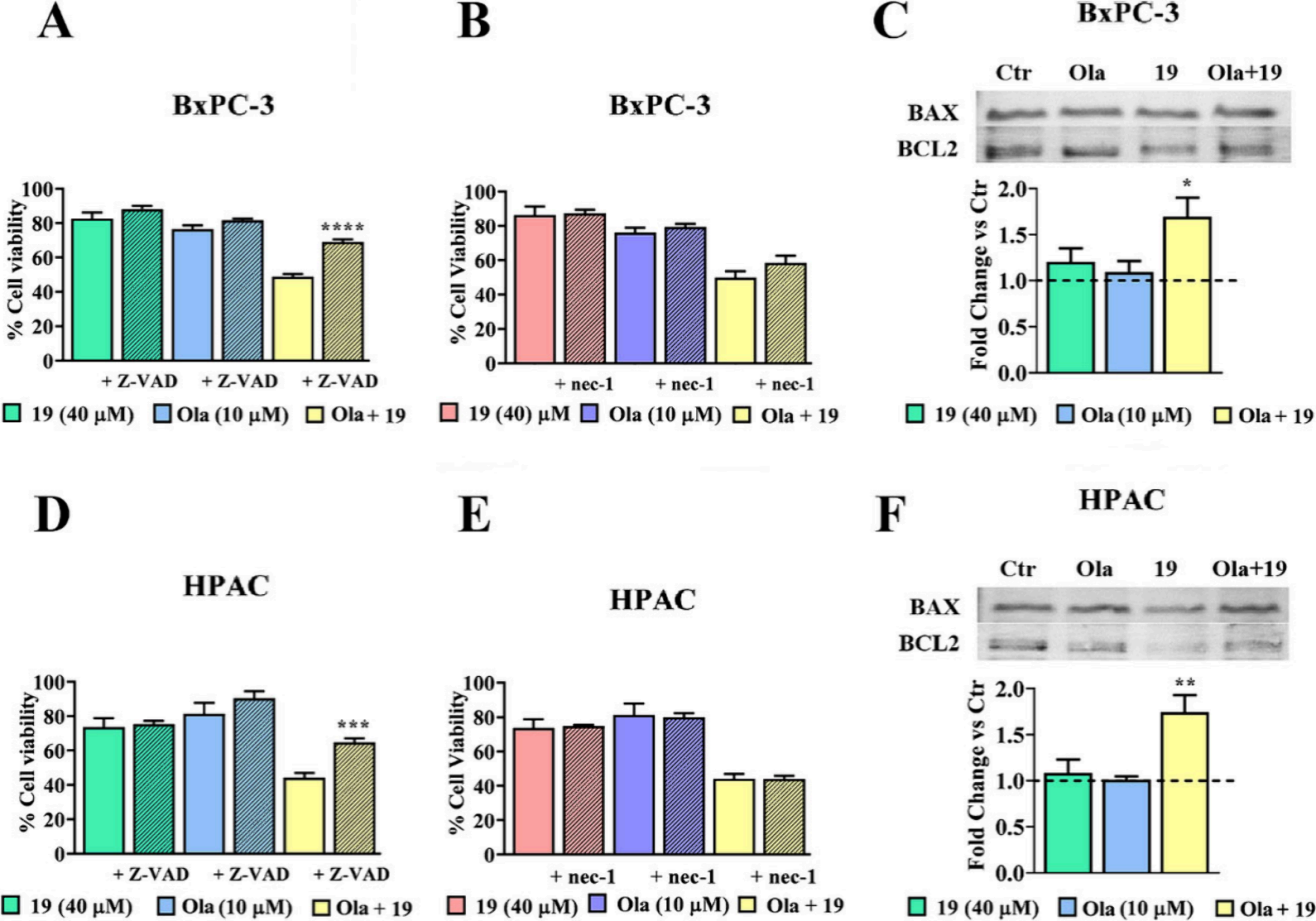
Cell death assays performed with Z-VAD-FMK (**A**, **D**) or Necrostatin-1 (**B**, **E**) in cultures treated with **19**, ola, or their combination; (**C**, **F**) Apoptosis induction confirmed by immunodetection of the BAX/BCL2 ratio.

Finally, we examined the effects of the **19**/ola combination in 3D spheroid cultures of BxPC-3 cells, which better mimic *in vivo* conditions compared to 2D cultures for their differences in cell–cell interactions, mechanical properties and drug diffusion.^33,34^ In this model, we observed evidence of synergism for the combination of ola with 60 μM **19** (I. Index 3D = 0.85) (Figure 6A). These results align with the literature, suggesting that 3D models are more resistant to cytotoxicity due to increased drug resistance and limited drug penetration.^35,36^ Furthermore, we analyzed spheroid volume over time during treatment with **19** alone or in combination with ola (Figure 6B–C). Interestingly, 60 μM **19** alone had no significant impact on spheroid volume, but its combination with ola significantly reduced the spheroid volume. The cell death analysis showed significantly increased spheroid death with the **19**/ola combination, mirroring the spheroid volume findings (Figure 6D). These results indicate that while 40 μM **19** synergized with ola in 2D cultures, the combination of 60 μM **19** and ola retained synergism in 3D cultures.

**Figure 6:**
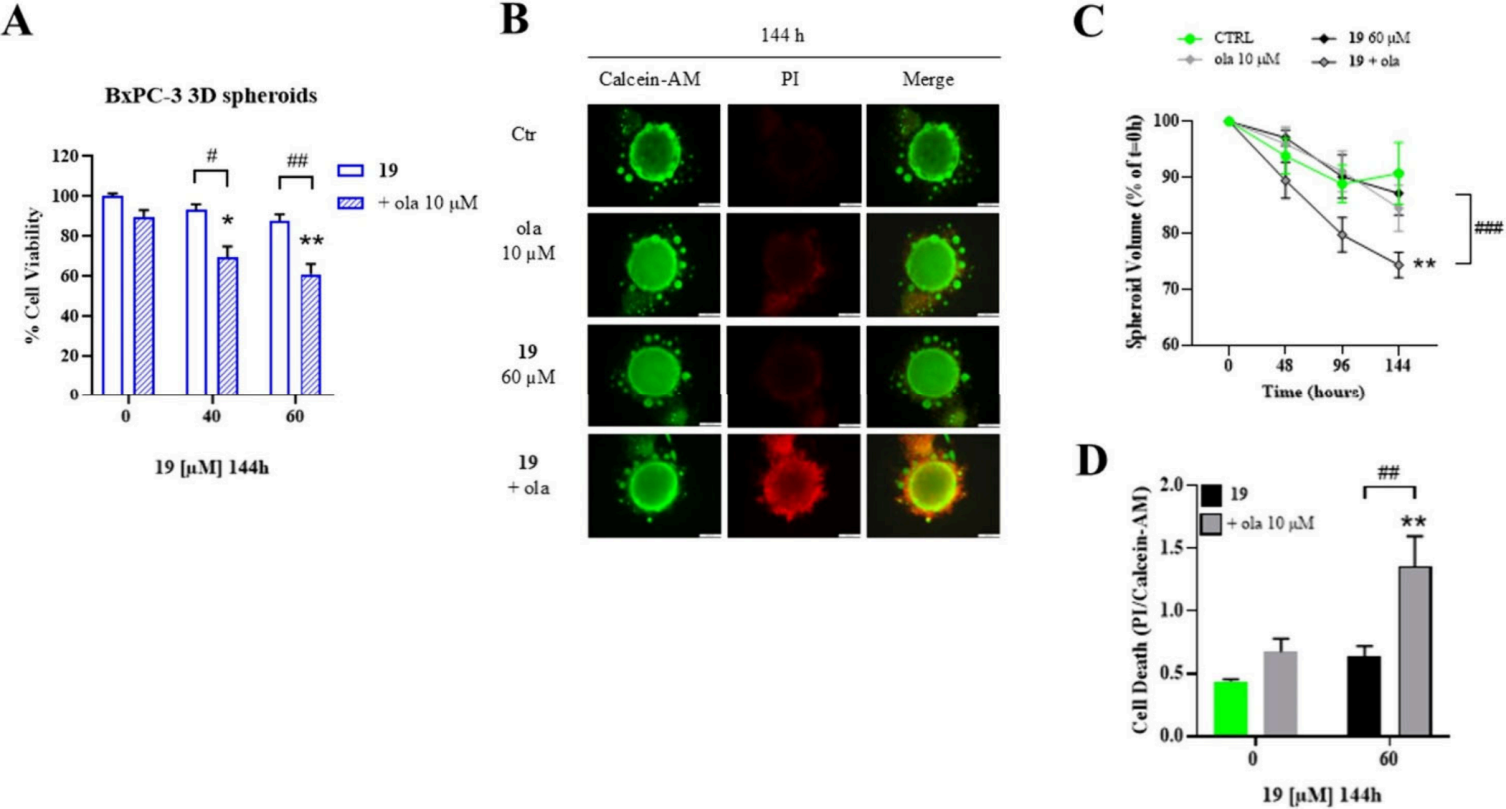
Effects of 6-day treatment with **19** and ola, alone or in combination, on BxPC-3 3D spheroid viability (**A**), volume (**B**, **C**), and cell death (**D**).

Comparative analysis with previously reported RAD51–BRCA2 inhibitors, **35d** and **CAM833**, highlights key differences in solubility and activity profiles (Table S2). Compound **19** displays a higher ELISA EC_50_ (29.35 ± 2.25 μM) than **35d** (20 ± 1.1 μM) and **CAM833** (10 ± 2 μM), yet it shows a dramatically improved kinetic solubility (>250 μM vs <1 μM for **35d**). This represents a substantial advance over previous Zone II inhibitors, which were limited by poor aqueous solubility.

In functional HR assays, **19** achieves 74% inhibition at 40 μM, comparable to **35d** (67% at 40 μM) and close to **CAM833** (>90% at 50 μM). Notably, under the same conditions where **19** and olaparib displayed synergistic behavior in BxPC-3 cells, **CAM833** showed only a borderline, nearly insignificant synergism (Figure S13). Although **CAM833** remains a reference RAD51–BRCA2 inhibitor, in pancreatic cancer models **19** exhibits a more favorable balance between potency and solubility (I. Index = 0.54), representing a significant improvement relative to **35d**. Collectively, these results identify **19** as an improved chemotype among RAD51–BRCA2 PPI inhibitors, combining solid biochemical activity with markedly enhanced solubility and synergistic cellular effects.

In conclusion, we reported phenylfuran-4-carboxyquinoline as a promising chemotype for the development of RAD51-BRCA2 PPI inhibitors. Within the series, **19** emerged as a representative tool compound, overcoming the aqueous solubility limitations of previous inhibitors and displaying selectivity toward cancer cells over normal pancreatic cells. Computational point mutation analysis and NMR displacement assays supported its mechanism as a PPI inhibitor, in agreement with its biological activity as an HR inhibitor. The combination of **19** with ola specifically induced apoptosis through the simultaneous disruption of two distinct DNA repair mechanisms, consistent with the SL paradigm. As the activity of **19** remains in the micromolar range, further optimization is currently ongoing. This early stage work provides a validated starting point for future structure refinement and contributes to the expanding chemical biology toolkit for probing the RAD51-BRCA2 interaction in pancreatic cancer models.

## Supplementary Material



## Notes

Computational studies; Synthetic procedures; NMR spectra and UPLC-MS of **1**–**20**; Biophysical, biochemical and biological methods. (PDF)

## Abbreviations

DDR: DNA damage response
EDG: electron-donating group
HBA: hydrogen bond acceptor
HBD: hydrogen bond donor
HR: homologous recombination
HRD: homologous recombination deficiency
HR-QA: HR Quick Assay
MST: microscale thermophoresis
PARPi: poly­(ADP-ribose) polymerase inhibitor
PPI: protein–protein interaction
SL: synthetic lethality, synthetic lethal
*TP53*: Tumor Protein 53
VS: virtual screening
