# Supplementary material for: Targeting RAD51-BRCA2 Interaction to Enhance Synthetic Lethality with Olaparib in Pancreatic Cancer: Development of a Novel Phenyl Furan-Quinoline-Carboxylic Acid Series
Source: ACS Med Chem Lett. 2026 Jan 26;17(2):520–30. doi: 10.1021/acsmedchemlett.5c00711 (PMC12907944; doi:10.1021/acsmedchemlett.5c00711)
Supplement: Supplementary file 1 [file ml5c00711_si_001.pdf]

Targeting RAD51-BRCA2 Interaction to Enhance Synthetic Lethality with Olaparib in Pancreatic Cancer: Development of a Novel Phenyl Furan-Quinoline-Carboxylic Acid Series

Giovanni Ferrandi,[1,2,±] Greta Bagnolini,[1,±] Laura Poppi,[2,±] Mirco Masi,[2] Viola Previtali,[2] Angela Andonaia,[2] Giulia Varignani,[1,2] Marina Veronesi,[5] Francesca De Franco,[3] Federico Falchi,[1,2] Giuseppina Di Stefano,[4] Stefania Girotto,[5] Marinella Roberti,[1, §,*] Andrea Cavalli[1,2,6, §,*]

[1] Department of Pharmacy and Biotechnology, University of Bologna, 40126 Bologna, Italy
[2] Computational and Chemical Biology, Istituto Italiano di Tecnologia, 16163 Genoa, Italy
[3] TES Pharma S.r.l., I-06073 Corciano, Perugia, Italy
[4] Department of Medical and Surgical Sciences, University of Bologna,40126 Bologna, Italy
[5] Structural Biophysics Facility, Istituto Italiano di Tecnologia, 16163 Genoa, Italy
[6] Centre Européen de Calcul Atomique et Moléculaire (CECAM), Ecole Polytechnique Fédérale de Lausanne (EPFL), 1015 Lausanne, Switzerland.

[±]Authors equally contributed to this work as co-first
[§]Authors equally contributed to this work as co-last
* Corresponding author e-mail: marinella.roberti@unibo.it

Contents

S1. Computational studies 3

Virtual Screening 3

Figure S1 Chemical structures of hit compound **ARN22064** and its derivative **ARN24089** 4

Point mutational studies 4

Table S1. Point mutations and delta affinity values 4

S2. Chemistry 6

Materials and methods 6

Synthetic general procedures and synthesis of final compounds **1**–**20** and intermediates **24**, **25**, **26b**–**27b**, **26c**–**27c**, **38**–**49**, **53**, **61**–**64, 65b**–**68b**, **65c**–**68c**, **69**–**72**, **77** 6

Scheme S1. Synthesis of intermediate **52** 21

Scheme S2. Synthesis of intermediate **74** 22

Kinetic solubility studies 22

S3. Biophysical, biochemical and biological experiments 23

Materials and methods 23

Figure S2. HR inhibition of **1** and cell viability of **1**/ola combination 31

Figure S3. HR inhibition of **4** and cell viability of **4**/ola combination 31

Figure S4. HR inhibition of **8** 32

Figure S5. HR inhibition of **16** and cell viability of **16**/ola combination 32

Figure S6. HR inhibition of **17** and cell viability of **17**/ola combination 33

Figure S7. HR inhibition of **18** 33

Figure S8. HR inhibition of **20** and cell viability of **20**/ola combination 34

Figure S9. MST analysis of **19** binding to RAD51 34

Figure S10. Evaluation of RAD51 foci, γ-H2AX nuclei and micronuclei formation. 35

Figure S11. Evaluation of colony formation of **19**/ola combination 36

Figure S12. Interaction Index of **19**/ola combination in BxPC-3, HPAC and CAPAN-1 36

Table S2. Comparative analysis of **19** with **35d** and **CAM833** 37

Figure S13. Cell viability of **CAM833**, ola, or combination in BxPC-3 cell 37

S4. ^{1}H-NMR spectrum, ^{13}C-NMR spectrum and UPLC-MS analysis of final compounds **1-20** 38

References 78

S1. Computational studies

Virtual Screening

For the sake of clarity, we report below the sections “1.1 Protein Preparation”, “1.2 Database Preparation” and “1.3 High Throughput Docking (Virtual Screening)”, as we have previously reported in G. Bagnolini, D. Milano, M. Manerba, F. Schipani, J. A. Ortega, D. Gioia, F. Falchi, A. Balboni, F. Farabegoli, F. De Franco, J. Robertson, R. Pellicciari, I. Pallavicini, S. Peri, S. Minucci, S. Girotto, G. Di Stefano, M. Roberti, A. Cavalli, J. Med. Chem. **2020** 63 (5), 2588-2619 DOI: 10.1021/acs.jmedchem.9b01526[1]

Protein Preparation

The crystal structure of a RAD51-BRCA2 BRC repeat complex was downloaded from the Protein Data Bank (PDB code 1N0W). The structure was then treated with the Schrödinger Suite 2014-4 Protein Preparation Wizard tool. All the selenomethionines were mutated to methionine, water molecules and ions were removed, and an exhaustive sampling of the orientations of groups, whose hydrogen bonding network needs to be optimized, was performed. Finally, the protein structure was refined to relieve steric clashes with a restrained minimization with the OPLS2005 force field until a final RMSD of 0.30 Å with respect to the input protein coordinates.

Database Preparation

A commercially available library of compounds composed of ASINEX and LifeChemicals databases collected from ZINC was prepared with the LigPrep tool of the Schrödinger Suite. The 2D (smi file) structures were converted to 3D structures and for each entry all stereoisomers were generated. The resulting molecules were submitted to Epik and all the tautomers and ionization states at pH 7.0 ± 2.0 were calculated. Finally, duplicates, compounds with more than 2 chiral centers, Pan-Assay Interference Compounds (PAINS), compounds with Michael acceptor groups, and frequent hitters were deleted. To enrich the database with potential Protein Protein Interaction Inhibitors, the database was filtered with the PPI-HitProfiler tool using the “soft” methods.

High Throughput Docking (Virtual Screening)

All filtered ligands (about 750K) were docked with Glide SP by centering the grid on the position of BRCA Phe1546. The 10K top-scoring compounds were re-docked with Glide XP and the 1K top-scoring compounds were selected. Both grid generation and docking calculations were performed with the default settings. The selected compounds were visually inspected to identify compounds able to match the interactions between RAD51 and BRCA and 42 compounds were selected and purchased.

Induced-Fit Docking IFD (Induced-Fit Docking)

Calculations were performed with the previously prepared protein structure using both enantiomers of each ligand. All the ligands were prepared using Ligprep utility in Schrödinger 2019-2. The IFD protocol involves the use of the Glide docking program to generate a number of initial possible ligand poses followed by a protein side chain optimization using the S3 Prime protein structure modeling program. After a number of iterations, the process produces a list of final poses, ordered by a proprietary scoring function (that is a combination of the Prime Energy and Glide scoring function). The Schrödinger Extended Sampling protocol was selected along with the OPLS3e force field. The grid box was centered on the centroid of residues Tyr205, Arg247, and Phe259. Residues within 5.0 Å of ligand poses were refined during the process. The other parameters were set to their default values. Resulting poses were evaluated by visual inspection.

Figure S1 Chemical structures of hit compound **ARN22064** and its derivative **ARN24089**

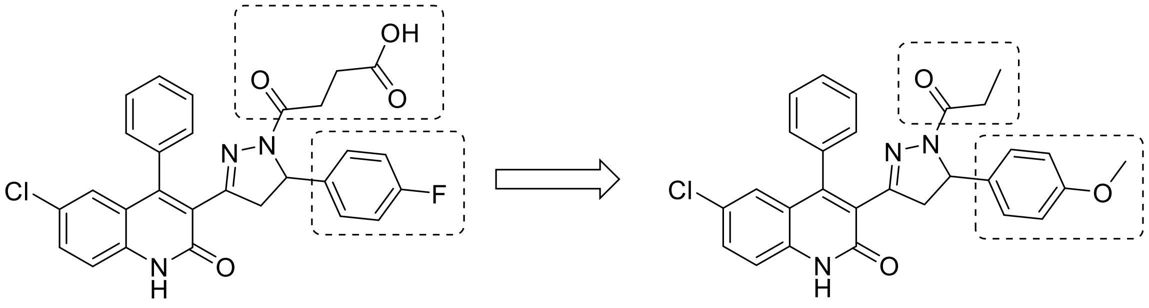

ARN22064 ARN24089

Hit compound **ARN22064** was identified by VS against PDB ID 1N0W, and its derivative **ARN24089** was published in Bagnolini G. *et al.* J.Med.Chem, **2020**, 63 (5), 2588-2619.[1]

Point mutational studies

Starting from the protein–ligand complex obtained by molecular docking, all residues within a 6 Å radius from the ligand **19** were mutated using the *Residue Scanning Calculation* tool available in the Schrödinger Suite (release 2024-4). Each residue was mutated to all possible amino acids except histidine. All settings were kept at their default values.

Table S1. Point mutations and delta affinity values

The table shows mutations that could lead to a significant variation in binding affinity. For simplicity, all results with a ΔAffinity between –4 and +4 were omitted. Positive values indicate a substantial loss of affinity, whereas negative values suggest a potential improvement in affinity.

Mutation	Delta Affinity
250(ARG->GLY)	15.5
250(ARG->ASP)	14.9
254(ARG->ASP)	14.1

250(ARG->GLU)	14.1
250(ARG->ASN)	13.3
250(ARG->ALA)	12.6
250(ARG->SER)	12.6
251(MET->ASP)	12.3
254(ARG->GLY)	12.0
250(ARG->CYS)	11.7
251(MET->GLY)	11.2
250(ARG->PRO)	10.7
251(MET->TRP)	10.5
251(MET->LYS)	10.3
250(ARG->GLN)	10.2
250(ARG->THR)	10.1
254(ARG->ALA)	9.7
251(MET->ILE)	9.7
254(ARG->PRO)	9.7
254(ARG->VAL)	9.5
254(ARG->GLU)	9.5
254(ARG->LYS)	9.5
251(MET->ALA)	9.1
251(MET->SER)	8.8
254(ARG->CYS)	8.3
251(MET->GLU)	8.3
250(ARG->TRP)	8.1
251(MET->ASN)	8.0
251(MET->CYS)	7.9
250(ARG->VAL)	7.7
250(ARG->MET)	7.3
250(ARG->LYS)	7.1
251(MET->VAL)	7.1
250(ARG->ILE)	7.0
250(ARG->LEU)	6.9
208(SER->GLU)	6.5
254(ARG->GLN)	6.4
254(ARG->ILE)	5.8
251(MET->TYR)	5.8
250(ARG->PHE)	5.6
251(MET->THR)	5.5
250(ARG->TYR)	5.3
251(MET->LEU)	5.1
251(MET->PHE)	5.1
247(ARG->GLU)	4.9
205(TYR->ASP)	4.9
255(LEU->ASP)	4.8
254(ARG->ASN)	4.6
255(LEU->PHE)	4.6
205(TYR->GLU)	4.1
257(ASP->TRP)	-4.2
204(LEU->ARG)	-5.7
208(SER->ARG)	-7.1

S2. Chemistry

Materials and methods

Solvents and reagents were obtained from commercial suppliers and used without further purification. NMR experiments were run on a Bruker Avance III 400 MHz spectrometer (400.13 MHz for ^{1}H and 100.62 MHz for ^{13}C), equipped with a BBI probe and Z-gradients, or on a Bruker FT NMR Avance III 600-MHz spectrometer (600.130 MHz for 1H and 150.903 MHz for 13C) equipped with a 5 mm CryoProbe QCI quadruple resonance, a shielded Z-gradient coil, and the automatic sample changer SampleJet NMR system. Spectra were acquired at 300 K, using deuterated dimethylsulfoxide (DMSO-d_6) or deuterated chloroform ($CDCl_3$-*d*) as solvents. Chemical shifts for ^{1}H and ^{13}C spectra were recorded in parts per million using the residual nondeuterated solvent as the internal standard. The coupling constants of the splitting patterns were reported in Hz and were indicated according i.e. as singlet (s), doublet (d), triplet (t) or multiplet (m). UHPLC−MS analyses were run on a Waters ACQUITY ARC UHPLC/MS system consisting of a QDa mass spectrometer equipped with an electrospray ionization interface and a 2489 UV/Vis detector. The detected wavelengths (l) were 254 nm and 365 nm. The analyses were performed on an XBridge BEH C18 column (10 × 2.1 mm i.d., particle size 2.5 μm) with a XBridge BEH C18 VanGuard Cartridge precolumn (5 mm × 2.1 mm i.d., particle size 1.8 μm). The mobile phases were H_2O (0.1% formic acid, FA) (A) and MeCN (0.1% formic acid) (B). Electrospray ionization in positive and negative mode was applied in the mass scan range 50−1200 M/z. The following gradient was used: 0−0.78 min, 20% B; 0.78-2.87 min, 20−95% B; 2.87-3.54 min, 95% B; 3.54-3.65 min, 95-20% B; 3.65-5-73, 20% B. Flow rate: 0.7 mL/min. All final compounds displayed ≥95 % purity as determined by UPLC/MS analysis. Electrospray ionization (ESI) high-resolution mass spectra (HRMS) were obtained on an XevoG2XSQTof mass spectrometer, solvent MeOH +0.1% FA, acquisition time 30 sec.

Synthetic general procedures and synthesis of final compounds **1**–**20** and intermediates **24**, **25**, **26b**–**27b**, **26c**–**27c**, **38**–**49**, **53**, **61**–**64, 65b**–**68b**, **65c**–**68c**, **69**–**72**, **77**

*General procedure A for the synthesis of 2-(furan-2-yl)quinoline-4-carboxylic acid derivatives **15, 24, 25, 53**.* In a microwavable vessel, the appropriate substituted isatin (**21, 22, 50, 51**, 1.00 equiv), acetylfuran (**23, 52**, 1.20 equiv) and KOH (3.00 equiv) are stirred in EtOH/H_2O (3:1 v/v, 0.5 M) at room temperature. The reaction is conducted in the microwave reactor and kept stirring at 80 °C for 3 hours, 80 W. After 3 hours, the solution is transferred to a flask and is quenched with water. The ethanol is evaporated under reduced pressure and the aqueous solution is extracted with Et_2O (3x50 mL) to remove the excess of furan. The aqueous phase is then acidified to pH 3 with conc. HCl until the precipitation of the desired product. The residue is filtered, resolubilized with acetone and concentrated in vacuum to obtain the pure product without further purifications.

*General procedure B for the synthesis of methyl 2-(furan-2-yl)quinoline-4-carboxylate derivatives **26b**, **27b**, **65b**–**68b**.* In a microwavable vessel, $SOCl_2$ (3.00 equiv) is added dropwise to a solution of the quinoline (**24, 25, 61–64,** 1.00 equiv) in dry MeOH (0.4 M) at 0 °C under stirring. The tube is placed in the microwave reactor and the reaction is stirred for 3 hours at 80 °C, 80 W. The reaction is cooled to room temperature and 10 mL of MeOH are added and evaporated by rotavapor with basic trap. The residue is dissolved in DCM and washed with K_2CO_3 20% (3x40 mL) and brine (1x40 mL), dried over Na_2SO_4, filtered and concentrated under reduced pressure to obtain the pure product without further purifications.

*General procedure C for the synthesis of methyl 2-(5-bromofuran-2-yl)quinoline-4-carboxylate derivatives **26c**, **27c**, **65c**–**68c**.* In a microwavable vessel, a solution of the appropriate ester intermediate (**26b**, **27b, 65–68b,** 1.00 equiv) is stirred in DMF (0.4 M) prior to the addition of *N*-bromo succinimide (1.50 equiv) at room temperature. The tube is then placed in the microwave reactor and kept under stirring at 35 °C for 40 minutes, 80 W. The reaction mixture is added dropwise to 20 mL of cold distilled water at 0°C under stirring. The formation of a precipitate is observed, and the solid is filtered. The crude product is purified over silica gel to obtain the pure compound, unless otherwise noted.

*General procedure D for the synthesis of methyl 2-(5-arylfuran-2-yl)quinoline-4-carboxylate derivatives **20**, **38–49**, **69–72**.* In a three-neck flask, the brominated intermediate (**26c–27c, 65c–68c, 77,** 1.00 equiv) is solubilized in a mixture of dioxane: H_2O [10:1] 0.1 M. The appropriate boronic acid **28–37** (1.50 equiv) and K_2CO_3 (3.00 equiv) are added to the solution and the mixture is degassed with N_2. Palladium tetrakis (0.05 equiv) is then added and the reaction is conducted at 100 °C in inert atmosphere, stirring for 2 hours. Then, the reaction is cooled to room temperature, 5 mL of water are added and the dioxane is evaporated under reduced pressure. The formed precipitate is recovered by filtration. The remaining water is extracted with AcOEt (3x10 mL). The organic phase is collected, dried over Na_2SO_4, filtered and concentrated under vacuum. The crude joints solids are purified over silica gel to obtain the pure compound, unless otherwise noted.

*General procedure E for the synthesis of 2-(5-arylfuran-2-yl)quinoline-4-carboxylic acid derivatives **1–16**, **19**.* The intermediate **38–49, 69–72** (1.00 equiv) is solubilized in THF (0.2 M), then NaOH 30% (20.00 equiv) is added, and the reaction is stirred at room temperature until complete conversion. The mixture is then concentrated under reduced pressure and 5 mL of distilled water are added. The formed precipitate is recovered by filtration, and the pure compound is obtained as carboxylate. For water-soluble compounds, the aqueous solution is acidified by the addition of conc HCl to reach pH 3. The red precipitate is recovered by filtration, and the pure compound is obtained as undissociated carboxylic acid.

*General procedure F for the synthesis of 2-(furan-2-yl)quinoline-4-carboxylic acid derivatives **18**, **61–64**, **77*** In a one-neck flask, the appropriate aldehyde (**58, 74, 76,** 2.00 equiv) and boron-diethyl ether trifluoride (**59,** 0.50 equiv) are added to a solution of the appropriate substituted aniline (**54–57, 74, 76,** 1.80 equiv) in dry acetonitrile (0.2 M). The mixture is stirred at 65°C for 20 min. Subsequently, a solution of pyruvic acid **60** (1.00 equiv) in acetonitrile (0.6 M) is added dropwise. The reaction is stirred at 65 °C for 24 hours. Then the

mixture is cooled down to room temperature, water is added, and the aqueous phase is extracted with DCM (3x40 mL). The collected organic phase is additionally extracted with an aqueous NaOH 5% solution (3x20 mL). The basic water is adjusted to pH 3 with conc HCl until the formation of a precipitate. The solid residue is recovered by filtration and dried by vacuum to obtain the pure product without further purifications.

2-(5-(2-Carboxyphenyl)furan-2-yl)-6-methylquinoline-4-carboxylic acid (**1**) (Scheme 1A) Compound **1** was obtained following the described procedure E using intermediate **38** (128 mg, 0.32 mmol) and NaOH 30% (0.85 ml, 6.38 mmol). After acid precipitation, **1** was obtained as pure compound (118 mg, quantitative yield) ^{1}H-NMR (401 MHz, DMSO-*d$_6$*) δ 8.35 (s, 1H), 8.21 (s, 1H), 7.96 (d, J = 8.6 Hz, 1H), 7.79 (d, J = 7.7 Hz, 1H), 7.68 – 7.56 (m, 3H), 7.50 – 7.41 (m, 2H), 6.98 (d, J = 3.6 Hz, 1H), 2.51 (s, 3H). ^{13}C-NMR (101 MHz, CD_3OD-d4) δ 180.47, 178.97, 175.03, 155.54, 153.47, 149.38, 149.26, 148.10, 140.99, 137.48, 133.24, 128.81, 128.73, 128.51, 127.64, 127.11, 126.76, 125.30, 116.07, 113.52, 111.77, 24.24. In agreement with Horak, Y. *et al*.[2] UHPLC-MS (ESI, m/z) Rt = 2.73 min, 374.2 $[M+H]^+$. ESI-HRMS calculated for $C_{22}H_{16}NO_5$ $[M+H]^+$ 374.1023, found 374.1018; ESI-HRMS calculated for $C_{22}H_{14}NO_5$ $[M-H]^-$ 372.0877, found 372.0875.

2-(5-(3-Carboxyphenyl)furan-2-yl)-6-methylquinoline-4-carboxylic acid (2) (Scheme 1A) Compound **2** was obtained via general procedure E using intermediate **39** (186 mg, 0.46 mmol) and NaOH 30% (1.24 ml, 9.2 mmol). After acidic precipitation, **2** was obtained as pure compound (170 mg, quantitative yield). ^{1}H-NMR (401 MHz, DMSO-*d$_6$*) δ 8.39 - 8.34 (m, 2H), 8.32 (s, 1H), 8.16 - 8.08 (m, 1H), 8.00 (d, J = 8.6 Hz, 1H), 7. 90 (dt, J = 7.7, 1.4 Hz, 1H), 7.69 - 7.57 (m, 2H), 7.52 (d, J = 3.6 Hz, 1H), 7.33 (d, J = 3.6 Hz, 1H), 2. 51 (d, J = 1.0 Hz, 3H); ^{13}C-NMR (101 MHz, DMSO-*d$_6$*) δ 167.84, 167.40, 154.24, 152.84, 147.39, 147.26, 137.90, 137.28, 133.04, 132.11, 130.44, 129.95, 129.51, 129.30, 128.66, 124.74, 123.79, 118.41, 113.82, 110.20, 22.05. UHPLC-MS (ESI, m/z) R_t = 2.87 min, 374 $[M+H]^+$ ESI-HRMS calculated for $C_{22}H_{16}NO_5$ $[M+H]^+$ 374.1023, found 374.1017.

2-(5-(4-Carboxyphenyl)furan-2-yl)-6-methylquinoline-4-carboxylic acid (3) (Scheme 1A) Compound **3** was obtained via general procedure E using intermediate **40** (150 mg, 0.373 mmol) and NaOH 30% (1 ml, 7.47 mmol). After acidic precipitation, **3** was obtained as pure compound (139 mg, quantitative yield). ^{1}H-NMR (401 MHz, DMSO-*d$_6$*) δ 8.36 (s, 1H), 7.91 (d, J = 8.1 Hz, 2H), 7.87 (s, 1H), 7.80 (d, J = 8.6 Hz, 1H), 7. 73 (d, J = 8.1 Hz, 2H), 7.46 (dd, J = 8.6, 2.0 Hz, 1H), 7.30 (d, J = 3.5 Hz, 1H), 7.11 (d, J = 3.5 Hz, 1H), 2. 44 (s, 3H) ppm; ^{13}C-NMR (101 MHz, DMSO-*d$_6$*) δ 167.84, 167.40, 154.73, 153.67, 147.62, 147.21, 137.90, 134.74, 133.04, 131.62, 130.09, 129.95, 129.51, 129.30, 127.08, 125.06, 123.02, 118.41, 112.06, 108.90, 22.05. UHPLC-MS (ESI, m/z) R_t = 3.79 min, 360 $[M+H]^+$

Sodium 6-methyl-2-(5-phenylfuran-2-yl)quinoline-4-carboxylate (**4**) (Scheme 1A) Compound **4** was obtained via general procedure E using intermediate **41** (158 mg, 0.46 mmol) and NaOH 30% (1.23 ml, 9.20 mmol). After filtration, **4** was obtained as pure compound (151 mg, quantitative yield). ^{1}H- NMR (401 MHz, DMSO-*d$_6$*) δ 8.39 (dt, J = 1.9, 0.8 Hz, 1H), 7.91 (s, 1H), 7.86 - 7.83 (m, 2H), 7.81 (d, J = 8. 6 Hz, 1H), 7.50 - 7.42 (m, 3H), 7.35 - 7.29 (m, 2H), 7.15 (d, J = 3.6 Hz, 1H), 2.44 (d, J = 1.0 Hz, 3H) ppm. ^{13}C-NMR (101 MHz,

DMSO-*d_6*) δ 166.64, 166.36, 154.08, 152.89, 147.40, 147.21, 138.21, 136.08, 133.21, 130.97, 130.59, 130.11, 129.63, 129.10, 129.03, 124.56, 124.43, 123.44, 118.46, 113.87, 110.39, 22.02 ppm. UHPLC-MS (ESI, m/z) R_t = 3.27 min, 330 $[M+H]^+$. ESI-HRMS calculated for $C_{21}H_{16}NO_3$ $[M+H]^+$ 330.1125, found 330.1122. ESI-HRMS calculated for $C_{21}H_{15}NNaO_3$ $[M+H]^+$ 352.0944, found 352.0955.

Sodium 2-(5-(2-methoxyphenyl)furan-2-yl)-6-methylquinoline-4-carboxylate (**5**) (Scheme 1A) Compound **5** was obtained via general procedure E using intermediate **42** (132 mg, 0.353 mmol) and NaOH 30% (0.942 ml, 7.07 mmol). After filtration, **5** was obtained as pure compound (134 mg, quantitative yield). ^{1}H-NMR (401 MHz, CD_3OD-*d_4*) δ 8.17 (dd, J = 7.9, 1.8 Hz, 2H), 8.04 (s, 1H), 7.98 (d, J = 8.7 Hz, 1H), 7.59 (dd, J = 8.7, 2.0 Hz, 1H), 7.39 (dd, J = 3.6, 0.5 Hz, 1H), 7.33 (ddd, J = 8.6, 7.2, 1.7 Hz, 1H), 7.15 (d, J = 3.6 Hz, 1H), 7.13 – 7.07 (m, 2H), 4.00 (s, 3H), 2.54 (s, 3H). ^{13}C-NMR (101 MHz, CD_3OD-*d_4*) δ 174.97, 157.47, 153.55, 152.62, 149.34, 149.30, 148.08, 137.52, 133.28, 130.09, 128.66, 127.34, 126.77, 125.27, 121.89, 120.40, 116.00, 113.79, 113.73, 112.32, 55.92, 21.78. UHPLC-MS (ESI, m/z) R_t = 3.32 min, 360 $[M+H]^+$

2-(5-(2-Hydroxyphenyl)furan-2-yl)-6-methylquinoline-4-carboxylic acid (6) (Scheme 1A) Compound **6** was obtained via general procedure E using intermediate **43** (158 mg, 0.44 mmol) and NaOH 30% (1.17 ml, 8.79 mmol). After acidic precipitation and Et_2O washing, **6** was obtained as pure compound (151 mg, quantitative yield). ^{1}H-NMR (401 MHz, DMSO-*d_6*) δ 10.51 (s, 1H), 8.38 (s, 1H), 8.30 (s, 1H), 7.98 (d, J = 8.6 Hz, 1H), 7.90 (dd, J = 7.9, 1.7 Hz, 1H), 7.66 (dd, J = 8.6, 1.9 Hz, 1H), 7.47 (d, J = 3.5 Hz, 1H), 7.23 – 7.14 (m, 2H), 7.08 (d, J = 8.1 Hz, 1H), 6.97 (t, J = 7.5 Hz, 1H), 2.52 (s, 3H). ^{13}C-NMR (101 MHz, DMSO-*d_6*) δ 167.69, 154.21, 152.16, 150.71, 147.19, 147.08, 136.90, 132.40, 128.98, 128.95, 125.42, 124.46, 123.31, 119.34, 117.54, 116.72, 116.30, 112.98, 112.20, 21.59. UHPLC-MS (ESI, m/z) R_t = 2.74 min, 346 $[M+H]^+$

Sodium 6-methyl-2-(5-(o-tolyl)furan-2-yl)quinoline-4-carboxylate (7) (Scheme 1A) Compound **7** was obtained via general procedure E using intermediate **44** (115 mg, 0.322 mmol) and NaOH 30% (0.858 ml, 6.44 mmol). After filtration, **7** was obtained as pure compound (117 mg, quantitative yield). ^{1}H-NMR (401 MHz, CD_3OD-*d_4*) δ 8.15 (s, 1H), 8.03 (s, 1H), 8.00 – 7.90 (m, 2H), 7.59 (dd, J = 8.7, 2.0 Hz, 1H), 7.43 (d, J = 3.6 Hz, 1H), 7.36 – 7.22 (m, 3H), 6.87 (d, J = 3.6 Hz, 1H), 2.60 (s, 3H), 2.54 (s, 3H). ^{13}C-NMR (101 MHz, CD_3OD-*d_4*) δ 173.53, 155.12, 152.13, 147.99, 147.83, 146.66, 136.16, 134.68, 131.87, 130.90, 129.44, 127.78, 127.32, 126.96, 125.82, 125.31, 114.38, 111.75, 110.97, 20.77, 17.67. UHPLC-MS (ESI, m/z) R_t = 3.49 min, 344 $[M+H]^+$

Sodium 2-(5-(2-fluorophenyl)furan-2-yl)-6-methylquinoline-4-carboxylate (8) (Scheme 1A) Compound **8** was obtained via general procedure E using intermediate **45** (80 mg, 0.221 mmol) and NaOH 30% (0.59 mL, 4.43 mmol). After filtration, **8** was obtained as pure compound (81 mg, quantitative yield). ^{1}H-NMR (600 MHz, DMSO-*d_6*) δ 8.43 (s, 1H), 8.03 (td, J = 8.0, 2.0 Hz, 1H), 7.98 (s, 1H), 7.86 (d, J = 8. 5 Hz, 1H), 7.52 (dd, J = 8.6, 2.1 Hz, 1H), 7.46 - 7.34 (m, 4H), 7.07 (t, J = 3.5 Hz, 1H), 2.48 (s, 3H) ppm. ^{13}C-NMR (101 MHz, DMSO-*d_6*) δ 169.38, 158.93, 157.27, 153.47, 149.68, 147.99, 146.93, 134.68, 131.35, 129.61, 128.33, 126.64, 126.10, 125.22, 124.73, 117.87, 116.40, 114.71, 112.81, 111.60, 21.46 ppm. UHPLC-MS (ESI, m/z) R_t = 3.47

min, 348 [M+H]$^+$ In agreement with Horak, Y. *et al.*[2] ESI-HRMS calculated for $C_{21}H_{15}FNO_3$ [M+H]$^+$ 348.1030, found 348.1036. ESI-HRMS calculated for $C_{21}H_{14}FNNaO_3$ [M+H]$^+$ 370.0850, found 370.856. ESI-HRMS calculated for $C_{21}H_{13}FNO_3$ [M-H]$^-$ 346.0885, found 346.0888. ESI-HRMS calculated for $C_{21}H_{13}$ $ClFNNaO_3$ [M-H+ Cl]$^-$ 404.0471, found 404.0474.

6-Methyl-2-(5-(pyridin-4-yl)furan-2-yl)quinoline-4-carboxylic acid (9) (Scheme 1A) Compound **9** was obtained via general procedure E using intermediate **46** (130 mg, 0.377 mmol) and NaOH 30% (1 mL, 7.55 mmol). After acidic precipitation, **9** was obtained as pure compound (124 mg, quantitative yield). ^{1}H-NMR (401 MHz, DMSO-*d$_6$*) δ 8.71 – 8.65 (m, 2H), 8.40 (d, J = 3.1 Hz, 2H), 8.03 (d, J = 8.6 Hz, 1H), 7.90 – 7.84 (m, 2H), 7.70 (dd, J = 8.7, 2.0 Hz, 1H), 7.61 – 7.53 (m, 2H), 2.55 (s, 3H) ppm. ^{13}C-NMR (101 MHz, DMSO-*d$_6$*) δ 167.89, 154.09, 152.44, 150.73, 147.46, 147.46, 147.10, 138.19, 137.58, 136.68, 133.13, 129.63, 124.73, 123.95, 123.95, 118.33, 113.52, 113.11, 22.08 ppm. UHPLC-MS (ESI, m/z) R_t = 1.49 min, 331 [M+H]$^+$

6-Methyl-2-(5-(pyridin-3-yl)furan-2-yl)quinoline-4-carboxylic acid (10) (Scheme 1A) Compound **10** was obtained via general procedure E using intermediate **47** (88 mg, 0.255 mmol) and NaOH 30% (0.681 mL, 5.11 mmol). After acidic precipitation, **10** was obtained as pure compound (84 mg, quantitative yield). ^{1}H-NMR (401 MHz, DMSO-*d$_6$*) δ 9.23 (d, J = 2.3 Hz, 1H), 8.63 (dd, J = 5.0, 1.6 Hz, 1H), 8.42 (d, J = 4.6 Hz, 3H), 8. 02 (d, J = 8.6 Hz, 1H), 7.68 (m, 2H), 7.57 (d, J = 3.6 Hz, 1H), 7.46 (d, J = 3.6 Hz, 1H), 2.55 (s, 3H). ^{13}C-NMR (101 MHz, DMSO-*d$_6$*) δ 167.89, 154.09, 152.44, 150.73, 148.95, 147.46, 147.10, 138.19, 137.58, 136.68, 134.11, 133.41, 133.13, 129.63, 124.73, 123.95, 118.33, 113.52, 113.11, 22.08. UHPLC-MS (ESI, m/z) R_t = 2.28 min, 331 [M+H]$^+$

2-(5-(2-Carboxyphenyl)furan-2-yl)quinoline-4-carboxylic acid (11) (Scheme 1A) Compound **11** was obtained via general procedure E using intermediate **48** (90 mg, 0.232 mmol) and NaOH 30% (0.619 ml, 4.65 mmol). After acidic precipitation, **11** was obtained as pure compound (83 mg, quantitative yield). ^{1}H-NMR (401 MHz, DMSO-*d$_6$*) δ 8.57 (dd, J = 8.6, 1.5 Hz, 1H), 8.26 (s, 1H), 8.11 - 8.02 (m, 1H), 7.81 (ddd, J = 8. 3, 6.9, 1.4 Hz, 2H), 7.68 - 7.64 (m, 2H), 7.63 - 7.57 (m, 1H), 7.54 - 7.45 (m, 2H), 6.99 (d, J = 3.6 Hz, 1H) ppm. ^{13}C-NMR (101 MHz, DMSO-*d$_6$*) δ 170.24, 167.77, 154.41, 152.84, 148.68, 148.26, 138.23, 132.22, 131.35, 131.17, 130.98, 129.60, 129.15, 129.07, 128.56, 128.23, 128.08, 125.98, 123.74, 118.03, 113.55, 111.54 ppm. UHPLC-MS (ESI, m/z) R_t = 2.58 min, 360 [M+H]$^+$ In agreement with Horak, Y. *et al.*[2] ESI-HRMS calculated for $C_{21}H_{14}NO_5$ [M+H]$^+$ 360.0866, found 360.0861.

Sodium 2-(5-phenylfuran-2-yl)quinoline-4-carboxylate (12) (Scheme 1A) Compound **12** was obtained via general procedure E using intermediate **49** (70 mg, 0.212 mmol) and NaOH 30% (0.57 ml, 4.25 mmol). After filtration, **12** was obtained as pure compound (71 mg, quantitative yield). ^{1}H-NMR (401 MHz, DMSO-*d$_6$*) δ 8.67 (dd, J = 8.4, 1.5 Hz, 1H), 8.00 (s, 1H), 7.97 - 7.93 (m, 1H), 7.89 (dd, J = 8.3, 1.2 Hz, 2H), 7. 67 (ddd, J = 8.4, 6.8, 1.5 Hz, 1H), 7.55 - 7.43 (m, 3H), 7.40 (d, J = 3.5 Hz, 1H), 7.39 - 7.31 (m, 1H), 7.20 (d, J = 3.6 Hz, 1H) ppm. ^{13}C NMR (151 MHz, DMSO-*d$_6$*) δ 154.08, 153.20, 148.23, 147.94, 129.90, 129.15, 129.07, 128.45,

128.03, 127.86, 125.22, 124.79, 123.80, 114.68, 112.05, 108.65. UHPLC-MS (ESI, m/z) R_t = 3.5 min, 316 $[M+H]^+$

Sodium 6-fluoro-2-(5-phenylfuran-2-yl)quinoline-4-carboxylate (**13**) (Scheme 2A) Compound **13** was obtained via general procedure E using intermediate **69** (105 mg, 0.30 mmol) and NaOH 30% (0.8 ml, 6 mmol). After filtration, **13** was obtained as pure compound (107 mg, quantitative yield). ^{1}H-NMR (401 MHz, DMSO-d_6) δ 8.62 (dd, J = 11.4, 3.0 Hz, 1H), 8.11 (s, 1H), 7.97 (dd, J = 9.3, 5.8 Hz, 1H), 7.89 - 7. 81 (m, 2H), 7.54 (ddd, J = 9.2, 8.1, 3.1 Hz, 1H), 7.47 (t, J = 7.7 Hz, 2H), 7.39 - 7.31 (m, 2H), 7.17 (d, J = 3.5 Hz, 1H). ^{13}C-NMR (101 MHz, DMSO-d_6) δ 168.26, 160.78, 158.38, 154.56, 153.36, 149.30, 149.24, 148.00, 147.98, 146.00, 131.50, 131.41, 130.27, 129.48, 128.47, 126.36, 126.25, 124.23, 119.60, 119.34, 116.79, 112.51, 111.72, 111.49, 109.07, 40.41, 31.11, 17.49. UHPLC-MS (ESI, m/z) R_t = 3.37 min, 334 $[M+H]^+$

Sodium 2-(5-phenylfuran-2-yl)-6-(trifluoromethyl)quinoline-4-carboxylate (**14**) (Scheme 2A) Compound **14** was obtained via general procedure E using intermediate **70** (60 mg, 0.151 mmol) and NaOH 30% (0.4 ml, 3 mmol). After filtration, **14** was obtained as pure compound (61 mg, quantitative yield). ^{1}H-NMR (401 MHz, $CDCl_3$-*d*) δ 8.90 - 8.84 (m, 1H), 8.20 (d, J = 12.9 Hz, 2H), 7.91 (td, J = 8.9, 1. 8 Hz, 3H), 7.50 (d, J = 3.7 Hz, 1H), 7.45 (dd, J = 8.4, 7.0 Hz, 2H), 7.38 - 7.31 (m, 1H), 7.03 (d, J = 3.6 Hz, 1H). ^{13}C-NMR (101 MHz, $CDCl_3$-*d*) δ 172.01, 156.36, 151.93, 150.55, 149.36, 148.56, 129.97, 129.11, 128.53, 128.02, 127.74, 127.35, 127.02, 125.94, 125.64, 125.00, 124.96, 124.79, 124.74, 123.99, 123.21, 122.95, 116.18, 113.96, 107.73, 17.84, 17.80. UHPLC-MS (ESI, m/z) R_t = 3.87 min, 384 $[M+H]^+$

6-Bromo-2-(5-phenylfuran-2-yl)quinoline-4-carboxylic acid (**15**) (Scheme 1B) Compound **15** was synthesized via general procedure A using 5-bromoindolin 2,3-dione **50** (105 mg, 0.464 mmol), 5-phenyl-2-acetyl furan **52** (103.8 mg, 0.557 mmol) and KOH (78.19 mg, 1.39 mmol). After work-up, **15** was obtained as pure compound, without further purification (68 mg, yield 37%). ^{1}H-NMR (600 MHz, DMSO-d_6) δ 8.90 (d, J = 2.2 Hz, 1H), 8.44 (s, 1H), 8.02 (d, J = 9.0 Hz, 1H), 7.94 (dd, J = 9.0, 2.3 Hz, 1H), 7.92 – 7.88 (m, 2H), 7.58 (d, J = 3.6 Hz, 1H), 7.51 (t, J = 7.8 Hz, 2H), 7.42 – 7.36 (m, 1H), 7.25 (d, J = 3.6 Hz, 1H) ppm. ^{13}C-NMR (151 MHz, DMSO-d_6) δ 167.31, 155.76, 152.10, 148.78, 147.64, 136.37, 133.91, 131.86, 129.98, 129.56, 129.56, 128.93, 128.17, 125.03, 124.56, 124.56, 121.32, 119.76, 114.71, 109.51 ppm. UHPLC-MS (ESI, m/z) R_t = 3.63 min, 394-396 $[M+H]^+$ ESI-HRMS calculated for $C_{20}H_{13}BrNO_3$ $[M+H]^+$ 394.0073, found 394.0083. ESI-HRMS calculated for $C_{20}H_{11}BrNO_3$ $[M-H]^-$ 391.9928, found 391.9929.

Sodium 6-ethyl-2-(5-phenylfuran-2-yl)quinoline-4-carboxylate (**16**) (Scheme 2A) Compound **16** was obtained as general procedure E using intermediate **71** (117 mg, 0.327 mmol) and NaOH 30% (0.873 ml, 6.55 mmol). After filtration, **16** was obtained as pure compound (119 mg, quantitative yield). ^{1}H-NMR (401 MHz, $CDCl_3$-*d*) δ 8.18 - 8.15 (m, 1H), 8.02 (s, 1H), 7.99 (d, J = 8.7 Hz, 1H), 7.93 - 7.88 (m, 2H), 7.62 (dd, J = 8.7, 2.0 Hz, 1H), 7. 44 (dd, J = 8.4, 7.1 Hz, 2H), 7.38 (d, J = 3.6 Hz, 1H), 7.35 - 7.28 (m, 1H), 6.99 (d, J = 3.6 Hz, 1H), 2.83 (q, J = 7.6 Hz, 2H), 1.33 (t, J = 7.6 Hz, 3H). ^{13}C-NMR (101 MHz, $CDCl_3$-*d*) δ 173.51, 155.52, 152.35, 148.06, 147.74, 146.82, 142.44, 130.85, 130.20, 128.47, 127.71, 127. 40, 124.10, 123.89, 123.82,

114.41, 112.28, 107.44, 67.41, 48.18, 47.97, 47.76, 47.55, 47.34, 47.12, 46.91, 28.62, 25.04, 17.77, 14.43. UHPLC-MS (ESI, m/z) R_t = 3.46 min, 344 $[M+H]^+$ ESI-HRMS calculated for $C_{22}H_{18}NO_3$ $[M+H]^+$ 344.1281, found 344.1284.

6-Amino-2-(5-phenylfuran-2-yl)quinoline-4-carboxylic acid (**17**) (Scheme 1B) In a microwavable vessel, **53** (80 mg, 0.22 mmol, 1 eq) was solubilized in anhydrous methanol (0.2 M) and KOH (150 mg, 2.66 mmol, 12 eq) was added. The reaction was microwaved at 90 °C for 1.5 hours. Next, 1 mL of water was added, and methanol was removed under reduced pressure. The basic water was acidified by adding conc HCl dropwise. At neutral pH, **17** precipitated as a solid, recovered by filtration and dried by vacuum to afford the pure compound (18 mg, yield 25%). ^{1}H-NMR (600 MHz, DMSO-d_6) δ 8.14 (s, 1H), 7.87 - 7.83 (dd, J = 9.0, 2.5 Hz, 2H), 7.76 (d, J = 9.0 Hz, 1H), 7.65 (d, J = 2.5 Hz, 1H), 7. 51 - 7.44 (t, J = 9.0 Hz, 2H), 7.37 - 7.31 (m, 1H), 7.26 (d, J = 3.5 Hz, 1H), 7.22 (dd, J = 9.0, 2.5 Hz, 1H), 7.16 (d, J = 3.5 Hz, 1H) ppm. ^{13}C-NMR (151 MHz, DMSO-d_6) δ 169.02, 153.92, 153.50, 148.66, 143.31, 142.83, 130.62, 130.43, 129.50, 128.29, 126.59, 124.11, 122.68, 117.88, 110.90, 109.06, 103.77 ppm. UHPLC-MS (ESI, m/z) R_t = 2.63 min, 331 $[M+H]^+$. ESI-HRMS calculated for $C_{20}H_{15}N_2O_3$ $[M+H]^+$ 331.1077, found 331.1079. ESI-HRMS calculated for $C_{20}H_{13}N_2O_3$ $[M-H]^-$ 329.0932, found 329.0930.

6-Hydroxy-2-(5-phenylfuran-2-yl)quinoline-4-carboxylic acid (**18**) (Scheme 2B) Compound **18** was obtained via general procedure H. 4-Aminophenol **73** (150 mg, 1.37 mmol), 5-phenylfurfural **74** (260.34 mg, 1.51 mmol, 1.1 equiv), complexed boron-diethyl ether trifluoride **59** (0.062 mL, 0.687 mmol) and 0.1 mL (0.1% mol) of acetic acid were added, and the reaction was stirred at 65°C for 3 hours. After 3 hours, pyruvic acid **60** (0.07 mL, 0.995 mmol) was added. **18** was obtained as pure compound (22 mg, yield 7%). ^{1}H-NMR (600 MHz, DMSO-d_6) δ 8.33 (s, 1H), 8.02 (d, J = 2.7 Hz, 1H), 7.97 (d, J = 9.1 Hz, 1H), 7.92 – 7.86 (m, 2H), 7.50 (t, J = 7.8 Hz, 2H), 7.44 – 7.34 (m, 3H), 7.20 (d, J = 3.5 Hz, 1H) ppm. ^{13}C-NMR (151 MHz, DMSO-d_6) δ 168.12, 157.32, 154.66, 152.82, 145.14, 144.38, 131.47, 130.25, 129.53, 128.57, 125.59,124.31, 124.31, 123.34, 118.65, 112.39, 109.20, 107.30 ppm. UHPLC-MS (ESI, m/z) R_t = 2.85 min, 332 $[M+H]^+$ ESI-HRMS calculated for $C_{20}H_{14}NO_4$ $[M+H]^+$ 332.0917 found 332.0920.

Sodium 6-methoxy-2-(5-phenylfuran-2-yl)quinoline-4-carboxylate (**19**) (Scheme 2A) Compound **19** was obtained via general procedure E using intermediate **72** (115 mg, 0.32 mmol) and NaOH 30% (0.853 ml, 6.40 mmol). After filtration, **19** was obtained as pure compound (117 mg, quantitative yield). ^{1}H-NMR (401 MHz, $CDCl_3$-*d*) δ 8.07 (s, 1H), 7.96 (d, J = 9.2 Hz, 1H), 7.92 - 7.87 (m, 2H), 7.83 (d, J = 2.9 Hz, 1H), 7. 43 (t, J = 7.7 Hz, 2H), 7.37 (dd, J = 9.3, 2.8 Hz, 1H), 7.34 - 7.27 (m, 2H), 6.97 (d, J = 3.6 Hz, 1H), 3.93 (s, 3H). ^{13}C-NMR (101 MHz, $CDCl_3$-*d*) δ 173.41, 157.88, 155.23, 152.44, 146.71, 146.24, 144.16, 130.28, 128.92, 128.45, 127.60, 125.16, 123.75, 122.40, 115.29, 111.60, 107.37, 104.30, 56.88, 54.59, 18.44, 18.41, 18.29, 16.92. UHPLC-MS (ESI, m/z) Rt = 3.52 min, 346 $[M+H]^+$. ESI-HRMS calculated for $C_{21}H_{16}NO_4$ $[M+H]^+$ 346.1074, found 346.1072. ESI-HRMS calculated for $C_{21}H_{14}NNaO_4$ $[M+H]^+$ 368.0893, found 368.0895.

6-Methyl-2-(5-phenylthiophen-2-yl)quinoline-4-carboxylic acid (20) (Scheme 2C) Compound **20** was obtained via general procedure D using the intermediate **77** (105 mg, 0.301 mmol), boronic acid **31** (55 mg, 0.452 mmol), K_2CO_3 (125 mg, 0.904 mmol) and palladium tetrakis (17 mg, 15.08 mmol). After removing the dioxane under vacuum, the basic water was acidified to pH 3 to form a precipitate, recovered by filtration. The crude product was purified over silica gel flash chromatography (0-10% AcOEt/EP) to afford **20** as pure compound (79 mg, yield 76%). ^{1}H-NMR (401 MHz, DMSO-*d$_6$*) δ 8.40 – 8.35 (m, 1H), 7.94 (s, 1H), 7.90 (d, J = 3.9 Hz, 1H), 7.83 – 7.74 (m, 3H), 7.58 (d, J = 3.9 Hz, 1H), 7.53 – 7.47 (dd, J = 7.9, 1.9 Hz, 1H), 7.53 – 7.47 (t, J = 7.9 Hz, 2H), 7.39 – 7.30 (m, 1H), 2.49 – 2.44 (m, 3H) ppm. ^{13}C-NMR (151 MHz, DMSO-*d$_6$*) δ 169.76, 150.92, 150.21, 146.99, 145.76, 145.21, 134.87, 134.17, 131.73, 129.66, 129.66, 128.45, 128.45, 127.53, 127.09, 125.89, 125.89, 125.35, 125.22, 114.90, 21.89 ppm. UHPLC-MS (ESI, m/z) R_t = 3.47 min, 346 $[M+H]^+$ ESI-HRMS calculated for $C_{21}H_{16}NO_2S$ $[M+H]^+$ 346.0896, found 346.0899.

2-(Furan-2-yl)-6-methylquinoline-4-carboxylic acid (24) (Scheme 1A) Compound **24** was synthesized via general procedure A using 5-methylisatin **21** (2 g, 12.41 mmol), 2-acetylfuran **23** (1.64 g, 14.9 mmol) and KOH (2.1 g, 37.23 mmol). After work-up, **24** was obtained as pure compound (2.88 g, yield 75%). ^{1}H-NMR (401 MHz, $CDCl_3$-*d*) δ 8.44 (d, J = 1.8 Hz, 1H), 8.25 (s, 1H), 7.98 (d, J = 8.7 Hz, 1H), 7.77 (d, J = 1. 7 Hz, 1H), 7.63 (dd, J = 8.7, 2.0 Hz, 1H), 7.34 (d, J = 3.5 Hz, 1H), 6.67 (dd, J = 3.5, 1.8 Hz, 1H), 2.54 (s, 3H) ppm. UHPLC-MS (ESI, m/z) R_t = 2.05 min, 254 $[M+H]^+$

2-(Furan-2-yl)quinoline-4-carboxylic acid (25) (Scheme 1A) Compound **25** was synthesized via general procedure A using isatin **22** (1 g, 6.8 mmol), 2-acetylfuran **23** (0.898 g, 8.16 mmol) and KOH (1.14 g, 20.39 mmol). After work-up, **25** was obtained as pure compound (0.876 g, yield 54%). ^{1}H-NMR (401 MHz, $CDCl_3$-*d*) δ 8.44 (dd, J = 8.7, 1.8 Hz, 1H), 8.25 (s, 1H), 7.98 (dd, J = 8.7, 1.8 Hz, 1H), 7.79 (t, J = 8. 7 Hz, 1H), 7.77 (dd, J = 8.7, 1.7 Hz, 1H), 7.63 (t, J = 8.7 Hz, 1H), 7.34 (d, J = 3.5 Hz, 1H), 6.67 (dd, J = 3.5, 1.8 Hz, 1H) ppm. UHPLC-MS (ESI, m/z) R_t = 2.7 min, 240 $[M+H]^+$

Methyl 2-(furan-2-yl)-6-methylquinoline-4-carboxylate (26b) (Scheme 1A) Compound **26b** was obtained via general procedure B using quinoline **24** (1 g, 3.95 mmol) and $SOCl_2$ (0.864 mL, 11.85 mmol). **26b** was obtained as pure compound (683 mg, yield 65%). ^{1}H-NMR (401 MHz, $CDCl_3$-*d*) δ 8.44 (d, J = 1.8 Hz, 1H), 8.25 (s, 1H), 7.98 (d, J = 8.7 Hz, 1H), 7.77 (d, J = 1.7 Hz, 1H), 7. 63 (dd, J = 8.7, 2.0 Hz, 1H), 7.34 (d, J = 3.5 Hz, 1H), 6.67 (dd, J = 3.5, 1.8 Hz, 1H), 3.90 (s, 3H), 2.54 (s, 3H) ppm. UHPLC-MS (ESI, m/z) Rt = 3.35 min, 268 $[M+H]^+$

Methyl 2-(5-bromofuran-2-yl)-6-methylquinoline-4-carboxylate (26c) (Scheme 1A) Compound **26c** was obtained via general procedure C using intermediate ester **26b** (570 mg, 2.13 mmol) and *N*-bromosuccinimide (569 mg, 3.2 mmol). The crude was purified over silica gel flash chromatography (EP:EtOAc 95:5) to afford **26c** as pure compound (567 mg, yield 77%). ^{1}H-NMR (401 MHz, $CDCl_3$-*d*) δ 8.44 (d, J = 1.8 Hz, 1H), 8.25 (s, 1H), 7.98 (d, J = 8.7 Hz, 1H), 7.63 (dd, J = 8.7, 2.0 Hz, 1H), 7.34 (d, J = 3.5 Hz, 1H), 6.67 (d, J = 3.5, 1H), 2.54 (s, 3H) ppm. UHPLC-MS (ESI, m/z) R_t = 3.7 min, 346-348 $[M+H]^+$

Methyl 2-(furan-2-yl)quinoline-4-carboxylate (27b) (Scheme 1A) Compound **27b** was obtained via general procedure B using quinoline **25** (0.876 g, 3.66 mmol) and $SOCl_2$ (0.801 mL, 10.99 mmol). **27b** was obtained as pure compound (45 mg, yield 80%). ^{1}H-NMR (401 MHz, $CDCl_3$-*d*) δ 8.44 (dd, J = 8.7, 1.8 Hz, 1H), 8.25 (s, 1H), 7.98 (dd, J = 8.7, 1.8 Hz, 1H), 7.79 (t, J = 8.7 Hz, 1H), 7. 77 (dd, J = 8.7, 1.7 Hz, 1H), 7.63 (t, J = 8.7 Hz, 1H), 7.34 (d, J = 3.5 Hz, 1H), 6.67 (dd, J = 3.5, 1.8 Hz, 1H), 3.90 (s, 3H) ppm. UHPLC-MS (ESI, m/z) R_t = 3.23 min, 254 $[M+H]^+$

Methyl 2-(5-bromofuran-2-yl)quinoline-4-carboxylate (27c) (Scheme 1A) Compound **27c** was obtained via general procedure C using intermediate ester **27b** (750 mg, 2.94 mmol) and *N*-bromosuccinimide (785.36 mg, 4.41 mmol). The crude was purified over silica gel flash chromatography (EP:EtOAc 95:5) to afford **27c** as pure compound (652 mg, yield 67%). ^{1}H-NMR (401 MHz, $CDCl_3$-*d*) δ 8.66 - 8.54 (m, 1H), 8.15 (s, 1H), 8.03 (dd, J = 8.6, 1.3 Hz, 1H), 7.65 (ddd, J = 8.3, 6. 8, 1.4 Hz, 1H), 7.50 (ddd, J = 8.3, 6.8, 1.3 Hz, 1H), 7.17 (d, J = 3.5 Hz, 1H), 6.46 (d, J = 3.5 Hz, 1H), 3.98 (s, 3H) ppm. UHPLC-MS (ESI, m/z) R_t = 3.6 min, 332-334 $[M+H]^+$

Methyl 2-(5-(2-(methoxycarbonyl)phenyl)furan-2-yl)-6-methylquinoline-4-carboxylate (38) (Scheme 1A) Compound **38** was obtained via general procedure D using brominated intermediate **26c** (230 mg, 0.664 mmol), boronic acid **28** (179 mg, 0.997 mmol), K_2CO_3 (276 mg, 2 mmol) and palladium tetrakis (38 mg, 0.033 mmol). The crude was purified over silica gel flash chromatography (EP:EtOAc 95:5) to afford **38** as pure compound (124 mg, yield 46%). ^{1}H NMR (401 MHz, DMSO-d^6) δ 8.35 (dt, J = 1.9, 0.9 Hz, 1H), 8.23 (s, 1H), 8.00 (d, J = 8.6 Hz, 1H), 7.89 (dd, J = 8.2, 1.2 Hz, 1H), 7.73 – 7.63 (m, 3H), 7.54 – 7.48 (m, 2H), 7.13 (d, J = 3.6 Hz, 1H), 4.03 (s, 3H), 3.90 (s, 3H), 2.54 (s, 3H) ppm. UHPLC-MS (ESI, m/z) Rt = 3.71 min, 402 $[M+H]^+$

Methyl 2-(5-(3-(methoxycarbonyl)phenyl)furan-2-yl)-6-methylquinoline-4-carboxylate (39) (Scheme 1A) Compound **39** was obtained via general procedure D using brominated intermediate **26c** (230 mg, 0.664 mmol), boronic acid **29** (179 mg, 0.997 mmol), K_2CO_3 (276 mg, 2 mmol) and palladium tetrakis (38 mg, 0.033 mmol). The crude was purified over silica gel flash chromatography (EP:EtOAc 95:5) to afford **39** as pure compound (186 mg, yield 70%). ^{1}H-NMR (401 MHz, DMSO-d_6) δ 8.36 (td, J = 1.7, 0.5 Hz, 1H), 8.31 (s, 1H), 8.25 (p, J = 0.8 Hz, 1H), 8.14 (ddd, J = 7.8, 1.9, 1.2 Hz, 1H), 8.00 (d, J = 8.6 Hz, 1H), 7. 91 (ddd, J = 7.8, 1.7, 1.1 Hz, 1H), 7.69 - 7.61 (m, 2H), 7.51 (d, J = 3.6 Hz, 1H), 7.34 (d, J = 3.6 Hz, 1H), 4.02 (s, 3H), 3.90 (s, 3H), 2.52 - 2.49 (s, 3H) ppm. UHPLC-MS (ESI, m/z) R_t = 3.87 min, 402 $[M+H]^+$

Methyl 2-(5-(4-(methoxycarbonyl)phenyl)furan-2-yl)-6-methylquinoline-4-carboxylate (40) (Scheme 1A) Compound **40** was obtained via general procedure D using brominated intermediate **26c** (200 mg, 0.578 mmol), boronic acid **30** (156 mg, 0.867 mmol), K_2CO_3 (240 mg, 1.73 mmol) and palladium tetrakis (38 mg, 0.033 mmol). The crude was purified over silica gel flash chromatography (EP:AcOEt 9:1) to afford **40** as pure compound (207 mg, yield 89%). ^{1}H-NMR (401 MHz, $CDCl_3$-*d*) δ 8.47 (t, J = 1.5 Hz, 1H), 8.39 (s, 1H), 8.12 (dd, J = 8.5, 1.9 Hz, 3H), 7.93 - 7.85 (m, 2H), 7. 61 (dd, J = 8.7, 1.9 Hz, 1H), 7.48 (s, 1H), 7.00 (d, J = 3.5 Hz, 1H), 4.11 (s, 3H), 3.95 (s, 3H), 2.58 (s, 3H) ppm. UHPLC-MS (ESI, m/z) R_t = 3.85 min, 402 $[M+H]^+$

Methyl 6-methyl-2-(5-phenylfuran-2-yl)quinoline-4-carboxylate (41) (Scheme 1A) Compound **41** was obtained via general procedure D using brominated intermediate **26c** (210 mg, 0.606 mmol), boronic acid **31** (111 mg, 0.909 mmol), K_2CO_3 (251 mg, 1.82 mmol) and palladium tetrakis (35 mg, 0.03 mmol). The crude was purified over silica gel flash chromatography (EP:AcOEt 95:5) to afford **41** as pure compound (178 mg, yield 86%). ^{1}H-NMR (401 MHz, DMSO-*d*$_6$) δ 8.30 (s, 1H), 8.24 (s, 1H), 7.97 (d, J = 8.6 Hz, 1H), 7.91 - 7.82 (m, 2H), 7.65 (dd, J = 8. 7, 1.9 Hz, 1H), 7.52 - 7.44 (m, 3H), 7.40 - 7.33 (m, 1H), 7.20 (d, J = 3.6 Hz, 1H), 4.02 (s, 3H), 2.50 (s, 3H) ppm. UHPLC-MS (ESI, m/z) R_t = 3.88 min, 344 $[M+H]^+$

Methyl 2-(5-(2-methoxyphenyl)furan-2-yl)-6-methylquinoline-4-carboxylate (42) (Scheme 1A) Compound **43** was obtained via general procedure D using brominated intermediate **26c** (200 mg, 0.577 mmol), boronic acid **32** (118 mg, 0.867 mmol), K_2CO_3 (239 mg, 1.73 mmol, 2 M in water) and palladium tetrakis (33 mg, 0.029 mmol). The crude was purified over silica gel flash chromatography (DCM 100%) to afford **43** as pure compound (175 mg, yield 85%). ^{1}H-NMR (401 MHz, DMSO-*d*$_6$) δ 8.32 (s, 1H), 8.29 (s, 1H), 8.01 (d, J = 8.6 Hz, 1H), 7.87 (dd, J = 7.2, 2.2 Hz, 1H), 7.69 (dd, J = 8.7, 2.0 Hz, 1H), 7.53 (d, J = 3.6 Hz, 1H), 7.41 – 7.28 (m, 3H), 7.02 (d, J = 3.6 Hz, 1H), 4.03 (s, 3H), 2.57 (s, 3H), 2.54 (s, 3H). UHPLC-MS (ESI, m/z) R_t = 4.05 min, 358 $[M+H]^+$

Methyl 2-(5-(2-hydroxyphenyl)furan-2-yl)-6-methylquinoline-4-carboxylate (43) (Scheme 1A) Compound **43** was obtained via general procedure D using the brominated intermediate **26c** (200 mg, 0.577 mmol), boronic acid **33** (132 mg, 0.867 mmol), K_2CO_3 (239 mg, 1.73 mmol, 2 M in water) and palladium tetrakis (33 mg, 0.029 mmol). The crude was purified over silica gel flash chromatography (DCM 100%) to afford **43** as pure compound (186 mg, yield 86%). ^{1}H-NMR (401 MHz, DMSO-*d*$_6$) δ 8.36 (s, 1H), 8.27 (s, 1H), 8.05 – 7.98 (m, 2H), 7.74 – 7.67 (m, 1H), 7.51 (d, J = 3.6 Hz, 1H), 7.43 – 7.34 (m, 1H), 7.22 – 7.10 (m, 3H), 4.04 (s, 3H), 3.97 (s, 3H), 2.55 (s, 3H). UHPLC-MS (ESI, m/z) R_t = 3.91 min, 374 $[M+H]^+$

Methyl 6-methyl-2-(5-(o-tolyl)furan-2-yl)quinoline-4-carboxylate (44) (Scheme 1A) Compound **44** was obtained via general procedure D using brominated intermediate **26c** (200 mg, 0.577 mmol), boronic acid **34** (120 mg, 0.867 mmol), K_2CO_3 (239 mg, 1.73 mmol, 2 M in water) and palladium tetrakis (33 mg, 0.029 mmol). The crude was purified over silica gel flash chromatography (10-20% AcOEt/EP) to afford **44** as pure compound (181 mg, yield 87%). ^{1}H-NMR (401 MHz, DMSO-*d*$_6$) δ 10.35 (s, 1H), 8.34 (s, 1H), 8.27 (s, 1H), 8.01 (d, J = 8.6 Hz, 1H), 7.93 (dd, J = 7.8, 1.7 Hz, 1H), 7.69 (dd, J = 8.7, 1.9 Hz, 1H), 7.49 (d, J = 3.5 Hz, 1H), 7.25 – 7.16 (m, 2H), 7.04 – 6.94 (m, 2H), 4.04 (s, 3H), 2.54 (s, 3H).

Methyl 2-(5-(2-fluorophenyl)furan-2-yl)-6-methylquinoline-4-carboxylate (45) (Scheme 1A) Compound **45** was obtained via general procedure D using brominated intermediate **26c** (135 mg, 0.389 mmol), boronic acid **35** (82 mg, 0.584 mmol), KF (67.97 mg, 1.17 mmol, 1.2 M in water) and palladium tetrakis (23 mg, 0.0195 mmol). The solution was left stirring at reflux for 4 h. The crude was purified over silica gel flash chromatography (EP: AcOEt 95:5 and DCM:EP 1:1) to afford **45** as pure compound (52 mg, yield 37%). ^{1}H-NMR (600 MHz, DMSO-*d*$_6$) δ 8.37 (s, 1H), 8.30 - 8.26 (m, 1H), 8.06 (td, J = 8.0, 2.0 Hz, 1H), 8.02 (d, J = 8.5

Hz, 1H), 7.70 (dd, J = 8. 6, 1.9 Hz, 1H), 7.55 (d, J = 3.6 Hz, 1H), 7.48 - 7.43 (m, 1H), 7.42 - 7.36 (m, 2H), 7.10 (t, J = 3.5 Hz, 1H), 4.04 (s, 3H), 2.54 (s, 3H) ppm. UHPLC-MS (ESI, m/z) R_t = 4.39 min, 362 $[M+H]^+$

Methyl 6-methyl-2-(5-(pyridin-4-yl)furan-2-yl)quinoline-4-carboxylate (46) (Scheme 1A) Compound **46** was obtained via general procedure D using brominated intermediate **26c** (181 mg, 0.523 mmol), boronic acid **36** (110 mg, 0.784 mmol), K_2CO_3 (217 mg, 1.57 mmol) and palladium tetrakis (30 mg, 0.026 mmol). The crude was purified by CombiFlash chromatographic (SiO_2 gold 12 g; DCM: MeOH, 9:1) to afford **46** as pure compound (130 mg, yield 72%). ^{1}H-NMR (401 MHz, DMSO-d_6) δ 8.71 - 8.65 (m, 2H), 8.40 (d, J = 3.1 Hz, 2H), 8.03 (d, J = 8.6 Hz, 1H), 7. 90 - 7.84 (m, 2H), 7.70 (dd, J = 8.7, 2.0 Hz, 1H), 7.61 - 7.53 (m, 2H), 4.02 (s, 3H), 2.55 (s, 3H) ppm. UHPLC-MS (ESI, m/z) R_t = 1.49 min, 331 $[M+H]^+$

Methyl 6-methyl-2-(5-(pyridin-3-yl)furan-2-yl)quinoline-4-carboxylate (47) (Scheme 1A) Compound **47** was obtained via general procedure D using brominated intermediate **26c** (150 mg, 0.433 mmol), boronic acid **37** (80 mg, 0.649 mmol), K_2CO_3 (179 mg, 1.30 mmol) and palladium tetrakis (25 mg, 0.022 mmol). The crude p was purified over silica gel flash chromatography (DCM: MeOH, 9:1) to afford **47** as pure compound (88 mg, yield 59%). ^{1}H-NMR (401 MHz, DMSO-d_6) δ 9.23 (d, J = 2.3 Hz, 1H), 8.63 (dd, J = 5.0, 1.6 Hz, 1H), 8.42 (d, J = 4.6 Hz, 3H), 8. 02 (d, J = 8.6 Hz, 1H), 7.68 (m, 2H), 7.57 (d, J = 3.6 Hz, 1H), 7.46 (d, J = 3.6 Hz, 1H), 4.02 (s, 3H), 2.54 (s, 3H) ppm. UHPLC-MS (ESI, m/z) R_t = 3.36 min, 345 $[M+H]^+$

Methyl 2-(5-(2-(methoxycarbonyl)phenyl)furan-2-yl)quinoline-4-carboxylate (48) (Scheme 1A) Compound **48** was obtained via general procedure D using brominated intermediate **27c** (200 mg, 0.602 mmol), boronic acid **28** (216 mg, 1.20 mmol), K_2CO_3 (250 mg, 1.81 mmol) and palladium tetrakis (49 mg, 0.04 mmol). The crude was purified over silica gel flash chromatography (EP:AcOEt 95:5) to afford **48** as pure compound (130 mg, yield 56%). ^{1}H-NMR (401 MHz, $CDCl_3$-d) δ 8.69 (ddd, J = 8.5, 1.5, 0.7 Hz, 1H), 8.33 (s, 1H), 8.16 (d, J = 8.5 Hz, 1H), 7.77 - 7. 68 (m, 3H), 7.62 - 7.52 (m, 2H), 7.48 - 7.38 (m, 2H), 6.80 (d, J = 3.6 Hz, 1H), 4.06 (s, 3H), 3.85 (s, 3H) ppm. UHPLC-MS (ESI, m/z) R_t = 3.88 min, 344 $[M+H]^+$

Methyl 2-(5-phenylfuran-2-yl)quinoline-4-carboxylate (49) (Scheme 1A) Compound **49** was obtained via general procedure D using brominated intermediate **27c** (100 mg, 0.3 mmol), boronic acid **31** (55 mg, 0.451 mmol), K_2CO_3 (125 mg, 0.9 mmol) and palladium tetrakis (17 mg, 0.015 mmol). The crude was purified over silica gel flash chromatography (EP: AcOEt, 95:5) to afford **49** as pure compound (70 mg, yield 71%). ^{1}H-NMR (401 MHz, DMSO-d_6) δ 8.67 (dd, J = 8.4, 1.5 Hz, 1H), 8.00 (s, 1H), 7.97 - 7.93 (m, 1H), 7.89 (dd, J = 8.3, 1.2 Hz, 2H), 7.67 (ddd, J = 8. 4, 6.8, 1.5 Hz, 1H), 7.55 - 7.43 (m, 3H), 7.40 (d, J = 3.5 Hz, 1H), 7.39 - 7.31 (m, 1H), 7.20 (d, J = 3.6 Hz, 1H), 4.01 (s, 3H) ppm. UHPLC-MS (ESI, m/z) R_t = 3.97 min, 330 $[M+H]^+$

6-Nitro-2-(5-phenylfuran-2-yl)quinoline-4-carboxylic acid (53) (Scheme 1B) Compound **53** was synthesized via general procedure A using 5-nitroisatin **51** (0.17 mg, 0.885 mmol), 5-phenyl-2-acetyl furan **52** (0.181 g, 0.973 mmol) and KOH (0.149 g, 2.65 mmol). After work-up, **53** was obtained as pure compound (0.087 g, yield 28%). The product contained a 10% impurity corresponding to the reduced amine form **17**. The

characterization reported refers to the desired compound **54**. ^{1}H-NMR (600 MHz, DMSO-d_6) δ 10.62 (d, J = 1.9 Hz, 1H), 8.22 (s, 1H), 8.05 (dd, J = 9.1, 2.4 Hz, 1H), 8.02 - 7.94 (d, J = 9.1 Hz, 1H), 7. 92 - 7.88 (m, 2H), 7.82 - 7.77 (m, 2H), 7.56 - 7.49 (d, J = 3.6 Hz, 1H), 7.42 (d, J = 3.6 Hz, 1H), 7.09 (d, J = 3.5 Hz, 1H) ppm. UHPLC-MS (ESI, m/z) R_t = 3.4 min, 361 $[M+H]^+$

6-Fluoro-2-(furan-2-yl)quinoline-4-carboxylic acid (61) (Scheme 2A) Compound **61** was obtained via general procedure F using 4-fluoroaniline **54** (0.5 mL, 5.4 mmol), furfural **58** (0. 49 mL, 6 mmol), complexed boron-diethyl ether trifluoride **59** (0.185 mL, 1.5 mmol) and pyruvic acid **60** (0.21 mL, 3 mmol). After work-up, **61** was obtained as pure compound (173 mg, yield 67%). ^{1}H-NMR (600 MHz, DMSO-d_6) δ 8.42 (dd, J = 11.0, 2.9 Hz, 1H), 8.35 (s, 1H), 8.13 (dd, J = 9.3, 5.8 Hz, 1H), 7.94 (dd, J = 1. 8, 0.8 Hz, 1H), 7.73 (dd, J = 9.3, 8.1, 2.9 Hz, 1H), 7.42 (dd, J = 3.5, 0.8 Hz, 1H), 6.72 (dd, J = 3.4, 1.8 Hz, 1H) ppm. UHPLC-MS (ESI, m/z) R_t = 2.59 min, 258 $[M+H]^+$

2-(Furan-2-yl)-6-(trifluoromethyl)quinoline-4-carboxylic acid (62) (Scheme 2A) Compound **62** was obtained via general procedure F using 4-trifluoromethylaniline **55** (0.223 mL, 1.8 mmol), furfural **58** (0. 166 mL, 2 mmol), complexed boron-diethyl ether trifluoride **59** (0.062 mL, 0.5 mmol) and pyruvic acid **60** (0.07 mL, 1 mmol). After work-up, **62** was obtained as pure compound (235 mg, yield 76%). ^{1}H-NMR (401 MHz, $CDCl_3$-*d*) δ 9.19 (s, 1H), 8.49 (s, 1H), 8.23 (d, J = 8.9 Hz, 1H), 7.96 (dd, J = 8.9, 2. 1 Hz, 1H), 7.82 (dd, J = 1.8, 0.8 Hz, 1H), 7.45 (dd, J = 3.5, 0.8 Hz, 1H), 6.70 (dd, J = 3.5, 1.8 Hz, 1H) ppm. Rt= 3.07 min, 308 $[M+H]^+$

6-Ethyl-2-(furan-2-yl)quinoline-4-carboxylic acid (63) (Scheme 2A) Compound **63** was obtained via general procedure F using 4-ethylaniline **56** (0.45 mL, 3.6 mmol), furfural **58** (0. 33 mL, 4 mmol), complexed boron-diethyl ether trifluoride **59** (0.123 mL, 1 mmol) and pyruvic acid **60** (0.141 mL, 2 mmol). After work-up, **63** was obtained as pure compound (228 mg, yield 43%). ^{1}H-NMR (401 MHz, $CDCl_3$-*d*) δ 8.52 (d, J = 1.9 Hz, 1H), 8.32 (s, 1H), 8.01 (d, J = 8.7 Hz, 1H), 7.79 (dd, J = 1.8, 0.7 Hz, 1H), 7. 68 (dd, J = 8.7, 2.0 Hz, 1H), 7.45 - 7.32 (m, 1H), 6.68 (dd, J = 3.5, 1.8 Hz, 1H), 2.84 (q, J = 7.6 Hz, 2H), 1.32 (t, J = 7.6 Hz, 3H) ppm. UHPLC-MS (ESI, m/z) Rt = 2.61 min, 268 $[M+H]^+$

2-(Furan-2-yl)-6-methoxyquinoline-4-carboxylic acid (64) (Scheme 2A) Compound **64** was obtained via general procedure F using 4-methoxyaniline **57** (0.222 g, 1.8 mmol), furfural **58** (0. 166 mL, 2 mmol), complexed boron-diethyl ether trifluoride **59** (0.062 mL, 0.5 mmol) and pyruvic acid **60** (0.07 mL, 1 mmol). After work-up, **64** was obtained as pure compound (170 mg, yield 63%). ^{1}H-NMR (401 MHz, DMSO-d_6) δ 8.26 (s, 1H), 8.11 (d, J = 2.9 Hz, 1H), 7.97 (d, J = 9.2 Hz, 1H), 7.90 (d, J = 1. 7 Hz, 1H), 7.46 (dd, J = 9.2, 2.9 Hz, 1H), 7.31 (d, J = 3.4 Hz, 1H), 6.69 (dd, J = 3.4, 1.8 Hz, 1H), 3.88 (s, 3H) ppm. UHPLC-MS (ESI, m/z) R_t = 2.3 min, 270 $[M+H]^+$

Methyl 6-fluoro-2-(furan-2-yl)quinoline-4-carboxylate (65b) (Scheme 2A) Compound **65b** was obtained via general procedure B using quinoline **61** (0.31 g, 1.21 mmol) and $SOCl_2$ (0.265 mL, 3.63 mmol). **65b** was obtained as pure compound (246 mg, yield 75%). ^{1}H-NMR (401 MHz, DMSO-d_6) δ 8.42 (dd, J = 11.0, 2.9 Hz, 1H), 8.35 (s, 1H), 8.13 (dd, J = 9.3, 5.8 Hz, 1H), 7.94 (dd, J = 1.8, 0. 8 Hz, 1H), 7.73 (dd, J = 9.3, 8.1, 2.9

Hz, 1H), 7.42 (dd, J = 3.5, 0.8 Hz, 1H), 6.72 (dd, J = 3.4, 1.8 Hz, 1H), 3.90 (s, 3H) ppm. UHPLC-MS (ESI, m/z). R_t = 3.36 min, 272 $[M+H]^+$

Methyl 2-(5-bromofuran-2-yl)-6-fluoroquinoline-4-carboxylate (65c) (Scheme 2A) Compound **65c** was obtained via general procedure C using intermediate ester **65b** (560 mg, 2.07 mmol) and *N*-bromo succinimide (554 mg, 3.11 mmol). The crude was purified over silica gel flash chromatography (EP: EtOAc, 95:5) to afford **65c** as pure compound (502 mg, yield 70%). ^{1}H-NMR (401 MHz, DMSO-d_6) δ 8.42 (dd, J = 11.0, 2.9 Hz, 1H), 8.35 (s, 1H), 8.13 (dd, J = 9.3, 5.8 Hz, 1H), 7.73 (ddd, J = 9.3, 8.1, 2.9 Hz, 1H), 7.42 (d, J = 3.5 Hz, 1H), 6.72 (d, J = 3.4 Hz, 1H) ppm. UHPLC-MS (ESI, m/z) R_t = 3.66 min, 350-352 $[M+H]^+$

Methyl 2-(furan-2-yl)-6-(trifluoromethyl)quinoline-4-carboxylate (66b) (Scheme 2A) Compound **66b** was obtained via general procedure B using quinoline **62** (0.245 g, 0.798 mmol) and $SOCl_2$ (0.175 mL, 2.39 mmol). **66b** was obtained as pure compound (130 mg, yield 51%). ^{1}H-NMR (401 MHz, $CDCl_3$-*d*) δ 9.19 (s, 1H), 8.49 (s, 1H), 8.23 (d, J = 8.9 Hz, 1H), 7.96 (dd, J = 8.9, 2.1 Hz, 1H), 7. 82 (dd, J = 1.8, 0.8 Hz, 1H), 7.45 (dd, J = 3.5, 0.8 Hz, 1H), 6.70 (dd, J = 3.5, 1.8 Hz, 1H), 3.90 (s, 3H) ppm. R_t = 3.59 min, 322 $[M+H]^+$

Methyl 2-(5-bromofuran-2-yl)-6-(trifluoromethyl)quinoline-4-carboxylate (66c) (Scheme 2A) Compound **66c** was obtained via general procedure C using intermediate ester **66b** (300 mg, 0.94 mmol) and *N*-bromo succinimide (249.31 mg, 1.40 mmol). The crude was purified over silica gel flash chromatography (EP: EtOAc, 95:5) to afford **66c** as pure compound (130 mg, yield 35%). ^{1}H-NMR (401 MHz, $CDCl_3$-*d*) δ 9.09 (dt, J = 2.0, 0.9 Hz, 1H), 8.37 (d, J = 1.1 Hz, 1H), 8.20 (dq, J = 8.9, 0. 8 Hz, 1H), 7.88 (dd, J = 8.9, 2.0 Hz, 1H), 7.31 (d, J = 3.5 Hz, 1H), 6.55 (d, J = 3.5 Hz, 1H), 4.08 (s, 3H) ppm. UHPLC-MS (ESI, m/z) R_t = 3.84 min, 400-402 $[M+H]^+$

Methyl 6-ethyl-2-(furan-2-yl)quinoline-4-carboxylate (67b) (Scheme 2A) Compound **67b** was obtained via general procedure B using quinoline **63** (0.377 g, 1.41 mmol) and $SOCl_2$ (0.309 mL, 4.23 mmol). **67b** was obtained as pure compound (274 mg, yield 70%). ^{1}H-NMR (401 MHz, $CDCl_3$-*d*) δ 8.52 (d, J = 1.9 Hz, 1H), 8.32 (s, 1H), 8.01 (d, J = 8.7 Hz, 1H), 7.79 (dd, J = 1.8, 0.7 Hz, 1H), 7.68 (dd, J = 8. 7, 2.0 Hz, 1H), 7.45 - 7.32 (m, 1H), 6.68 (dd, J = 3.5, 1.8 Hz, 1H), 3.90 (s, 3H), 2.84 (q, J = 7.6 Hz, 2H), 1.32 (t, J = 7.6 Hz, 3H) ppm. UHPLC-MS (ESI, m/z) R_t = 3.52 min, 282 $[M+H]^+$

Methyl 2-(5-bromofuran-2-yl)-6-ethylquinoline-4-carboxylate (67c) (Scheme 2A) Compound **67c** was obtained via general procedure C using intermediate ester **67b** (274 mg, 0.98 mmol) and *N*-bromo succinimide (261.65 mg, 1.47 mmol). The crude was purified over silica gel flash chromatography (EP: EtOAc, 95:5) to afford **67c** as pure compound (241 mg, yield 75%). ^{1}H-NMR (401 MHz, DMSO-d_6) δ 8.31 (s, 1H), 8.20 (s, 1H), 8.01 (d, J = 8.7 Hz, 1H), 7.72 (dd, J = 8.6, 2.0 Hz, 1H), 7. 45 (d, J = 3.5 Hz, 1H), 6.85 (d, J = 3.5 Hz, 1H), 3.99 (s, 3H), 2.80 (q, J = 7.6 Hz, 2H), 1.25 (t, J = 7.6 Hz, 3H) ppm. UHPLC-MS (ESI, m/z) R_t = 3.64 min, 362-364 $[M+H]^+$

Methyl 2-(furan-2-yl)-6-methoxyquinoline-4-carboxylate (68b) (Scheme 2A) Compound **68b** was obtained via general procedure B using quinoline **64** (0.17 g, 0.631 mmol) and $SOCl_2$ (0.138 mL, 1.89 mmol). **68b** was obtained as pure compound (100 mg, yield 56%). ^{1}H-NMR (401 MHz, DMSO-*d_6*) δ 8.26 (s, 1H), 8.11 (d, J = 2.9 Hz, 1H), 7.97 (d, J = 9.2 Hz, 1H), 7.90 (d, J = 1.7 Hz, 1H), 7. 46 (dd, J = 9.2, 2.9 Hz, 1H), 7.31 (d, J = 3.4 Hz, 1H), 6.69 (dd, J = 3.4, 1.8 Hz, 1H), 3.90 (s, 3H), 3.88 (s, 3H) ppm. UHPLC-MS (ESI, m/z) R_t = 3.26 min, 284 $[M+H]^+$

Methyl 2-(5-bromofuran-2-yl)-6-methoxyquinoline-4-carboxylate (68c) (Scheme 2A) Compound **68c** was obtained via general procedure C using intermediate ester **68b** (250 mg, 0.883 mmol) and *N*-bromo succinimide (235.61 mg, 1.32 mmol). The crude was purified over silica gel flash chromatography (EP: EtOAc, 95:5) to afford **68c** as pure compound (234 mg, yield 73%). ^{1}H-NMR (401 MHz, DMSO-*d_6*) δ 8.25 (s, 1H), 8.06 - 8.02 (m, 2H), 7.53 (dd, J = 9.3, 2.8 Hz, 1H), 7.41 (d, J = 3.6 Hz, 1H), 6.86 (d, J = 3.5 Hz, 1H), 4.02 (s, 3H), 3.92 (s, 3H) ppm. UHPLC-MS (ESI, m/z) R_t = 3.85 min, 360-362 $[M+H]^+$

Methyl 6-fluoro-2-(5-phenylfuran-2-yl)quinoline-4-carboxylate (69) (Scheme 2A) Compound **69** was obtained via general procedure D using brominated intermediate **65c** (100 mg, 0.29 mmol), boronic acid **31** (53.65 mg, 0.44 mmol), K_2CO_3 (118.5 mg, 0.87 mmol) and palladium tetrakis (7.33 mg, 0.015 mmol). The crude was purified over silica gel flash chromatography (DCM: PE, 85:15) to afford **69** as pure compound (85 mg, yield 85%). ^{1}H-NMR (401 MHz, $CDCl_3$-*d*) δ 8.54 - 8.42 (m, 2H), 8.22 (s, 1H), 7.91 - 7.79 (m, 2H), 7.58 - 7.41 (m, 4H), 7.34 (t, J = 7.4 Hz, 1H), 6.88 (d, J = 3.4 Hz, 1H), 4.09 (s, 3H) ppm. UHPLC-MS (ESI, m/z) Rt = 3.86 min, 348 $[M+H]^+$

Methyl 2-(5-phenylfuran-2-yl)-6-(trifluoromethyl)quinoline-4-carboxylate (70) (Scheme 2A) Compound **70** was obtained via general procedure D using brominated intermediate **66c** (115 mg, 0.29 mmol), boronic acid **31** (54 mg, 0.44 mmol), K_2CO_3 (118 mg, 0.87 mmol) and palladium tetrakis (17.33 mg, 0.015 mmol). The crude was purified over silica gel flash chromatography (EP: AcOEt, 95:5) to afford **70** as pure compound (70 mg, yield 62%). ^{1}H-NMR (401 MHz, $CDCl_3$-*d*) δ 9.08 (d, J = 1.9 Hz, 1H), 8.53 (s, 1H), 8.49 (d, J = 8.9 Hz, 1H), 7.94 (dd, J = 9.1, 2. 1 Hz, 1H), 7.89 - 7.79 (m, 3H), 7.51 - 7.44 (m, 2H), 7.42 - 7.35 (m, 1H), 6.93 (d, J = 3.7 Hz, 1H), 4.13 (s, 3H) ppm. UHPLC-MS (ESI, m/z) R_t = 4.03 min, 398 $[M+H]^+$

Methyl 6-ethyl-2-(5-phenylfuran-2-yl)quinoline-4-carboxylate (71) (Scheme 2A) Compound **71** was obtained via general procedure D using brominated intermediate **67c** (193 mg, 0.54 mmol), boronic acid **31** (98.76 mg, 0.81 mmol), K_2CO_3 (220.64 mg, 1.62 mmol) and palladium tetrakis (31.20 mg, 0.027 mmol). The crude was purified over silica gel flash chromatography (EP: AcOEt, 95:5) to afford **71** as pure compound (117 mg, yield 61%). ^{1}H-NMR (401 MHz, $CDCl_3$-*d*) δ 8.47 (s, 1H), 8.41 (s, 2H), 7.85 (dt, J = 8.1, 1.2 Hz, 2H), 7.67 (d, J = 8.9 Hz, 1H), 7.45 (t, J = 7.6 Hz, 3H), 7.35 (t, J = 7.4 Hz, 1H), 6.90 (d, J = 3.7 Hz, 1H), 4.11 (s, J = 1.2 Hz, 3H), 2.90 – 2.84 (q, J = 7.6 Hz, 2H), 1.37 – 1.33 (t, J = 7.6 Hz, 3H) ppm. UHPLC-MS (ESI, m/z) R_t = 4.03 min, 398 $[M+H]^+$

Methyl 6-methoxy-2-(5-phenylfuran-2-yl)quinoline-4-carboxylate (72) (Scheme 2A) Compound **72** was obtained via general procedure D using brominated intermediate **68c** (113 mg, 0.31 mmol), boronic acid **31** (57.06 mg, 0.47 mmol), K_2CO_3 (129.36 mg, 0.94 mmol) and palladium tetrakis (18.03 mg, 0.015 mmol). The crude was purified over silica gel flash chromatography (EP: AcOEt, 95:5) to afford **72** as pure compound (67 mg, yield 60%). ^{1}H-NMR (401 MHz, $CDCl_3$-*d*) δ 8.49 (s, 1H), 8.17 (d, J = 2.8 Hz, 1H), 7.84 (d, J = 7.8 Hz, 2H), 7.45 (t, J = 7.5 Hz, 5H), 7.34 (t, J = 7.6 Hz, 1H), 6.90 (d, J = 3.8 Hz, 1H), 4.11 (d, J = 0.9 Hz, 3H), 3.99 – 3.97 (m, 3H). UHPLC-MS (ESI, m/z) R_t = 3.79 min, 360 $[M+H]^+$

2-(5-Bromothiophen-2-yl)-6-methylquinoline-4-carboxylic acid (77) (Scheme 2C) Compound **77** was obtained via general procedure F using *p*-toluidine **75** (144 mg, 1.35 mmol), 5-bromothiophene-2-carbaldehyde **76** (286 mg, 1.5 mmol), complexed boron-diethyl ether trifluoride **59** (0.046 mL, 0.375 mmol) and pyruvic acid **60** (0.053 mL, 0.75 mmol). **77** was obtained as pure compound (113 mg, yield 83%). ^{1}H-NMR (401 MHz, DMSO-d_6) δ 8.36 (s, 1H), 8.32 (s, 1H), 7.96 – 7.89 (m, 2H), 7.66 (dd, J = 8.6, 1.9 Hz, 1H), 7.35 (d, J = 4.0 Hz, 1H), 2.52 (s, 3H) ppm. UHPLC-MS (ESI, m/z) R_t = 3.6 min, 348/350 $[M+H]^+$

Scheme S1. Synthesis of intermediate **52**

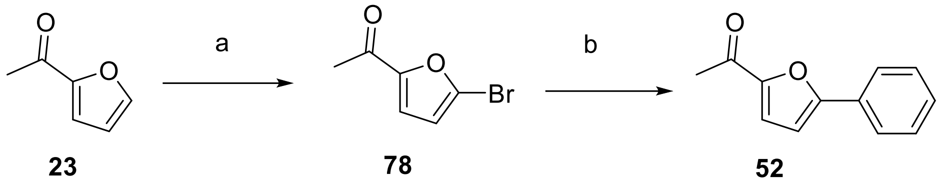

1-(5-bromofuran-2-yl)ethan-1-one (**78**) In a flask, 2-acetyl furan **23** (1 g, 9.08 mmol, 1.00 eq) was solubilized in 10 mL of DMF and *N*-bromo succinimide (1.94 g, 10.90 mmol, 1.20 eq) was added slowly at 0 °C. The reaction was left reacting at room temperature for 4 hrs. Then, the mixture was added dropwise to 50 mL of cold distilled water at 0°C under stirring. The formation of a sticky precipitate was observed. The aqueous solution is extracted with DCM (3x40 mL) and the organic solvent is dried over Na_2SO_4 and concentrated by vacuum. The crude was purified over silica gel flash column chromatography (EP: AcOEt, 98:2) to afford the pure compound **78** (710 mg, 3.76 mmol, yield 41%). ^{1}H NMR (600 MHz, DMSO) δ 7.50 (d, J = 3.6 Hz, 1H), 6.87 (d, J = 3.6 Hz, 1H), 2.39 (s, 3H). In agreement with Ismail, M. A.; Brun, R.; Wenzler, T.; Tanious, F. A.; Wilson, W. D.; Boykin, D. W. J. Med. Chem. 2004, 47, 3658;

1-(5-phenylfuran-2-yl)ethan-1-one (**52**) In a three-necked flask, the compound **78** (2 g, 10.58 mmol, 1.00 eq) was solubilized in dioxane: H_2O [10:1] 0.1 M). Phenyl boronic acid **31** (1.55 g, 12.7 mmol, 1.20 eq) and K_2CO_3 (2.92 g, 21.16 mmol, 2.00 eq) were added to the solution and the mixture was degassed with N_2. Palladium tetrakis (611 mg, 0.53 mmol, 0.05 eq) was then added and the reaction was conducted at 100 °C in inert atmosphere stirring for 2 hours. Then, the reaction was cooled to room temperature, 50 mL of water is added and the dioxane was evaporated by rotavapor. An extraction with DCM (3x50 mL) was performed. The organic phase was dried over Na_2SO_4, filtered and concentrated by vacuum. An automated Combi-flash chromatography column is conducted (SiO_2 gold 80 g; DCM: MeOH 8:2) to afford the desired **52** (1.2 g, 6.44 mmol, 61% yield) as dark orange oil. ^{1}H NMR (600 MHz, DMSO) δ 7.88 – 7.83 (m, 2H), 7.56 (d, J = 3.7 Hz, 2H), 7.53 – 7.47 (m, 1H), 7.45 – 7.39 (m, 1H), 7.21 (d, J = 3.7 Hz, 1H), 2.46 (s, 3H). UPLC-MS (ESI, m/z) Rt = 2.73 min, 173 $[M+H]^+$

Scheme S2. Synthesis of intermediate **74**

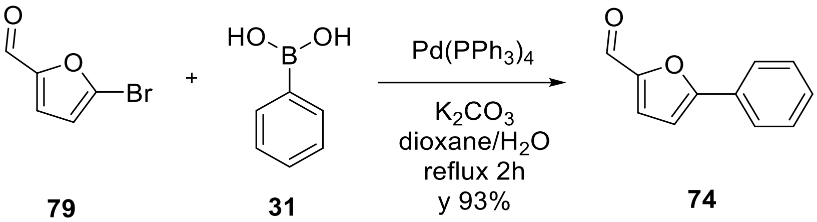

5-Phenylfuran-2-carbaldehyde (74) In a flask, 5-bromo-2-furaldehyde **79** (1 g, 1.00 equiv), phenyl boronic acid **31** (836 mg, 1.20 equiv) and potassium carbonate (1.60 g, 2.00 equiv) are dissolved and stirred in a mixture dioxane:H_2O. The mixture is bubbled under nitrogen for 20 min. Then palladium tetrakis catalyst (334 mg, 0.05 equiv) is added. The reaction is refluxed for two hours. After that time, the reaction mixture is diluted with ethyl acetate (20 mL). The organic phase is washed with H_2O (3x 10 mL), dried over Na_2SO_4 and evaporated to dryness. The crude is purified over silica gel flash chromatography (EP: EA, elution with 15% EA) to give pure compound **74** as orange solid (913 mg, yield 93%). ^{1}H NMR (600 MHz, $CDCl_3$-*d*) δ 9.66 (s, 1H), 7.83 (dd, *J* = 8.4, 1.2 Hz, 2H), 7.48 – 7.43 (m, *J* = 8.2, 6.7 Hz, 2H), 7.42 – 7.38 (m, *J* = 7.4 Hz, 1H), 7.33 (d, *J* = 3.7 Hz, 1H), 6.85 (d, *J* = 3.7 Hz, 1H). . UPLC-MS (ESI, m/z) @254 nm Rt = 2.76 min, 173 $[M+H]^+$ In agreement with M. S. McClure, B. Glover, E. McSorley, A. Millar, M. H. Osterhout, F. Roschangar, Organic letters 2001, Vol. 3, No. 111677-1680.

Kinetic solubility studies

The purpose of this study is to determine the aqueous kinetic solubility of compounds from 10 mM DMSO stock solution in Phosphate Buffered Saline (PBS) at pH 7.4 . The study is performed by incubating an aliquot of 10 mM DMSO stock solution in PBS (pH 7.4) at a target concentration of 250µM resulting in a final concentration of 2.5% DMSO. The incubation is carried out under shaking at 25°C for 24h followed by centrifugation at 14.800rpm (21100g) for 30 min. The supernatant is analyzed by UPLC/MS for the quantification of dissolved compound (in µM) by UV at a specific wavelength (215nm). The UPLC/MS analyses are performed on a Waters ACQUITY UPLC/MS system consisting of a SQD (single quadrupole detector) Mass Spectrometer equipped with an Electrospray Ionization interface and a Photodiode Array Detector. The PDA range is 210-400nm. Electrospray ionization in positive mode is used in the mass scan range 100-500Da. The analyses are run on an ACQUITY UPLC BEH C18 column (100x2.1mmID, particle size 1.7µm) with a VanGuard BEH C18 pre-column (5x2.1mmID, particle size 1.7µm), using H2O + 0.1% HCOOH (A) and MeCN + 0.1% HCOOH (B) as mobile phase. Solubility range: High >100 µM, Medium 10–100 µM, Low 0–10 µM

S3. Biophysical, biochemical and biological experiments

Materials and methods

ELISA assay

A competitive ELISA screening assay using biotinylated BRC4 peptide to disrupt the BRC4-RAD51 interaction was performed by modifying the method described by Rajendra *et al.*[3] Recombinant Human RAD51 protein, fused to GST-tag, was expressed in BL21 DE3 Gold E. coli and purified by GSTrap FF column (AKTA Instrument GE-Healthcare), US. GST-tag was removed using the Thrombin (GE Healthcare) before the elution. Eluted protein was quantified (BCA, ThermoFisher) and checked by SDS gel stained with Coomassie brilliant blue. BRC4-biotinylated peptide (N-term Biotin KEPTLLGFHTASGKKVKI AKESLDKVKNLFDEKEQ from Life Technologies) was used to coat 384-well plates (Nunc). After washing with PBS containing 0.05% Tween-20 (PBST), and blocking with the solution BSA 1%/PBST, overnight hybridization with human RAD51 protein. Test compounds were added in dose-response from 0.01 to 100mMin triplicate with constant DMSO 1%. Antibody raised against Rad51 (Millipore) and HRP-secondary antibody staining to develop the 3,30,5,50-tetramethyllbenzidine signal (Sigma) quenched with 1M HCl was used as the assay readout. Colorimetric measure was read on Victor5 (PerkinElmer) plate Reader. Unbiotinylated BRC4 and RAD51 were included in the assay as positive control. Results were analyzed using GraphPad Software. To calculate E_{max}% values, CAM833, a known RAD51-BRCA2 disruptor developed by Scott *et al.*[4] was used as internal reference. The procedure was previously reported in Myers S.H. *et al.*[5] Results are reported in Table 1: ELISA assay results are expressed as EC_{50} (all points tested in triplicate with error bars indicating the standard deviation) and as E_{max} % percentage (the maximum activity of the tested compound and compared to the activity of CAM833[4] at the same concentration).

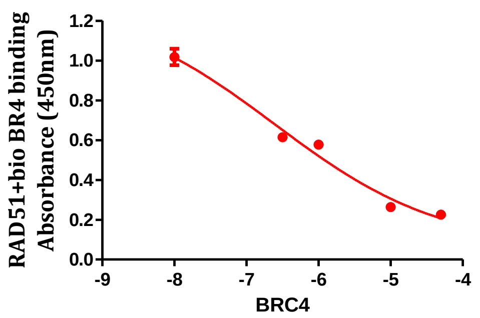

Log [untagged-BRC4]M	Absorbance RAD51+bio-BRC4		
-8,000	0,9350	1,0570	1,0620
-6,500	0,6120	0,6180	0,6150
-6,000	0,5540	0,6010	0,5780
-5,000	0,2520	0,2550	0,2840
-4,300	0,2270	0,2200	0,2280
BLK	0,2220	0,2290	0,2290

Calibration curve of the displacement of RAD51 from biotynilated-BRC4 with scalar doses of soluble untagged-BRC4: On the right, the table reports the raw data of absorbance measured at each concentration of untagged-BRC4: The developed ELISA procedure includes the immobilization of biotinylated-BRC4 on the wells surface of ELISA plate, and its following interaction with RAD51 in soluble form. This procedure aims at estimating the power of compounds in inhibiting the interaction RAD51-BRC4, and the quantitative value of the inhibitory efficacy is obtained with the aid of a calibration curve. By using scalar amounts of soluble, untagged-BRC4, we obtained a standard curve for quantifying the residual interaction between the

immobilized biotinylated-BRC4 peptide and RAD51. Soluble untagged-BRC4 can displace the interaction between RAD51 and the immobilized biotinylated-BRC4 and, as a consequence, the higher is the amount of the added untagged-BRC4, the lower is the RAD51 concentration detected in wells.

Protocol for the Expression and Purification of His-hRAD51.

His-hRAD51 expression and purification were carried out as previously reported[1] with minor modifications. hRAD51 was expressed in E. coli Rosetta2(DE3) cells. A saturated overnight culture of Rosetta2 (DE3)/pET15b-His-hRAD51 was diluted (1:1000) into a fresh TB-5052 autoinduction medium containing ampicillin (100 µg/mL). The flasks were shaken at 200 rpm at 20 °C for 72 h. The pellet was subsequently resuspended in an appropriate volume of buffer A (20 mM Tris-HCl (pH 8.00), 500 mM NaCl, 10 mM imidazole, 10% (v/v) glycerol) supplemented with protease inhibitor cocktail (SIGMA-FAST protease inhibitor cocktail tablets, EDTA-50 free). The cell suspension was lysed on ice through sonication (10 rounds of 1 min.; amplitude 85%; Tip KE76; Bandelin Sonoplus HD2070 sonicator). The disrupted cell suspension was centrifuged for 30 min at 20,000 g. The supernatant fraction was filtered with a 0.45 µm (MiniSart syringe filter 0.45 µm) membrane to remove residual particulates before chromatography. The supernatant was applied onto a HisTrap HP chromatography column (Cytiva), equilibrated with buffer A. A wash step was performed using 10% of buffer B (20 mM Tris-HCl (pH 8.00), 500 mM NaCl, 500 mM imidazole, 10% (v/v) glycerol). The protein was then eluted with a linear gradient from 10% to 100% of buffer B over 10 column volumes. Fractions (0.5 mL) were collected and analysed by SDS–PAGE. Collected fractions corresponding to the recombinant protein were dialyzed overnight at 4 °C against buffer C (50 mM Tris-HCl (pH 8.00), 200 mM KCl, 0.25 mM EDTA, 2 mM DTT, 10% (v/v) glycerol). Dialyzed protein was loaded onto RESOURCE Q anion exchange chromatography column (Cytiva) equilibrated in buffer C. The elution was performed with a linear gradient of buffer D (50 mM Tris-HCl (pH 8.00), 1 M KCl, 0.25 mM EDTA, 2 mM DTT, 10% (v/v) glycerol). Fractions (0.5 mL) were collected and analysed by SDS–PAGE. Fractions containing His-hRAD51 were pooled and dialyzed against the storage buffer (20 mM HEPES (pH 8.00), 250 mM KCl, 0.1 mM EDTA, 2 mM DTT, 10% (v/v) glycerol). The protein yield was determined from the optical absorption at 280 nm (extinction coefficient 14 900 M–1 cm–1) of the final sample.

Microscale thermophoresis

Microscale thermophoresis analyses were performed on a Monolith NT.115Pico instrument (NanoTemper Technologies, München, Germany). The labelling of hRAD51 recombinant protein was performed with the Monolith His-Tag labelling kit RED-tris-NTA 2nd Generation kit (NanoTemper Technologies). To determine a concentration-dependent MST binding curve, MST measurements were performed at 10-15% excitation and medium power on 16 premium capillaries containing a constant concentration (50 nM) of labelled His-hRAD51 protein and 16 different concentrations of the compounds. The highest concentration tested was 300 µM. Measurements were carried out in the following buffer: 20 mM

HEPES (pH 8.00), 250 mM KCl, 0.1% (v/v) Pluronic® F-127, 0.1% (v/v) PEG 8000, 5% (v/v) glycerol, 5% DMSO. Binding curves were fitted using the Affinity Analysis software of Nanotemper Technologies and analyzed at 1.5 seconds to obtain binding affinity data. The average of three independent binding curves were re-graphed and fitted using GraphPad Prism 10.2.3 (GraphPad Software, San Diego, CA, USA).

NMR binding and competition experiments

All NMR spectra were recorded on a Bruker NMR 600 MHz Neo spectrometer equipped with a 5 mm CryoProbe™ QCI $^{1}H/^{19}F–^{13}C/^{15}N–D$ quadruple resonance, a shielded z-gradient coil, and a SampleJet™ automatic sample changer with temperature control. Prior to binding experiments, the solubility of compound **19** was evaluated using the SPAM filter approach[6], testing concentrations of 50, 100 and 200 μM in the presence of 200 μM 4-trifluoromethyl benzoic acid (internal reference) in the binding assay buffer (20 mM Hepes pH 8, 250 mM KCl, 0.1 mM EDTA, 2 mM $MgCl_2$, 2 mM DTT 1% Glycerol, 20% D_2O, for the lock signal). For each sample, a 1D ^{1}H NMR spectrum was recorded using a standard NOESY (nuclear Overhauser effect spectroscopy) presaturation water suppression pulse sequence, with 64 k data points, a 30 ppm spectral width (sw) , 128 scans, an acquisition time (aq) of 1.835 s, a relaxation delay (d1) of 4 s, and a mixing time of 10 ms. In the binding and displacement experiments, compound **19** was evaluated at a fixed concentration (50 μM) under three conditions: alone, with RAD51 (1 μM), and with RAD51 in the presence of increasing amounts (2 μM and 5 μM) of the BRC4 peptide. WaterLOGSY[7] experiments, were used to assess ligand binding behavior, and the resulting spectra were recorded and processed in standard conditions (7.5 ms long 180° Gaussian-shaped pulse, aq 0.852 s, mixing time of 1.7 s, relaxation delay 2 s, lb 1 Hz).

In presence of protein, the NMR signals of compound **19** change from negative (absence of protein, red spectrum) to positive (blue spectrum), confirming the binding of the compound to RAD51. These positive signals decrease proportionally with increasing BRC4 concentrations (green and violet spectra) suggesting competition between the compound and the peptide. The WaterLOGSY signal of a non-significant buffer impurity (*) at approximately 8.4 pmm does not change in the presence of either protein or peptide, as it does not bind to RAD51.

Cell Cultures

BxPC-3, HPAC and CAPAN-1 cells were grown in RPMI 1640 (Merck, R0883) supplemented with 10% FBS, 100 U/mL penicillin/streptomycin, and 2 mM glutamine. The HEK-293 cell line was grown in DMEM High Glucose (Merck, D6546) supplemented with 10 % FBS, 100 U/mL penicillin/streptomycin, and 2 mM glutamine. All cell lines were obtained from American Type Culture Collection (ATCC, Manassas, VA, USA). The human primary pancreatic epithelial cell line (Cell Biologics, H6037) was grown in its specific medium (Cell Biologics, H6621) supplemented with epithelial cell growth supplement (Cell Biologics, H6621-Kit). All the cell line cultures were routinely tested for Mycoplasma contamination.

Homologous Recombination Quick Assay (HR-QA)

Homologous recombination (HR) was assessed using a commercially available kit (Norgen, 35600) as previously described.[8] This assay is based on cell transfection with two plasmids able to recombine upon cell entry. The efficiency of HR was assessed by Real-Time PCR, using primer mixtures included in the assay kit. Different primer mixtures allow differentiation between the original plasmid backbones and their recombination product. BxPC3 cells (2×10^5 per well) were seeded in a 24-well plate and allowed to adhere overnight. Co-transfection with the two plasmids (1µg each) was performed in Lipofectamine2000 (Invitrogen, 11668019), according to the manufacturer's instructions. During the 5 h of transfection, cells were exposed to different doses of compounds, dissolved in DMSO. After washing with PBS, cells were harvested, and DNA was isolated using QIAamp DNA Mini kit (Qiagen, 51304). Sample concentration was measured using an ONDA Nano Genius photometer. The efficiency of HR was assessed by Real-Time PCR, using 25 ng of template and primer mixtures included in the assay kit, following the protocol indicated by the manufacturer. Data analysis was based on the $2^{-\Delta\Delta Ct}$ method: (Recombination Product/Backbone Plasmids) treated versus (Recombination Product/Backbone Plasmids) control. In multidose experiments, the value of IC_{50} was extrapolated using least squares regression (R^2). HR inhibition results are expressed as a percentage of the maximum inhibition observed at the concentration shown in brackets, as shown in Table 1.

mClover-based Homologous Recombination assay (mCL-HR)

The mCL-HR assay was performed as detailed in Myers *et al*.[5] Different compound doses were added to HEK-293 cells grown on coverslips in a 24-well plate 1 h before transfection. Cells were transfected with 500 ng sgRNA plasmid targeting Lamin A (pUC CBA SpCas9. EF1a-BFP.sgLMNA, Addgene Plasmid, 98971) and 500 ng donor plasmid (pCAGGS Donor mClover-LMNA, Addgene Plasmid, 98970) using Lipofectamin2000 (Invitrogen, 11668019). The next day, cell culture media was replaced by fresh media containing the desired compound doses. Three days after transfection, cells were fixed in PBS containing 4% formalin for 15 min and washed twice with PBS before mounting. Images were acquired using a Nikon fluorescent microscope equipped with filters for FITC/TRITC/DAPI and were subsequently analyzed. The number of fluorescent cells (mClover$^+$ cells) was compared to the total amount of cells evaluated by DAPI. Images were analyzed by two independent observers analyzing approximately 100 cells for each treatment sample. Data were analyzed by one-way ANOVA with Tukey's post-test. Results reported in Figure 4D Results are mean values ± SD (n = 3). A significant decrease was observed at ≥ 20 µM (*p = 0.04; **p < 0.01).

Cells immunofluorescence assay

Immunofluorescence was used for studying RAD51 nuclear translocation and for evidencing an increase in γ-H2AX inside the nucleus following the treatment with compounds of interest as previously described.[1, 5] To visualize RAD51 in cell nuclei, BxPC-3 cells were seeded on glass coverslips placed in a 6-well culture plate (2×10^5 cells/well) and allowed to adhere overnight. Cultures were then preincubated with a specific dose of the target compound for 1 h and subsequently exposed to 50 µM cisplatin for an additional 1.5 h. Medium was removed, and cells were maintained in the presence of the compound for 5 h. To visualize γ-

H2AX, BxPC-3 cells were seeded on glass coverslips placed in a 6-well culture plate (13×10^4 cells/well) and allowed to adhere overnight. The next day, cells were treated with the desired compound for 72 h. At the end of cell treatment, cultures growing on coverslips were fixed in PBS containing 4% formalin for 13 min, permeabilized in 70% ethanol, air-dried, and washed twice with PBS. Samples were incubated in 5% bovine serum albumin (BSA) in PBS for 30 min and subsequently exposed to anti-RAD51 rabbit monoclonal antibody (1:1000 in 5% BSA/PBS, BioAcademia, 70-001) or anti-γ-H2AX [phospho-139] rabbit polyclonal (1:2000 in BSA/PBS, Abcam, 11174) overnight at 4 °C. After washing, coverslips were incubated with a secondary anti-rabbit rhodamine-labelled (1:1000 in 5% BSA/PBS, Novus Biologicals, NB120-6792), for 30 min, washed, air dried, and mounted with a solution 2 µg/mL DAPI in DABCO. Images were acquired using a Nikon fluorescent microscope equipped with filters for FITC, TRITC and DAPI. The percentage of cells bearing RAD51 or γ-H2AX nuclear foci was estimated by two independent observers analyzing approximately 200 cells for each treatment sample and comparing it to the corresponding DAPI. Data were statistically analyzed by one-way ANOVA followed by Tukey's multiple comparisons test. In CPL-exposed cultures, RAD51 labelling was observed in 56% of nuclei (*** $p = 0.0003$, compared to controls). This value dropped to 22% in cells exposed to both **19** and CPL (**$p = 0.001$, compared to cultures exposed to the single CPL treatment). Data were statistically evaluated by one-way ANOVA followed by Tukey's multiple comparisons test. The association of **19** with ola led to a statistically significant increased micronuclei generation, compared to that caused by the single ola administration. (p *** = 0.0001).

Cell viability assay

Cell viability was assessed with the CellTiter-Glo luminescent cell viability assay from Promega (G7571) as previously carried out.[1, 5] For this experiment, 1×10^3, 5×10^3 or 2×10^4 cells, depending on the cell line, were seeded in 200 µL of culture medium into each well of a 96-multiwell white body plate and allowed to adhere overnight. After 144 h incubation in the presence of PARPi and the RAD51-BRCA2 inhibitor alone or in combination, the plate was allowed to equilibrate at room temperature for 30 min and the CellTiter-Glo reactive was directly added to each well. The plate was kept on a shaker for 10 min to induce cell lysis, and its luminescence was measured with a Fluoroskan Ascent FL reader (Labsystems). Data were statistically evaluated by two-way ANOVA, followed by Tukey's multiple comparisons test. Results shown in Figure 5 are mean ± SD (n = 3). (****$p < 0.0001$).

Interaction Index

The interaction index (I. index) between PARPi and RAD51-BRCA2 inhibitor was assessed by applying the following formula, as reported in Ferreira D.S. *et al* [9] and Falchi F. *et al* [10]: (Surviving cells treated with the combination) / [(Surviving cells treated with PARPi) × (Surviving cells treated with RAD51/BRCA2 inhibitor)] × 100. According to ref. 46 and 47, a result ranging from 0.8 to 1.2 denotes an additive effect. Synergism is indicated by a result < 0.8. A result > 1.2 indicates an antagonistic effect. Results are shown in

Table 1. Figure 5 shows results of the combination studies of **19** and olaparib: BxPC-3: 0.77 (40 µM); HPAC: 0.72 (40 µM); CAPAN-1: >0.8 (40 µM).

Clonogenic assay

The clonogenic assay was carried out adapting the protocol described in Rossi V. *et al.*[11] BxPC-3 (5×10^2/well) were seeded into six-well plates and allowed to adhere overnight. The day after cells were re-fed with fresh media containing the desired concentrations of the studied drugs. After 24 h of treatment, the medium was changed, and cells were cultured for an additional 12 days in a drug-free medium. Subsequently, cells were fixed and stained with a solution of 0.5% crystal violet and 6% glutaraldehyde in phosphate-buffered saline (PBS) for 30 min. The remaining staining solution was removed, and the plate was washed with distilled water and dried at room temperature. Therefore, colonies were photographed. Finally, SDS 10% in H_2O was added to induce a complete dissolution of the crystal violet and absorbance was measured using a Multiskan EX plate reader with a wavelength of 570 nm. Data were statistically evaluated by one-way ANOVA, followed by Tukey's multiple comparisons test. A statistically significant reduction after ola/19 treatment was observed, compared to cells exposed to the single ola treatment (**** $p < 0.0001$).

Cell death inhibitor

Cell death assay was performed as described in Myers *et al.*[5] 5×10^3 BxPC-3 and 1×10^3 HPAC cells were seeded in a 96-multiwell plate and allowed to adhere overnight. The day after, cells were treated with PARPi and the RAD51-BRCA2 disruptor alone or in combination. 72 h later, 20 µM Z-VAD-FMK or 20 µM Nec-1 (Merck, Cat #627610; #480065), dissolved in DMSO, was added and then re-added to cultures every 24 h until the sixth day of treatment. The cell viability was assessed by using the same kit and procedure used for cell viability (CellTiter-Glo luminescent cell viability assay from Promega, G7571). Data were statistically evaluated using two-way ANOVA followed by Tukey's multiple comparisons test. Data were statistically evaluated using two-way ANOVA followed by Tukey's multiple comparisons test (**** $p < 0.0001$; *** $p = 0.005$; compared to the cultures treated with the **19**/ola association).

Western blot

Western blot analysis was performed as detailed in Masi M. *et al* [12]. Cell cultures (5×10^5 cells) were exposed for 144 h to PARPi given alone or in combination with the RAD51-BRCA2 disruptor. Cells were then lysed in 60 µL RIPA buffer containing protease inhibitor (Roche, 04693124001) and phosphatase inhibitor (Merck, 78420). The homogenates were left for 30 min on ice and then centrifuged for 15 min at 10000 g. 80 µg proteins of the supernatants (measured according to Bradford) were loaded into 12% polyacrylamide gel for electrophoresis. The separated proteins were blotted on a low fluorescent PVDF membrane (Cytiva Life Sciences, 10600060) using a standard apparatus for wet transfer with an electrical field of 60 mA for 16 h. The blotted membrane was blocked with 5% BSA in TBS-Tween and probed with the primary antibody. The antibodies used were: mouse anti-BCL-2 (1:200 in BSA/TBS tween, Santa Cruz

Biotechnology, sc-7382) and rabbit anti-BAX (1:1000 in BSA/TBS tween, Abcam, ab7977). Binding was revealed by Alexa Fluor 647 secondary antibody (1:1000 in TBS-Tween, donkey anti-mouse IgG, Jackson Immuno-Research Laboratories, 715-605-151) or Cy5-labelled secondary antibody (1:2500 in TBS-Tween, goat anti-rabbit-IgG, Jackson Immuno-Research Laboratories, 111-175-144). All incubation steps were performed according to the manufacturer's instructions. The fluorescence of the blots was assessed with the Pharos FX Scanner (Bio-Rad) at a resolution of 100 µm, using the Quantity One software (Bio-Rad). The intensity of the bands was evaluated using the ImageJ 1.53k software methods. Bands' densitometry was assessed by using ImageJ and used to evaluate the BAX/BCL2 ratio in treated cells vs. control cultures (Ctr). The results were analyzed by one-way ANOVA, followed by Dunnet's multiple comparisons test; *p = 0.01 (BxPC-3) and **p= 0.007 (HPAC) indicate a statistically significant increase compared to the control cultures.

3D Spheroids Cell Viability

Cell viability for 3D cell cultures was assessed via MTT [3-(4, 5-dimethylthiazol-2-yl)-2,5-diphenyltetrazolium bromide] assay as described in Masi M. *et al.* [13] and adapting the protocol reported in Bresciani G. *et al.*[14] BxPC-3 cells (3 x 10^5 cells/well) were seeded in sterile 1% agarose in 1X PBS-coated 96-multiwell plates, grown for 96 h to allow spheroids formation and treated for 144 h as previously indicated. After treatment, a sterile solution of 1 mg/mL MTT in 1X PBS was added to each well at the final concentration of 0.1 mg/mL. Plates were incubated at 37 °C for 4 h and formazan crystals were solubilized overnight by adding a 1:1 volume of SDS 10%/0.01M HCl solution to each well. Absorbance was measured at 570 nm and 690 nm wavelengths on a Tecan Spark® multiplate reader. Data were analyzed by two-way ANOVA with Tukey's post-test. Results reported in Figure 6 are shown as mean values ± SD (n = 5); combination vs ola alone (*p < 0.05, **p < 0.01) or vs **19** alone (#p < 0.05, ##p < 0.01, ###p < 0.001).

3D Spheroids Cell Death Assessment with Vital Dyes

For cell death assessment with vital dyes in 3D spheroids, BxPC-3 spheroids obtained as previously described were treated as detailed above. After treatment, Calcein-AM and PI were added to each well at the final concentration of 2.5 and 3.75 µM respectively as described in Baumann K.W. *et al.*[15] To determine cell death rate, Calcein-AM (Ex = 485 nm, Em = 530 nm) and PI (Ex = 535 nm, Em = 620 nm) fluorescence signals were measured on a Tecan Spark® multiplate reader and results were expressed as PI/Calcein-AM ratio.

Fluorescence and Brightfield Imaging

Fluorescence images were taken using a Leica DFC360 FX camera through a Leica DMI6000 B inverted microscope (Leica Microsystems, Wetzlar, Germany) equipped with filters for FITC, TRITC and DAPI (scale bars were added using the ImageJ software). Brightfield imaging of 3D spheroids was performed at 0, 48, 96 and 144 h treatment timings to determine spheroid volume change. Pictures were taken using a Leica DFC360 FX camera through a Leica DMI6000 B inverted microscope (scale bars were added using the ImageJ software). Image processing was performed with ImageJ software using the SA_NJ macro described

in Nunes J.S.P. *et al*.[16] Spheroid volume was calculated using the general formula described in Chen W. *et al* [17]: Spheroid Volume (V)= 0.5 ·(Length)·$(\text{Width})^2$

Statistical Analysis

All data were analyzed using the GraphPad Prism software, version 10 (GraphPad Software, San Diego, CA, USA) and outliers were determined with GraphPad outlier calculator (alpha = 0.05). All results were obtained from at least three independent experiments performed with duplicate or triplicate samples (exact number of replicates is indicated in the respective figure legend). The results are expressed as mean values ± standard deviation (SD) and were calculated using all the data obtained from the independent experiments; the significance level was set at $**p < 0.05$.

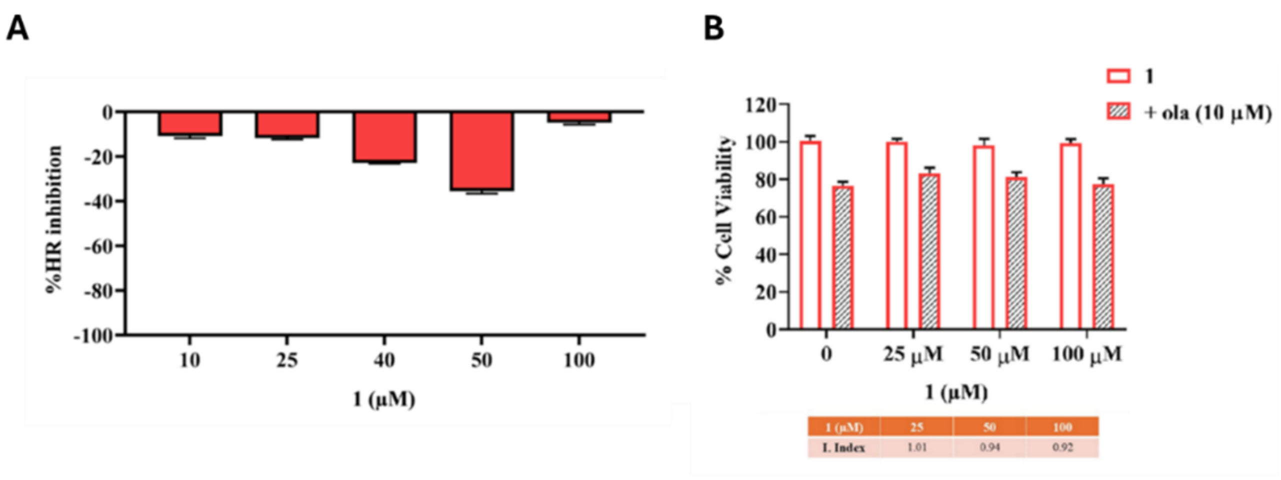

Figure S2. HR inhibition of **1** and cell viability of **1**/ola combination

A) HR inhibition assay of **1**. Compound **1** was tested from 10 µM to 100 µM. It reached an HR inhibition of 35 % at 50 µM, which dropped to 4 % at 100 µM; **B**) Evaluation of **1**/ola combination in BxPC-3 cells, and I. Index. The **1**/ola 144 h combination did not show a statistically significant decrease compared to treatment with ola alone (Data were statistically analyzed using the two-way ANOVA followed by Tukey's multiple comparisons test). The table below the graph indicates an additive effect when **1** (25-50-100 µM) is combined with ola.

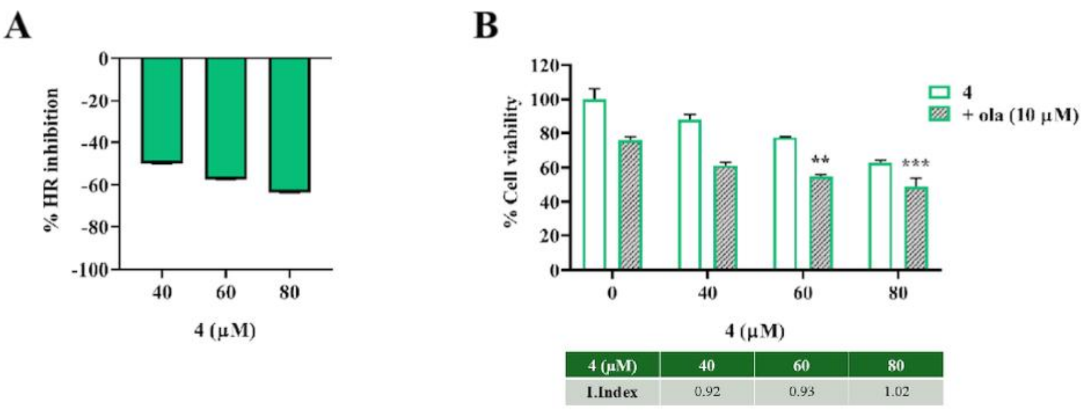

Figure S3. HR inhibition of **4** and cell viability of **4**/ola combination

Preliminary biological evaluation of compound **4**. **A**) Graph of the HR inhibition % reached by compound **4** (maximum 63 % at 80 µM). **B**) Graph of the effect of **4**/olaparib combination on BxPC-3 cells after 144 h of treatment. Although the **4** (60-80 µM) / olaparib combination decreases cell viability in a statistically significant manner compared to cells treated with olaparib alone (data were statistically analyzed using the two-way ANOVA followed by Tukey's multiple comparisons test), the I. Index > 0.8 indicated an additive combination effect.

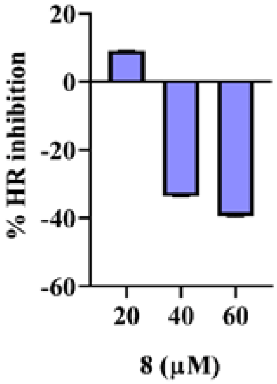

Figure S4. HR inhibition of **8**

HR inhibition assay of **8**. Compound **8** was tested at concentrations of 20, 40, and 60 µM, based on its EC_{50} value of 24 ± 6 µM determined from the ELISA assay. Even at the highest concentration (60 µM), **8** achieved only 39% inhibition of HR, indicating that it is a relatively weak HR inhibitor.

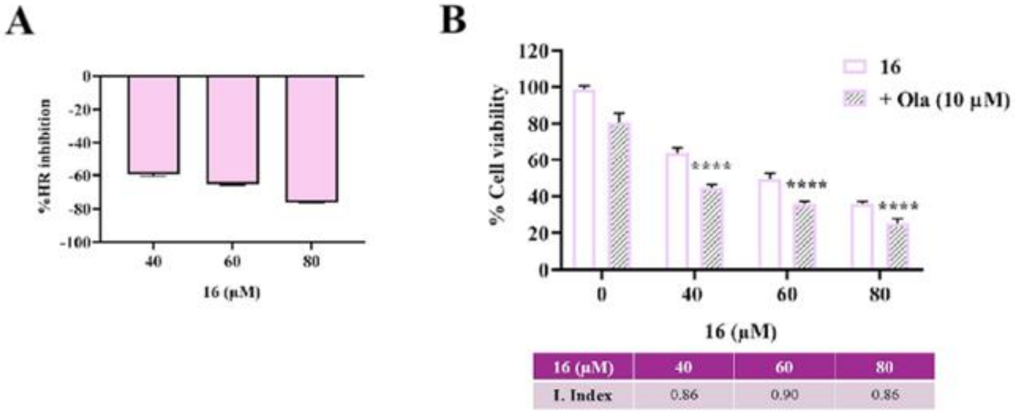

Figure S5. HR inhibition of **16** and cell viability of **16**/ola combination

Preliminary biological evaluation of compound **16**. **A**) Graph showing the percentage of HR inhibition achieved by compound **16**, with a maximum of 76% observed at 80 µM. **16** was tested at 40-60-80 µM, considering its EC_{50} in ELISA (51.9 ± 6.4 µM*)*. **B**) Graph of the effect of the **16**/olaparib combination on BxPC-3 cell viability after 144 h of treatment. Although the combination of compound **16** (40-60-80 µM) and olaparib significantly reduced cell viability compared to treatment with olaparib alone (statistically analyzed using two-way ANOVA followed by Tukey's multiple comparisons test), the I. Index > 0.8) suggests an additive rather than a synergistic effect. Moreover, **16** alone has a marked effect on cell viability (40 % cell viability reduction at 40 µM, which is the lower dose tested).

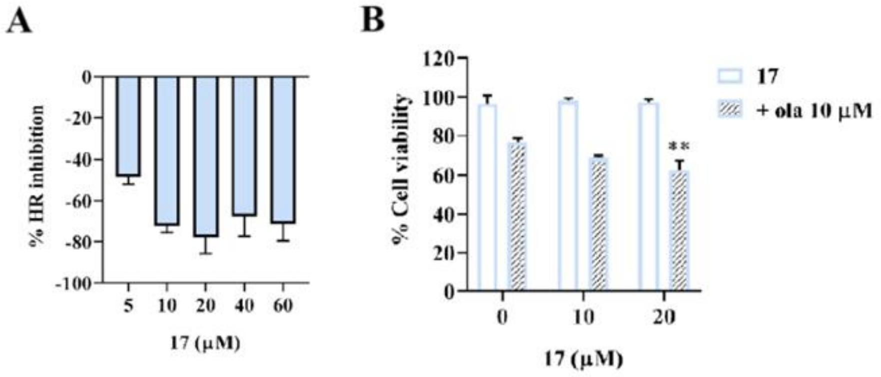

17 (µM)	10	20
I. Index	0.91	0.84

Figure S6. HR inhibition of **17** and cell viability of **17**/ola combination

Preliminary biological evaluation of compound **17**. **A**) Graph showing the percentage of HR inhibition achieved by compound **17** (ELISA EC_{50} = 35.5 ± 2.5), tested from 5 µM up to 60 µM with a maximum of 77% observed at 20 µM. **B**) Graph of the effect of the **17**/olaparib combination on BxPC-3 cell viability after 144 h of treatment. We observed a statistically significant decrease of cell viability due to the compounds' combination compared to the single treatment with Olaparib at **17** 20 µM (two-way ANOVA followed by Tukey's multiple comparisons test). However, the I. Index > 0.8 suggests an additive rather than a synergistic effect.

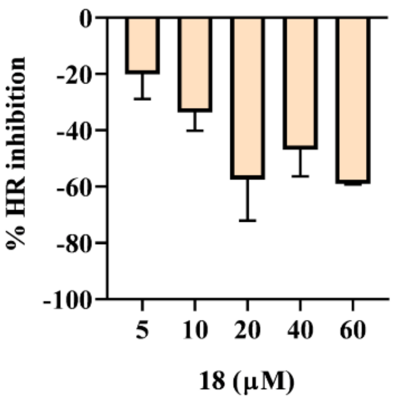

Figure S7. HR inhibition of **18**

HR inhibition assay of **18**. Compound **18** was tested at 5-10-20-40 and 60 µM (ELISA EC_{50} = 3.8 ± 0.1 µM). It did not exceed 60 % of HR inhibition, observed at 20 µM. Moreover, it showed a drop at 40 µM and 60 µM. It did not show a dose-dependent HR inhibition, suggesting eventual in-cell off-target effects.

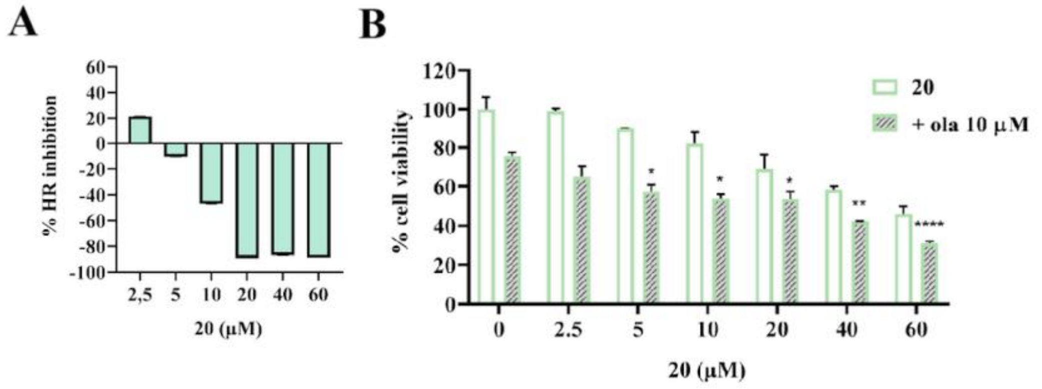

20 (μM)	2.5	5	10	20	40	60
I. Index	0.87	0.85	0.87	1.03	0.96	0.89

Figure S8. HR inhibition of **20** and cell viability of **20**/ola combination

Preliminary biological evaluation of compound **20**. **A**) Graph showing the percentage of HR inhibition achieved by compound **20**, tested from 2.5 μM up to 60 μM with a maximum of 89% observed at 20 μM (ELISA EC_{50} = 43.8 ± 6.8 μM). **B**) Graph of the effect of the **20**/olaparib combination on BxPC-3 cell viability after 144 h of treatment. We observed a statistically significant decrease in cell viability due to the compounds' combination compared to olaparib alone at all **20** doses except 2.5 μM (two-way ANOVA followed by Tukey's multiple comparisons test). However, the I. Index > 0.8 suggests an additive rather than a synergistic effect. Furthermore, the limited effect on HR at 5 μM cannot account for the cell viability reduction of ≈ 40 % observed in combination with olaparib, an event that strongly suggested potential off-target effects.

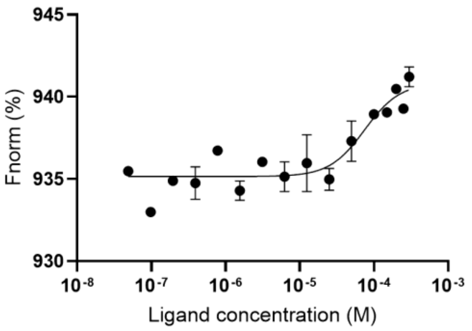

Figure S9. MST analysis of **19** binding to RAD51

MST analysis of the RAD51-**19** interaction (n = 3). Fluorescently labelled RAD51 was titrated with increasing concentrations of compound **19** leading to a binding curve with an estimated affinity constant (K_d) of K_d ≈ 75.14 ± 0,06 μM for the binding even.

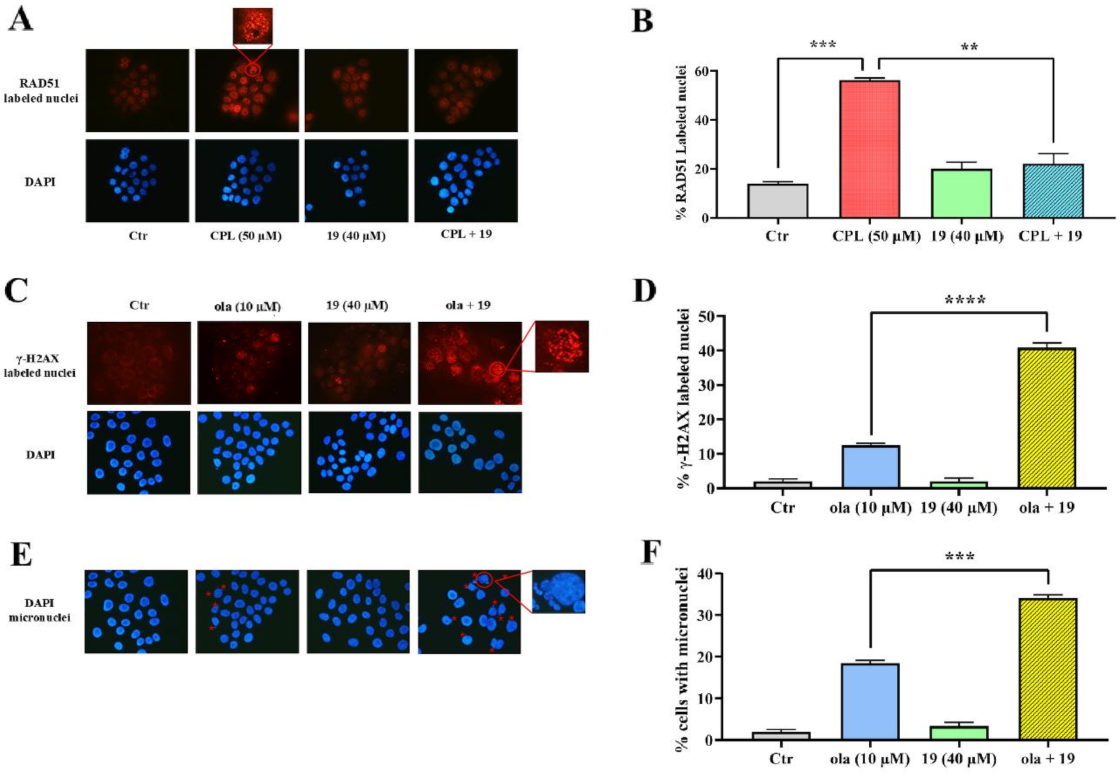

Figure S10. Evaluation of RAD51 foci, γ-H2AX nuclei and micronuclei formation.

A) Representative images show RAD51 immunodetection co-localized with DAPI in BxPC-3 cell nuclei, with an enlarged view of RAD51 foci in CPL-exposed cultures; **B**) RAD51-labelled nuclei percentage in different samples. Results are mean values ± SD (n = 3). RAD51 labelling was 56% in CPL-exposed cultures (***p = 0.0003 vs controls) and 22% in cultures exposed to both **19** and CPL (**p = 0.001 vs CPL); **C**) Immunofluorescence of γ-H2AX foci (red) in BxPC-3 cells after 72h exposure to **19** and ola, alone or in combination, with DAPI-stained nuclei. Higher magnification shows γ-H2AX foci in combination-treated cultures; **D**) Percentage of γ-H2AX-labelled nuclei. In combination-exposed cultures, γ-H2AX labelling was >40% (****p < 0.0001 vs ola alone); **E**) DAPI-stained micronuclei in the cytoplasm of cells exposed to **19**/ola for 72h (asterisks indicate micronuclei); **F**) Percentage of micronuclei-bearing cells. Results are mean values ± SD (n = 3). **19** + ola significantly increased micronuclei formation compared to ola alone (***p = 0.0001).

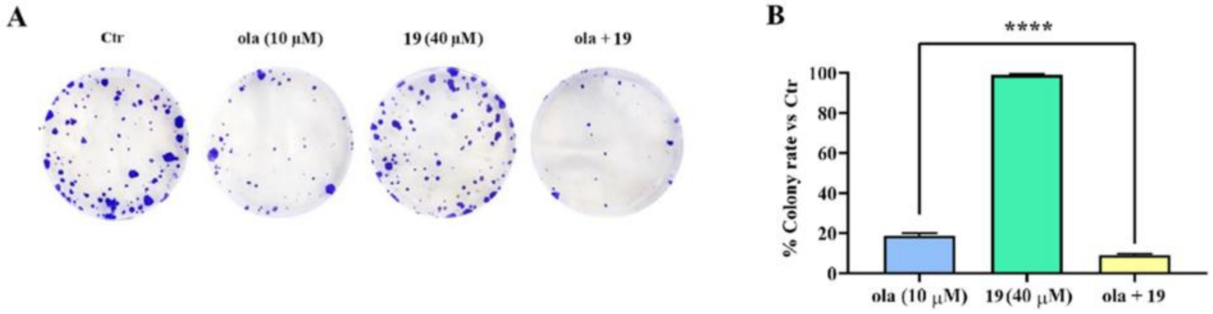

Figure S11. Evaluation of colony formation of **19**/ola combination

A) Representative images of formalin-fixed, crystal violet stained colonies; **B**) Statistically significant reduction after ola/**19** treatment, compared to cells exposed to the single ola treatment (**** $p < 0.0001$). Results expressed as mean ± SD (n = 3).

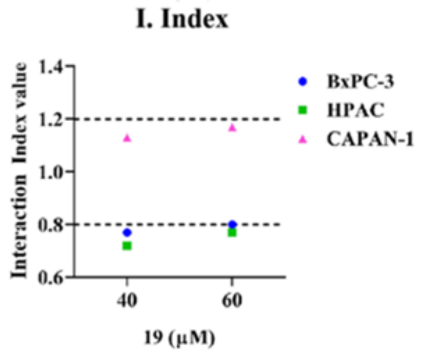

Figure S12. Interaction Index of **19**/ola combination in BxPC-3, HPAC and CAPAN-1

Interaction Index (I. Index) values after 6-day treatment with the combination ola + **19** on BxPC-3 (blue), HPAC (green), and CAPAN-1 (violet). I. Index values indicate synergy (<0.8), additivity (0.8–1.2), or antagonism (>1.2). BxPC-3: 0.77 (40 µM), 0.80 (60 µM); HPAC: 0.72 (40 µM), 0.77 (60 µM); CAPAN-1: 1.13 (40 µM), 1.17 (60 µM).

Table S2. Comparative analysis of **19** with **35d** and **CAM833**

Name	PPI binding site	Kinetic solubility (μM) [a]	ELISA EC_{50} (μM) [b]	% HR inhibition (mCl-HR) [b]	Combination w/ola in BxPC-3 cells [b]	I. Index [c]
**19** (ARN26912)	LFDE pocket (Zone II)	>250	29.35 ± 2.25	74% at 40 μM	Synergism	0.77
**35d** (ARN24089)	LFDE pocket (Zone II)	<1	20 ± 1[1, 18]	67% at 40μM[18]	Synergism	0.54
**CAM833**	FxxA pocket (Zone I)	171 ± 5	10 ± 2	>90% at 50 μM[4,*]	Additive/Synergism (Figure S15) **	0.79

[a] Section S.3
[b] Section S.4
[c] Reported I. Index refer to 40 μM of the tested compound in combination with 10 μM ola
[e] Section S.4.1.10
* Experiment performed in HeLa Kyoto cells and reported in Scott DE *et al*[4]
** Synergism was reported by Scott DE *et al*[4] in combination studies of 20 μM CAM833 with 1μM PARP1 inhibitor, AZD2461, in HCT116 cells.[4]

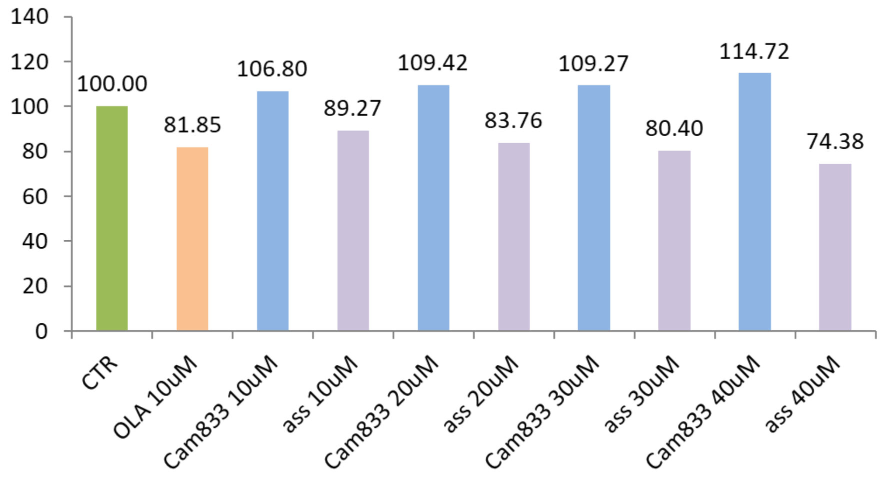

Figure S13. Cell viability of **CAM833**, ola, or combination in BxPC-3 cell

Cell viability of 6-day treatment; I. Index calculated for the combination of scalar doses of **CAM833** and 10 μM fixed concentration of ola: 1.02 (10 μM), 0.93 (20 μM); 0.89 (30 μM), 0.79 (40 μM).

S4. ^{1}H-NMR spectrum, ^{13}C-NMR spectrum and UPLC-MS analysis of final compounds **1-20**

^{1}H-NMR spectrum (401 MHz, DMSO-d_6) of **1**

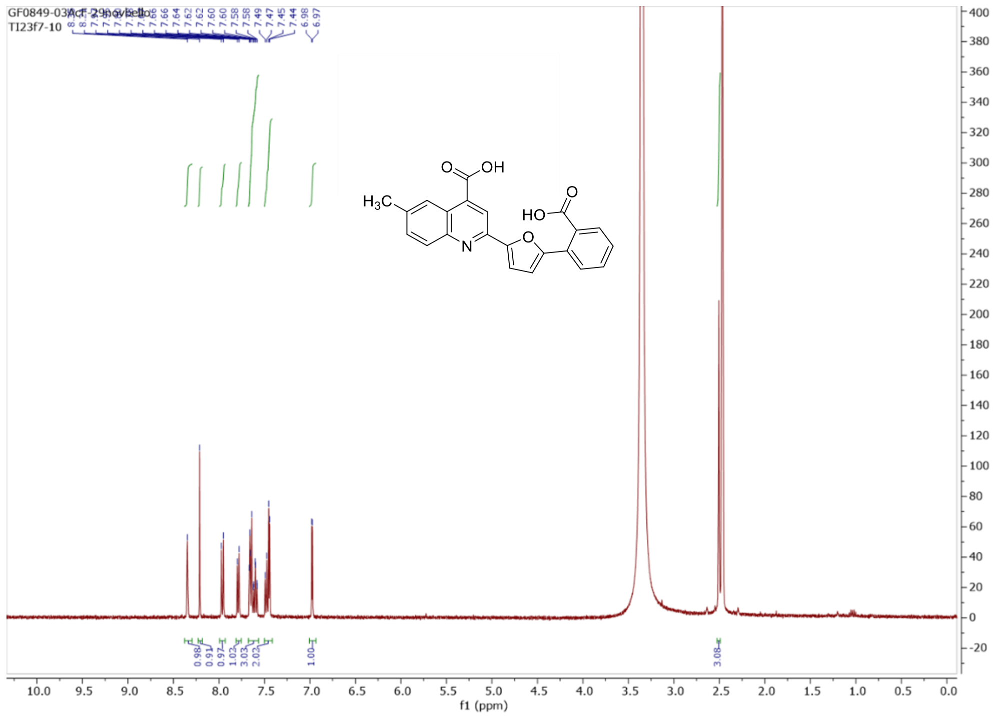

^{13}C-NMR spectrum (101 MHz, CD_3OD) of **1**

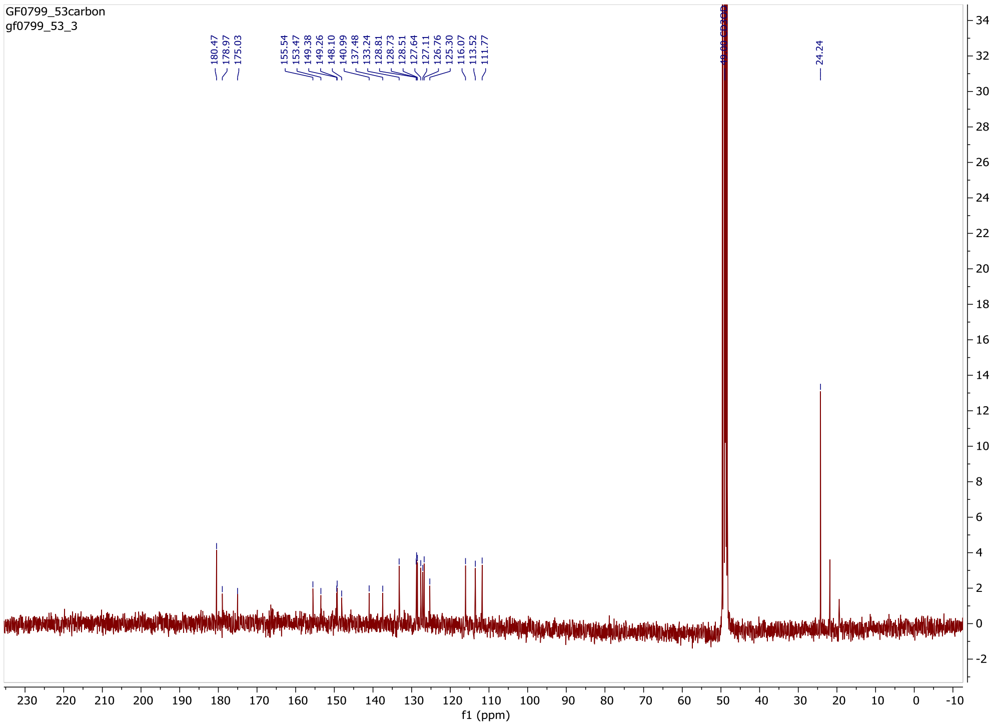

UHPLC-MS analysis of **1**

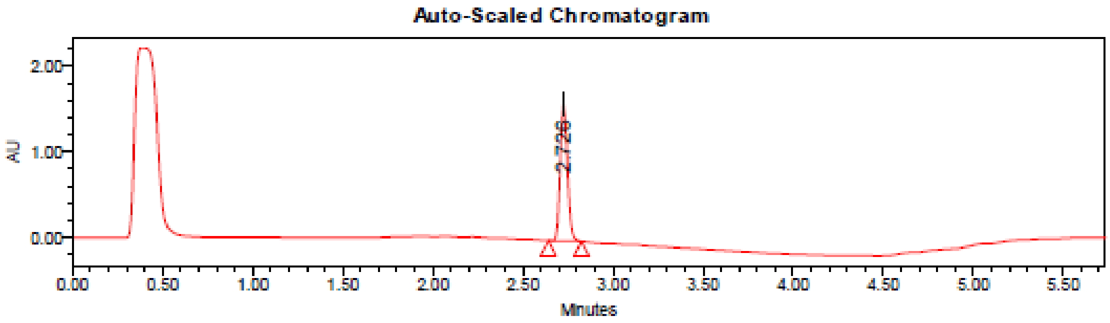

Processed Channel: W2489 ChA 215nm

	Processed Channel	Retention Time (min)	Area	% Area	Height
1	W2489 ChA 215nm	2.726	4475192	100.00	1594047

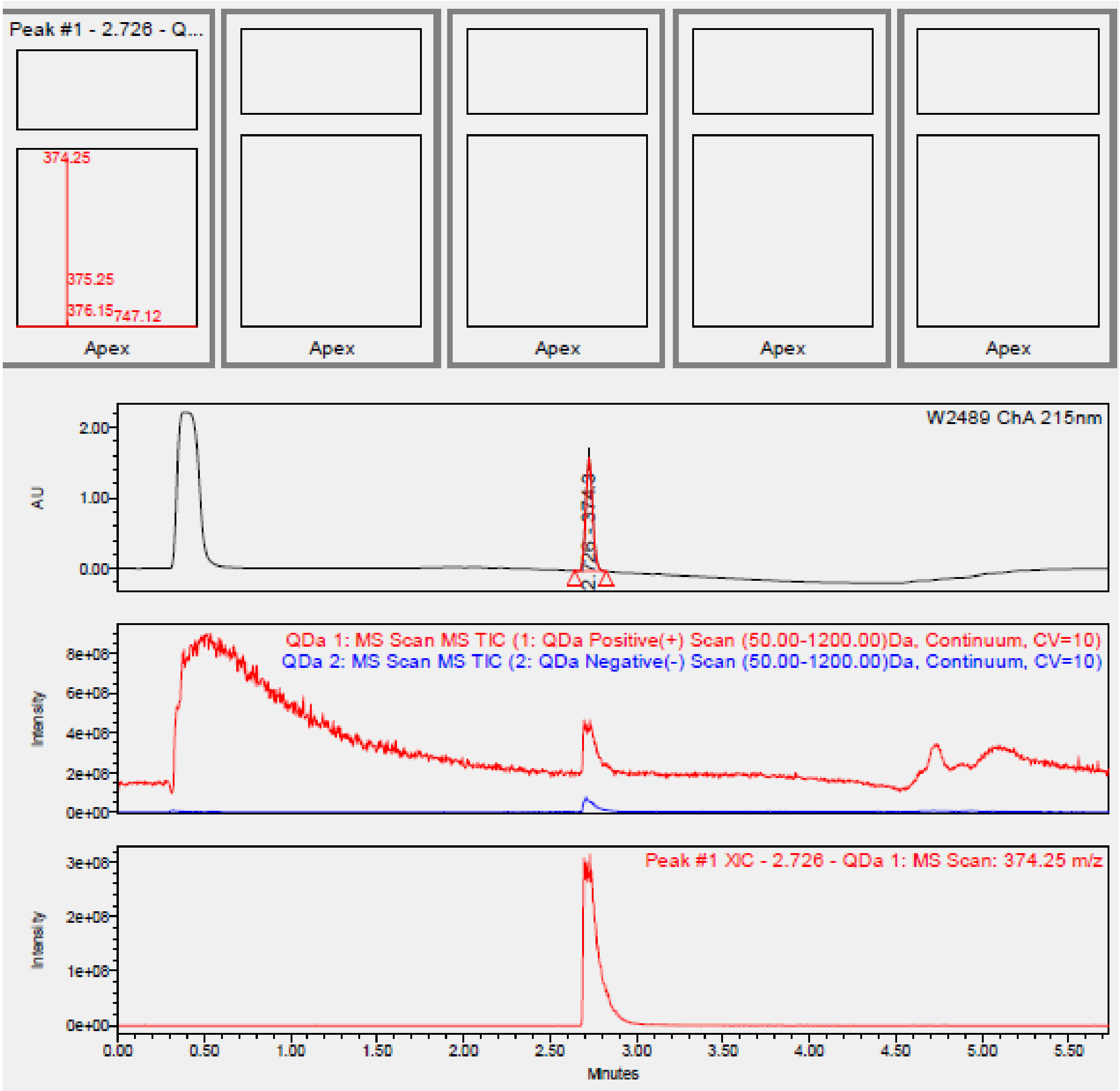

^{1}H-NMR spectrum (401 MHz, DMSO-d_6) of **2**

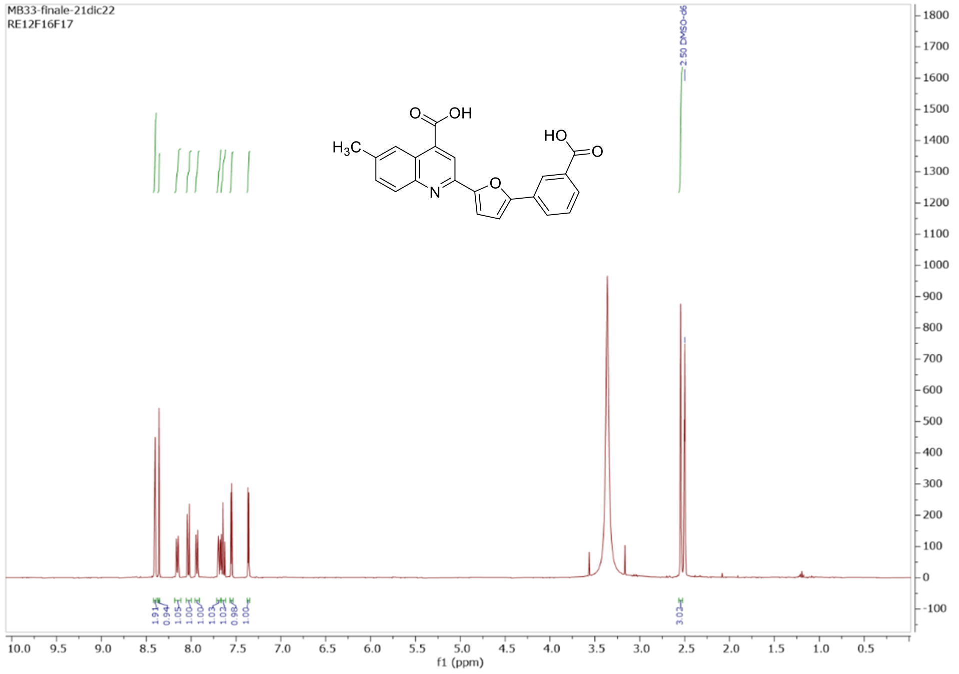

^{13}C-NMR spectrum (101 MHz, DMSO-d_6) of **2**

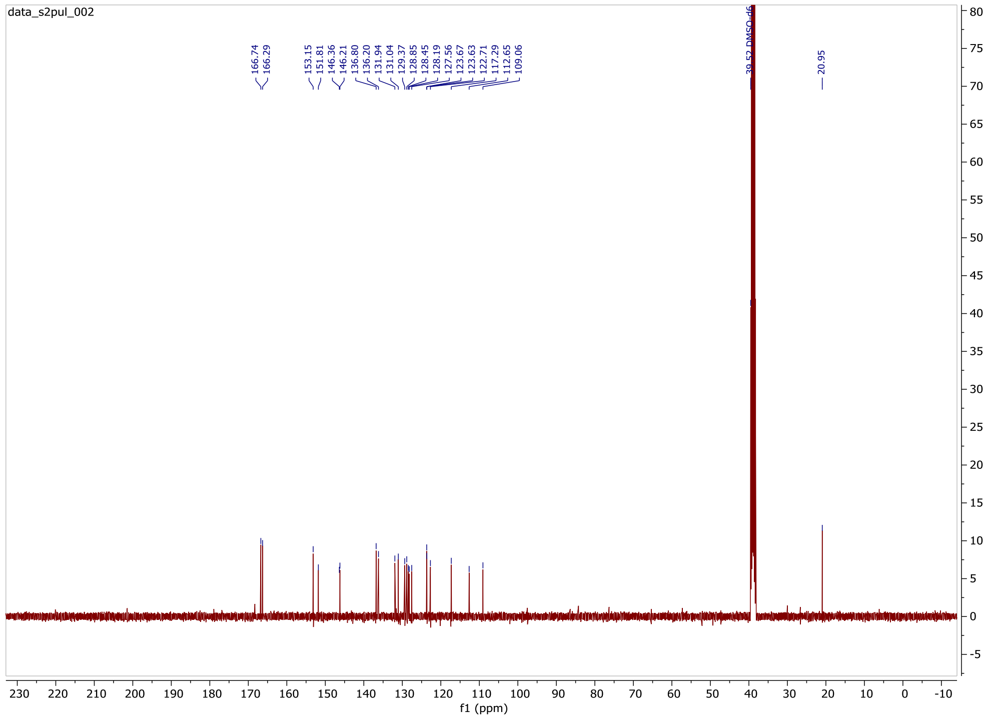

UHPLC-MS analysis of **2**

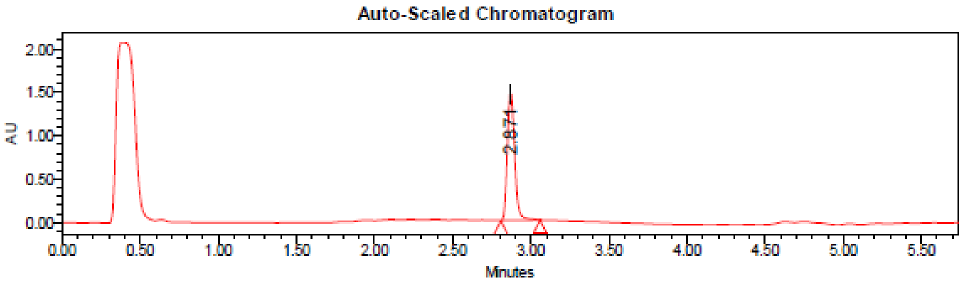

Processed Channel: W2489 ChA 215nm

	Processed Channel	Retention Time (min)	Area	% Area	Height
1	W2489 ChA 215nm	2.871	4752726	100.00	1475312

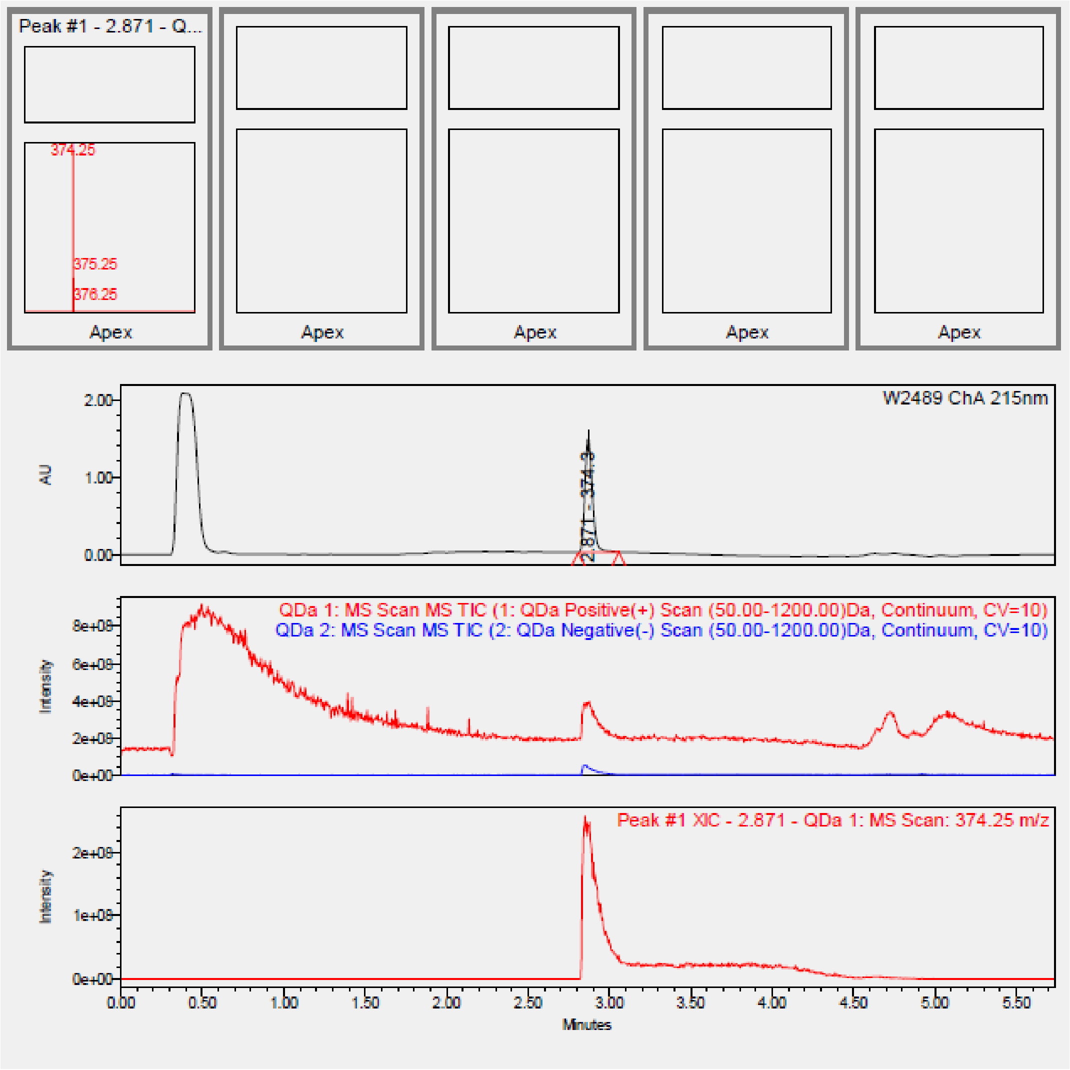

^{1}H-NMR spectrum (401 MHz, DMSO-d_6) of **3**

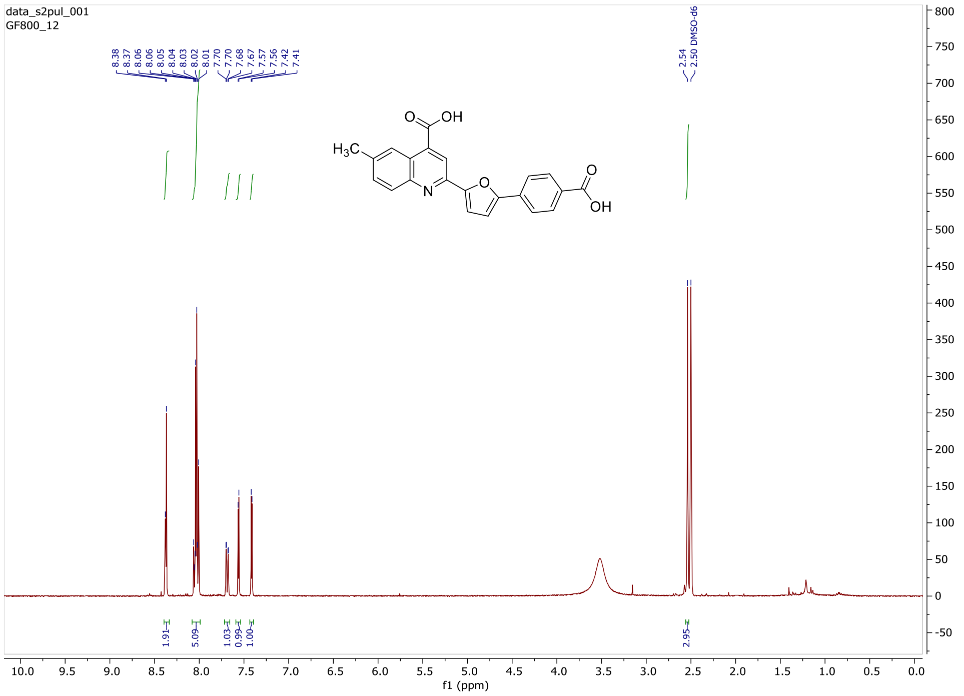

^{13}C-NMR spectrum (101 MHz, DMSO-d_6) of **3**

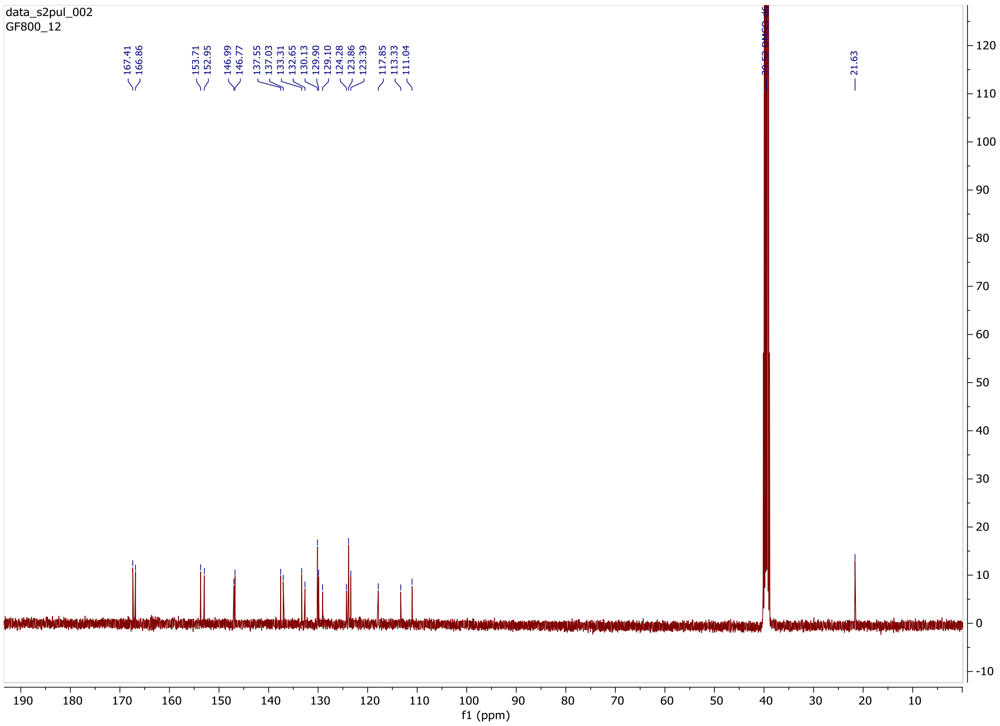

UHPLC-MS analysis of **3**

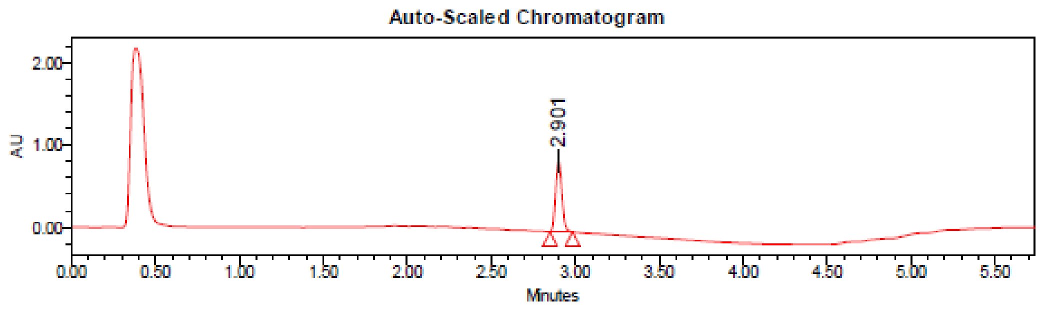

Processed Channel: W2489 ChA 215nm

	Processed Channel	Retention Time (min)	Area	% Area	Height
1	W2489 ChA 215nm	2.901	2199986	100.00	853095

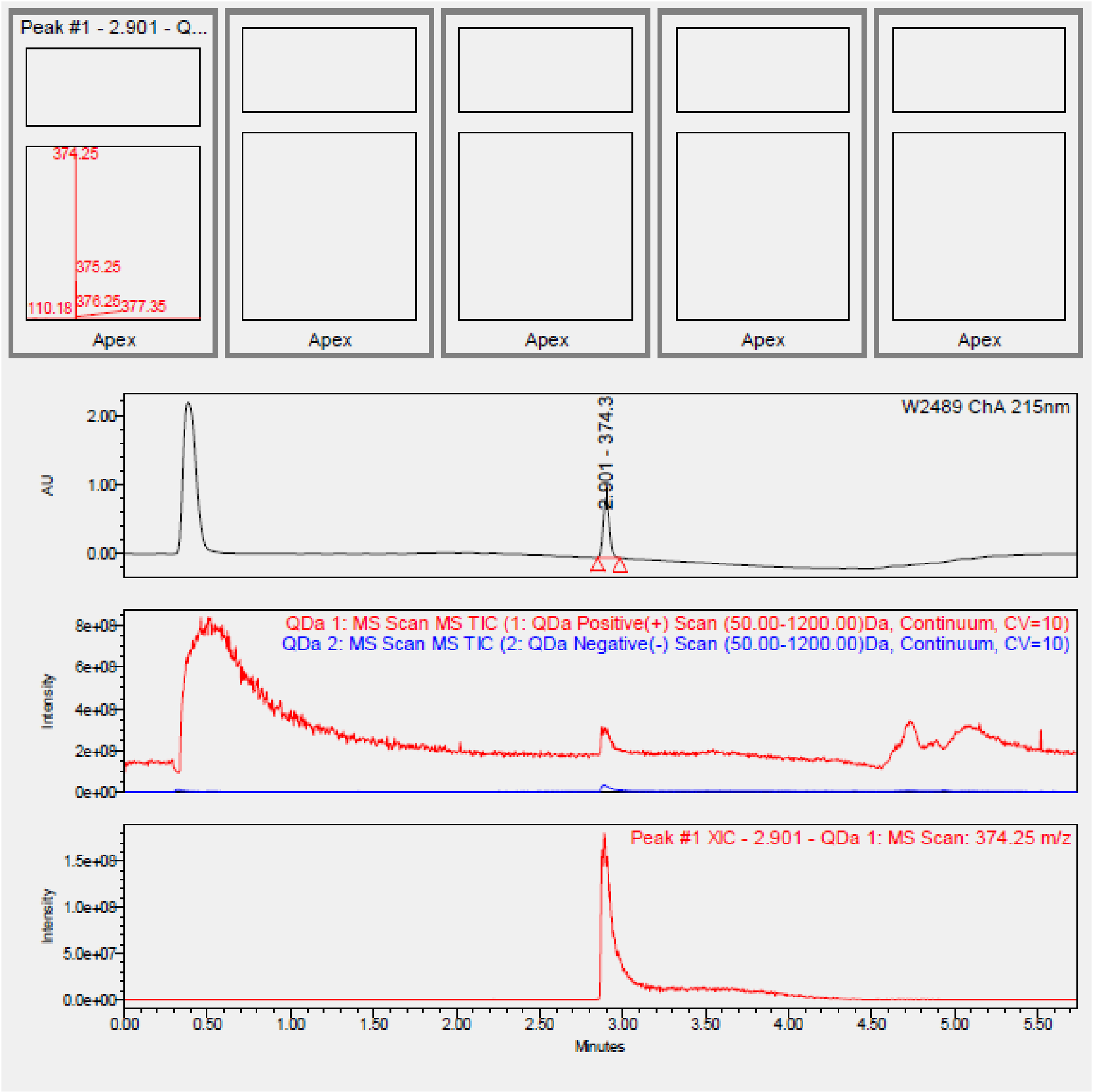

^{1}H-NMR spectrum (401 MHz, DMSO-d_6) of **4**

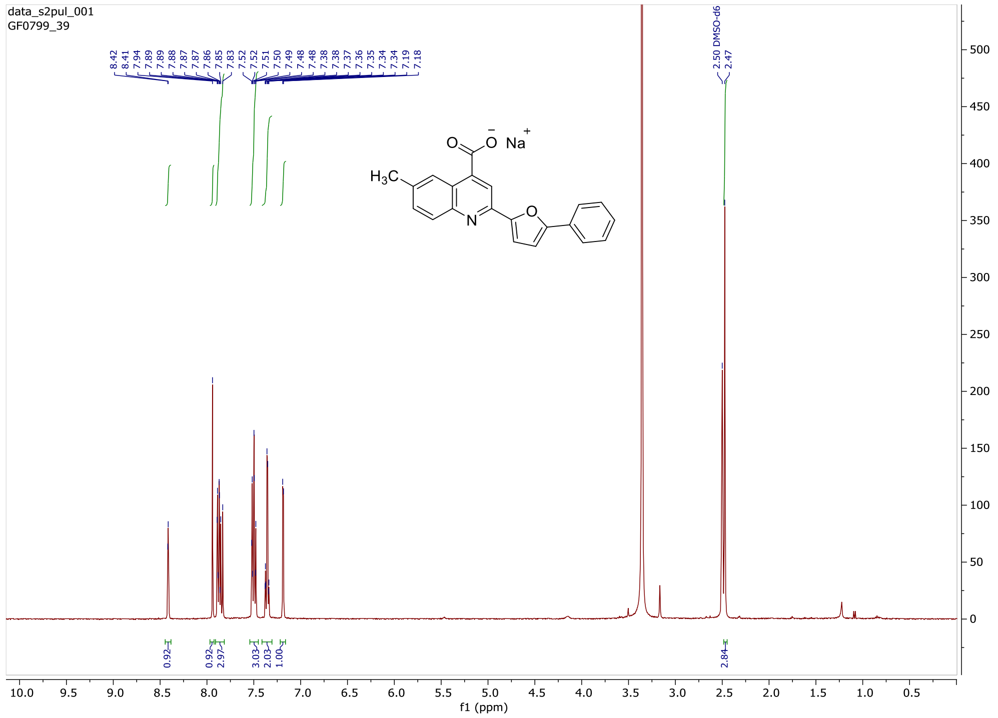

^{13}C-NMR spectrum (101 MHz, DMSO-d_6) of **4**

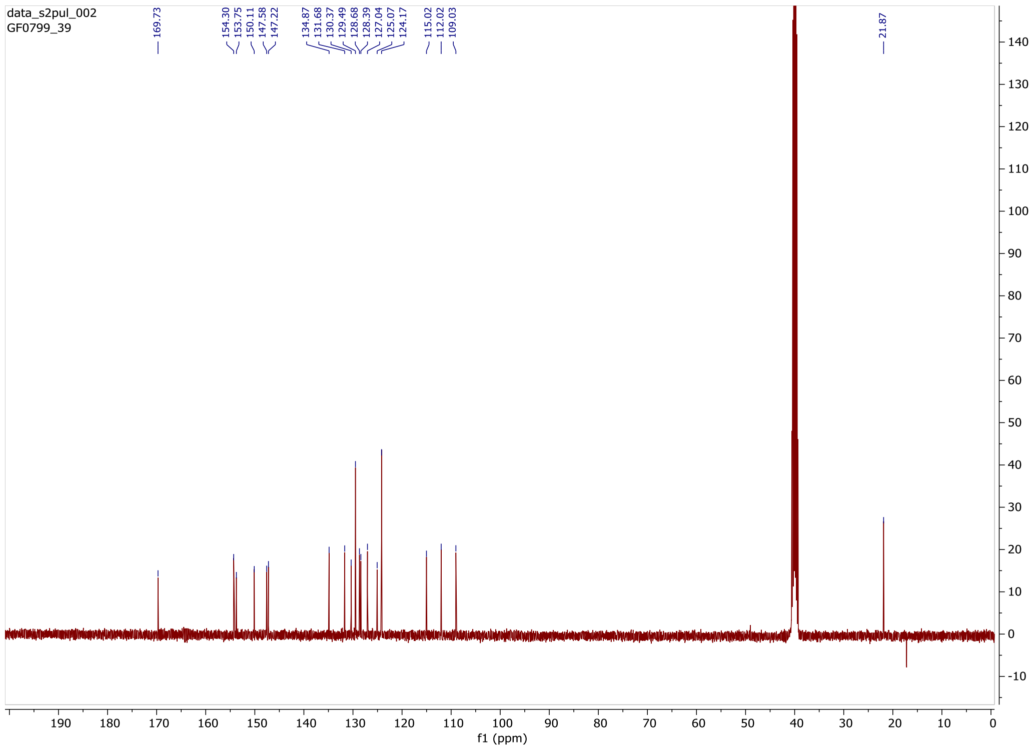

UHPLC-MS analysis of **4**

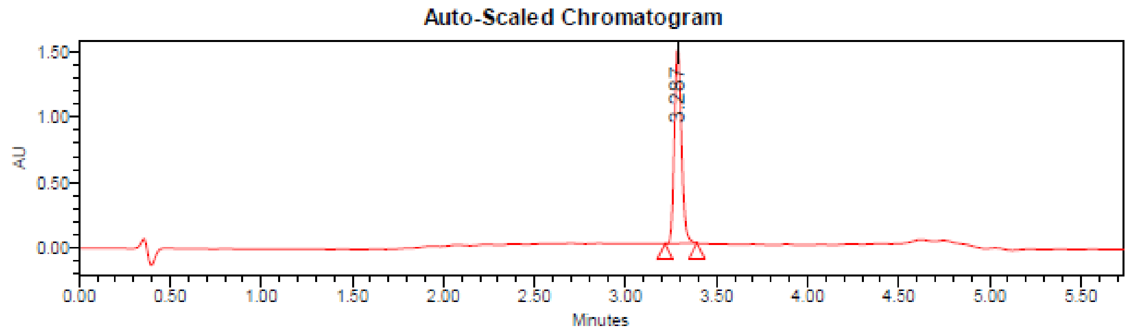

Unknown Peak Results

	Peak Type	RT	Area	% Area
1	Unknown	3.287	4029347	100.00

Unknown Peak Results

	Height
1	1479830

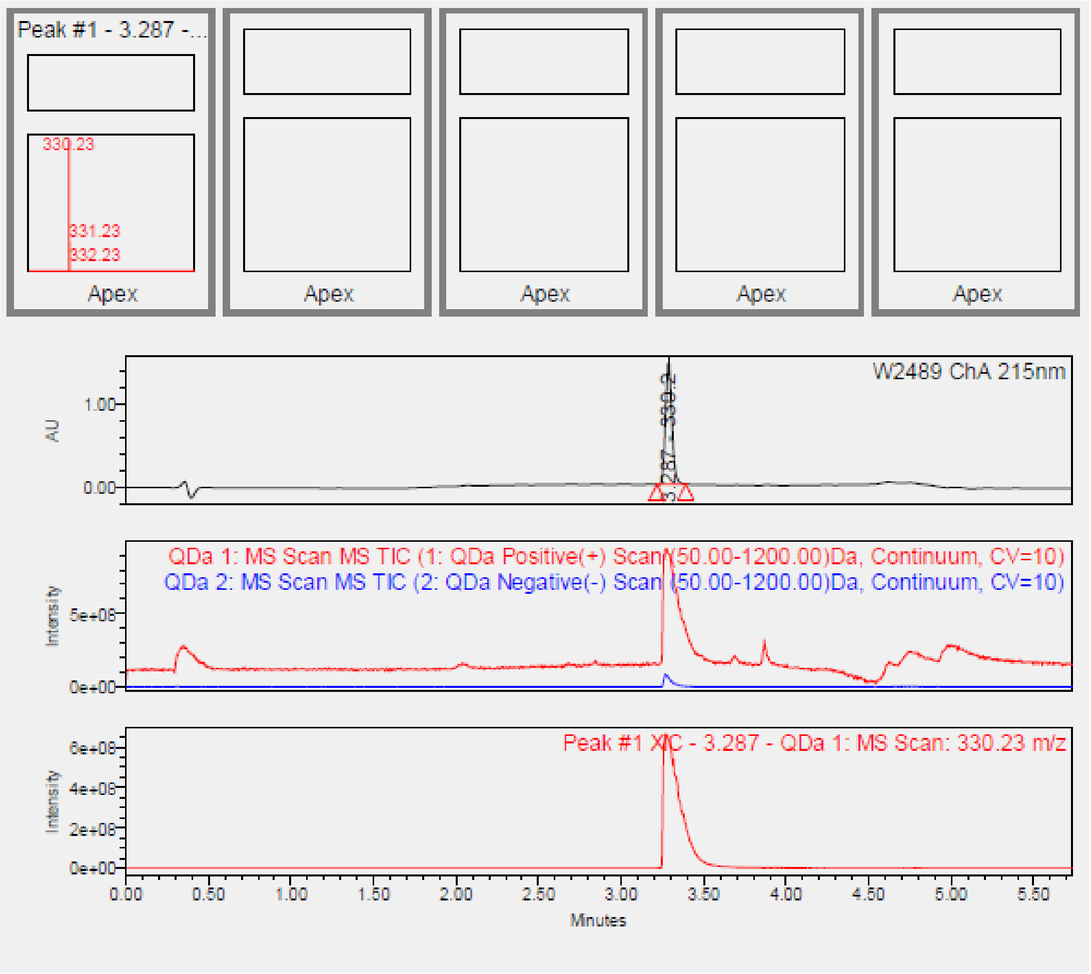

^{1}H-NMR spectrum (401 MHz, CD_3OD) of **5**

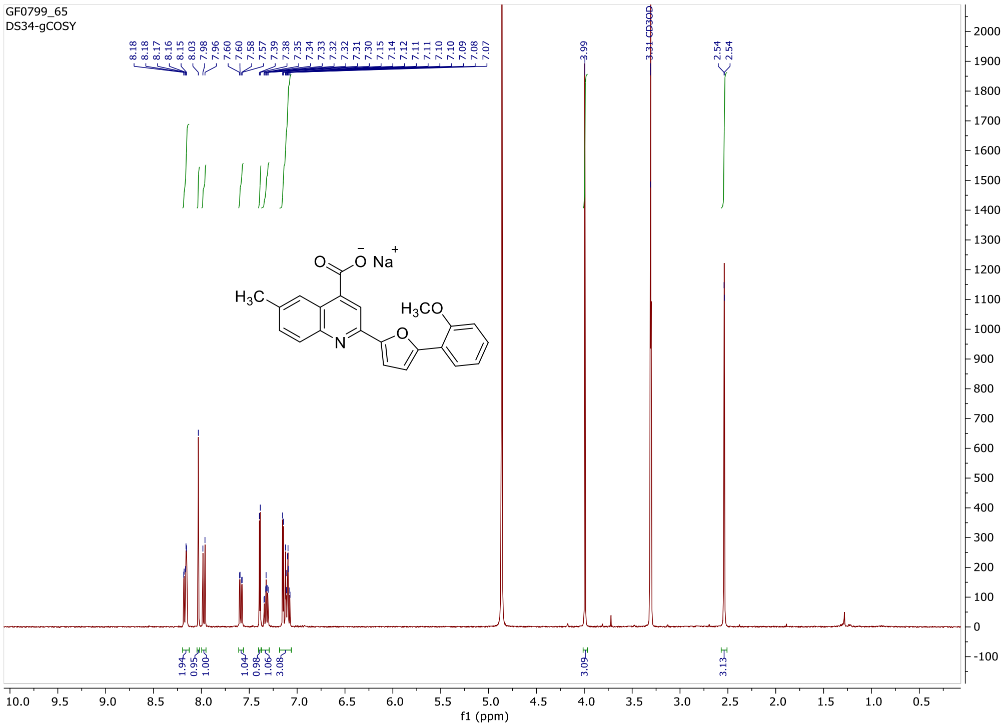

^{13}C-NMR spectrum (101 MHz, CD_3OD) of **5**

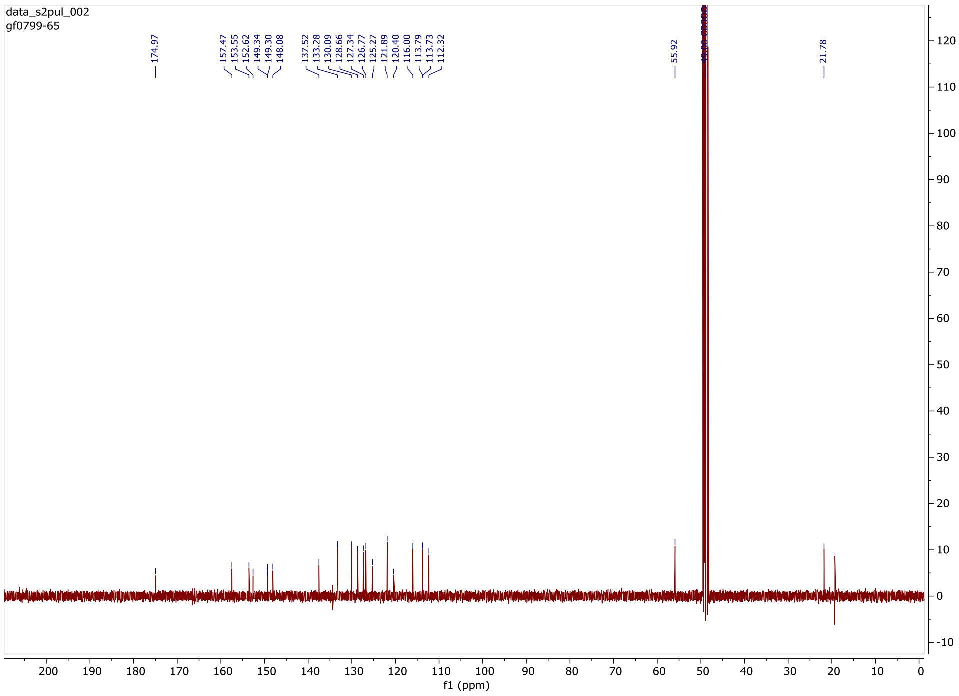

UHPLC-MS analysis of **5**

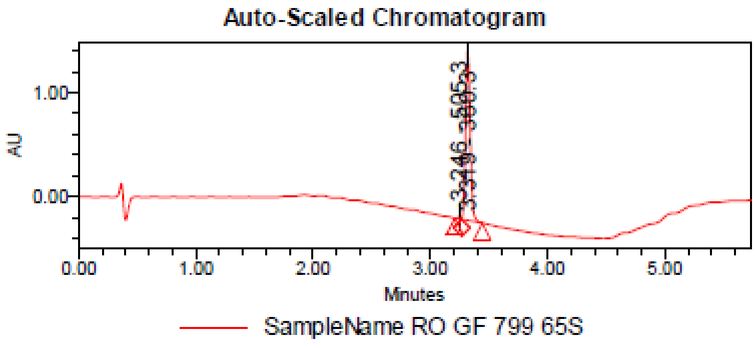

Peak Results

	Name	RT	Area	% Area	Height
1		3.246	32144	0.72	15367
2		3.319	4415105	99.28	1607457

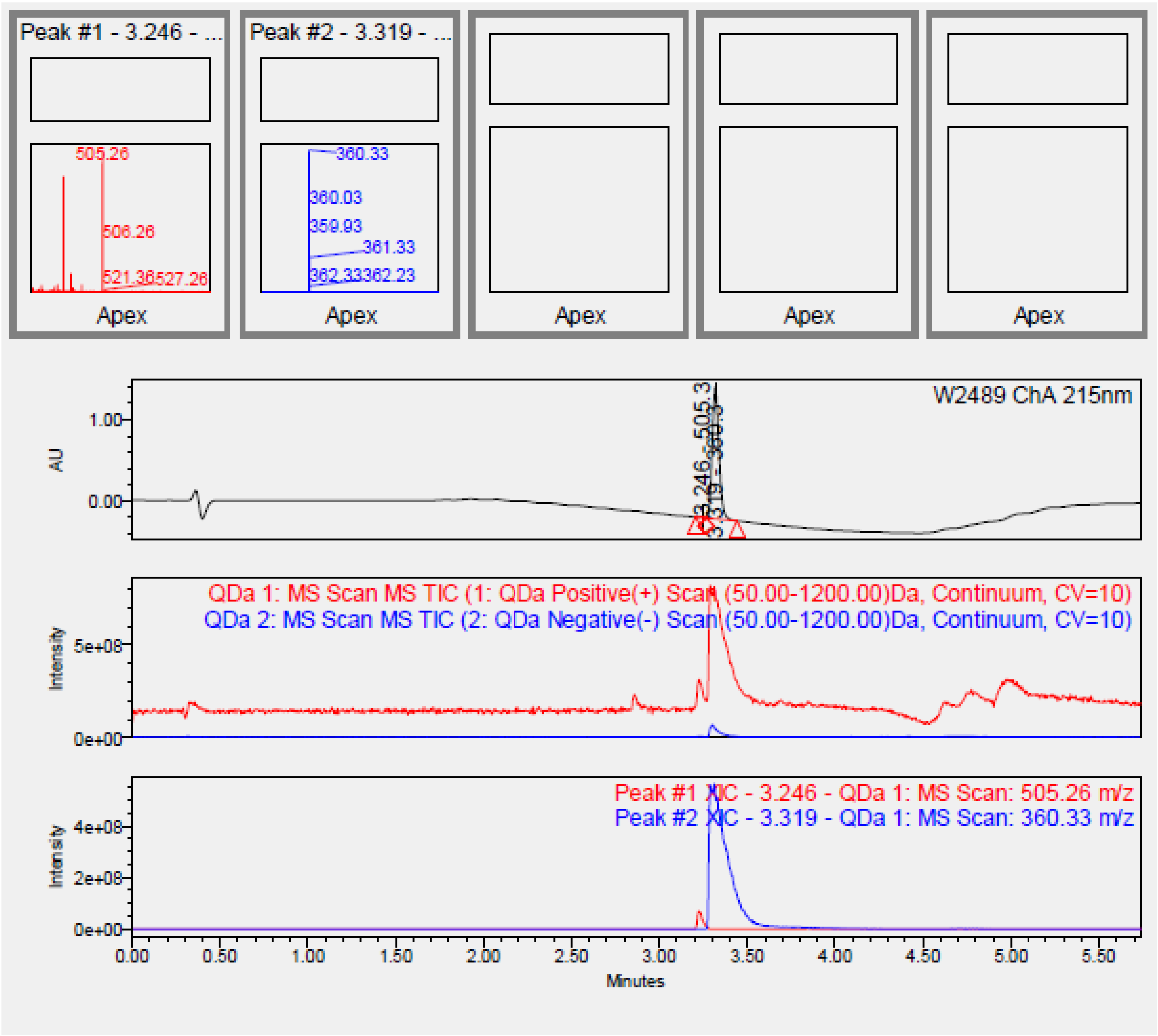

^{1}H-NMR spectrum (401 MHz, DMSO-d_6) of **6**

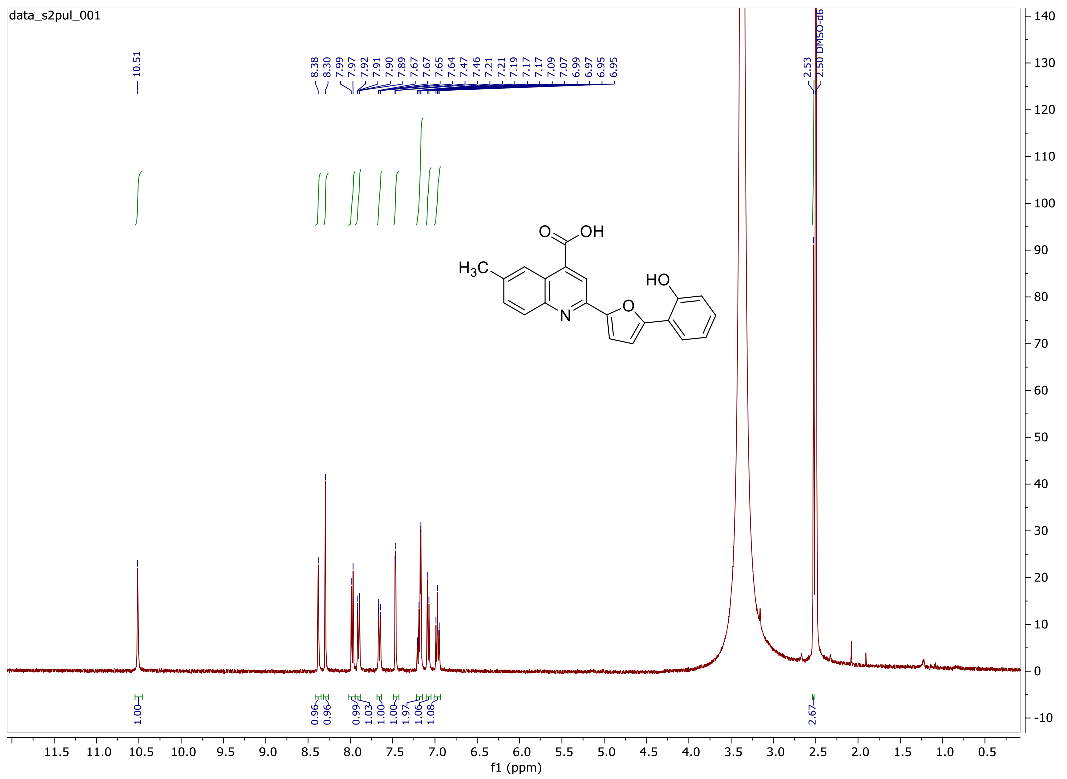

^{13}C-NMR spectrum (101 MHz, DMSO-d_6) of **6**

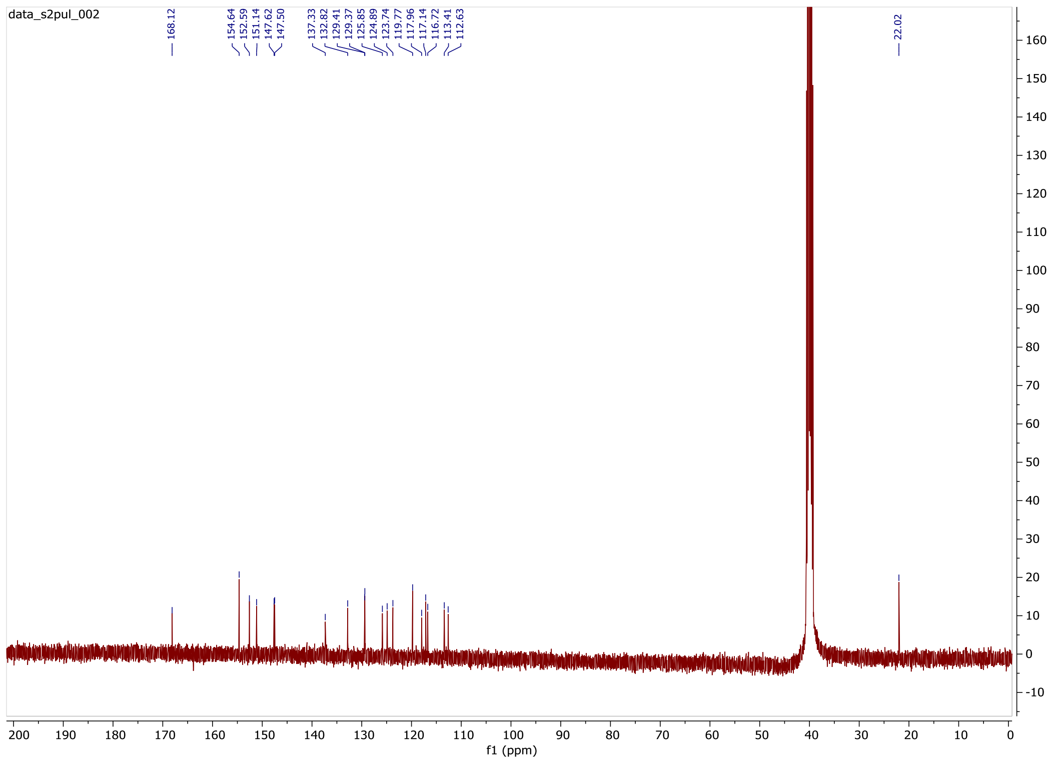

UHPLC-MS analysis of **6**

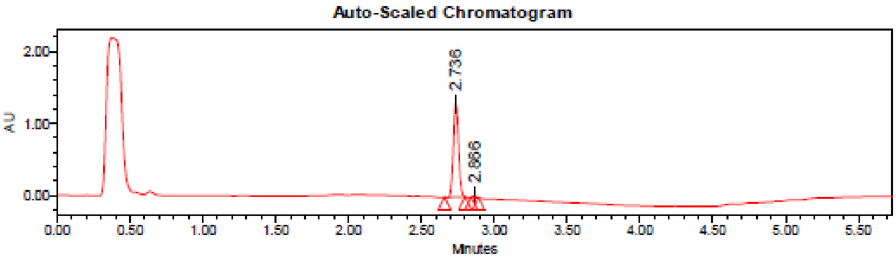

Processed Channel: W2489 ChA 215nm

	Processed Channel	Retention Time (min)	Area	% Area	Height
1	W2489 ChA 215nm	2.736	3246721	98.80	1305475
2	W2489 ChA 215nm	2.866	39345	1.20	22284

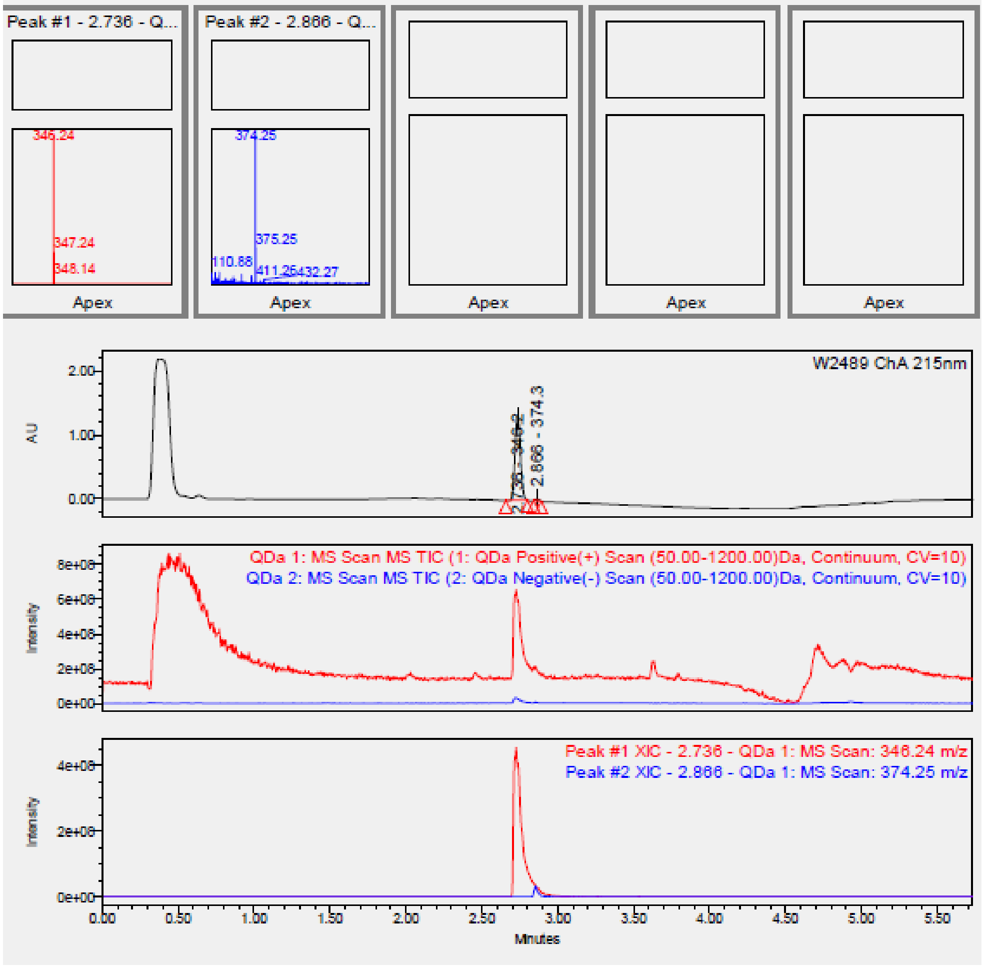

^{1}H-NMR spectrum (401 MHz, CD_3OD) of **7**

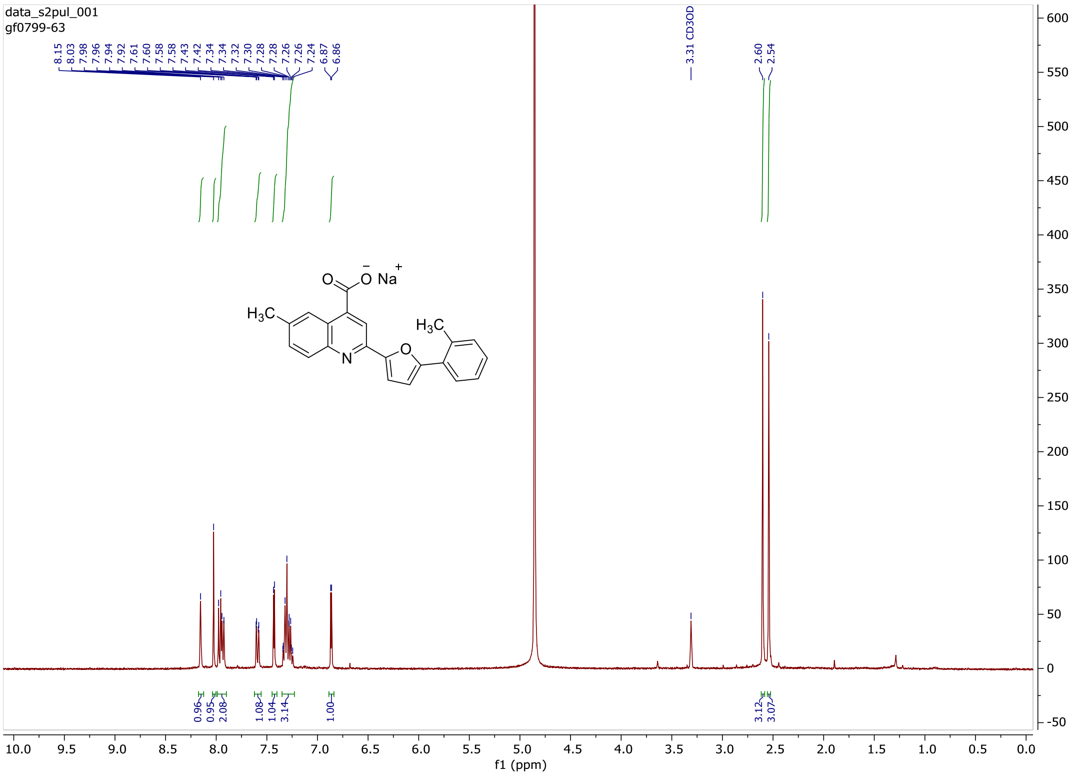

^{13}C-NMR spectrum (101 MHz, CD_3OD) of **7**

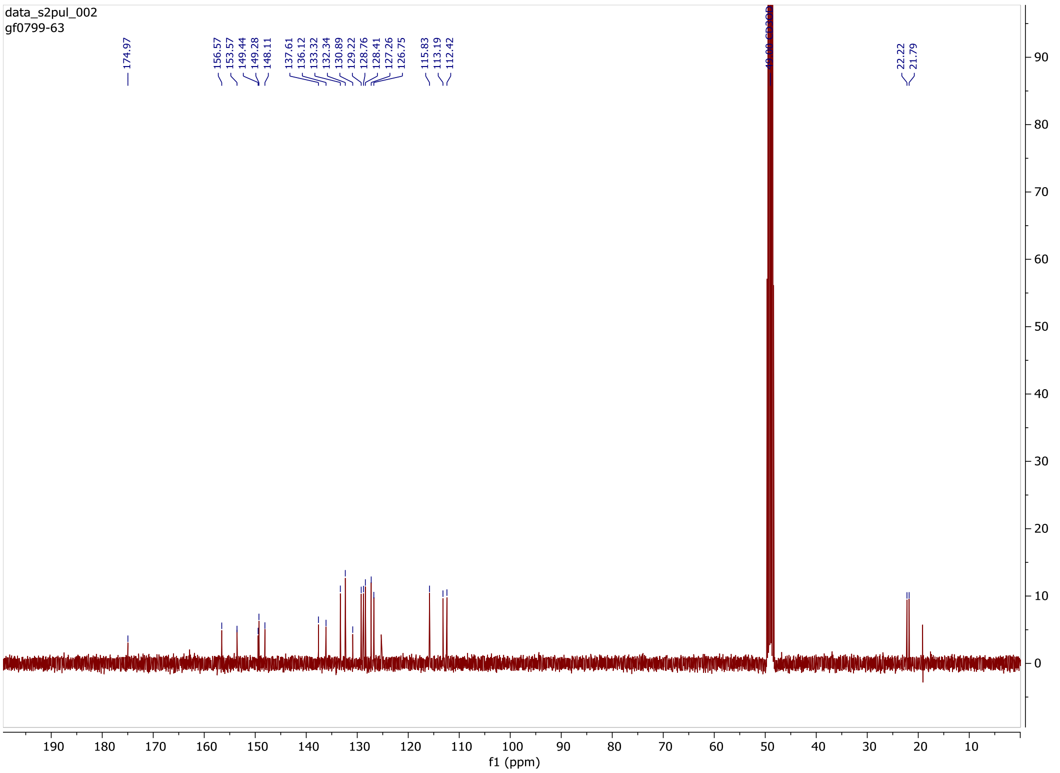

UHPLC-MS analysis of **7**

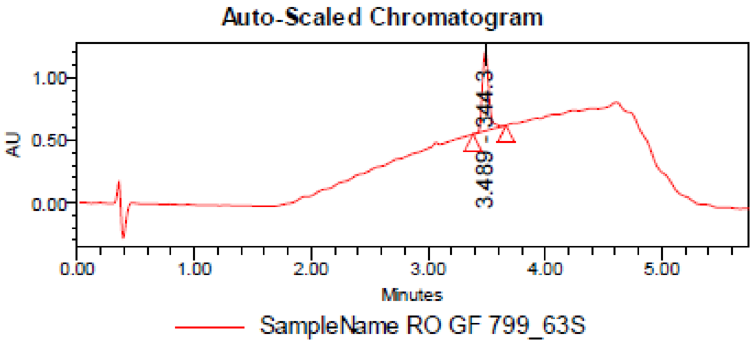

Peak Results

	Name	RT	Area	% Area	Height
1		3.489	2395538	100.00	631532

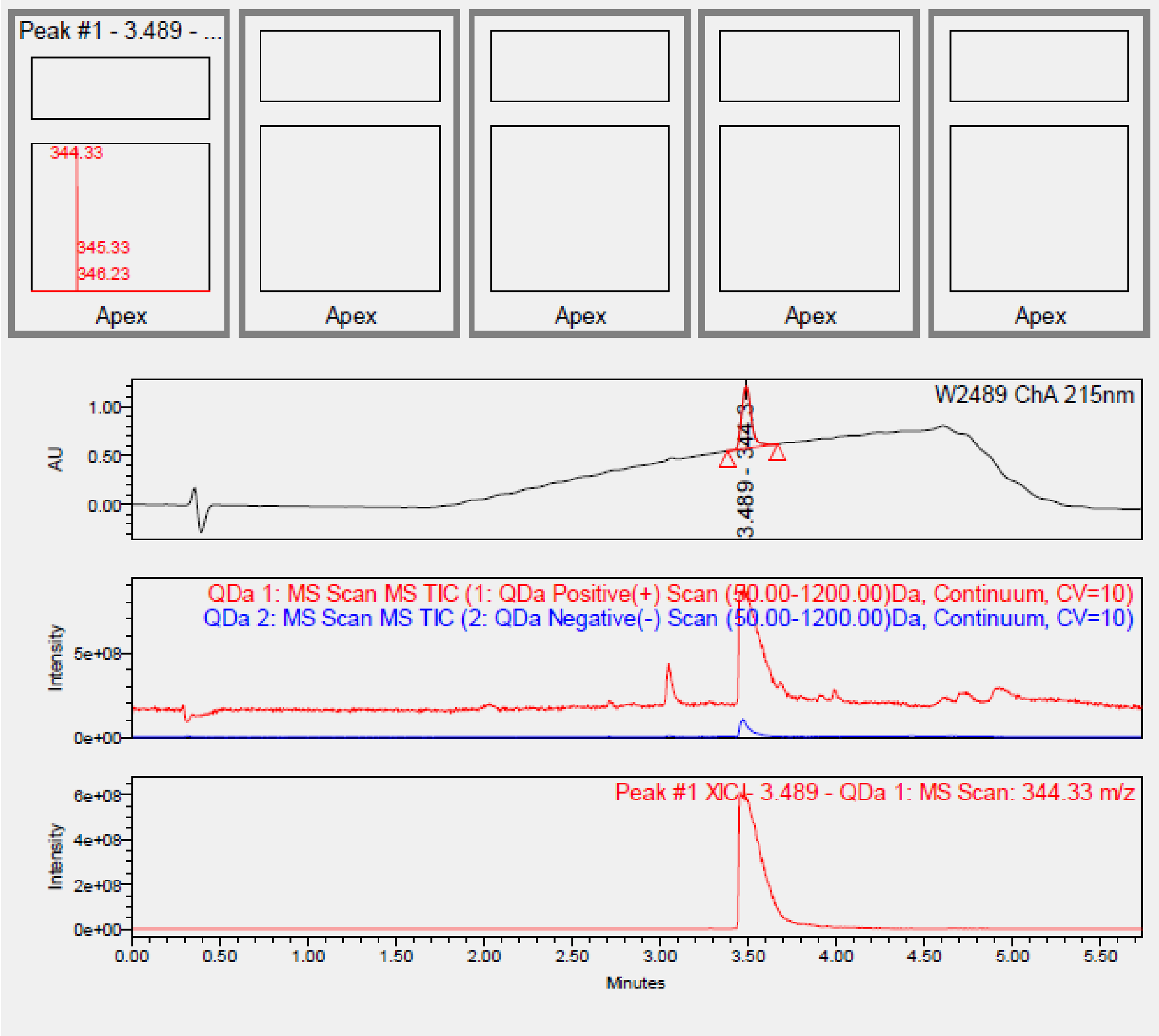

^{1}H-NMR spectrum (401 MHz, DMSO-d_6) of **8**

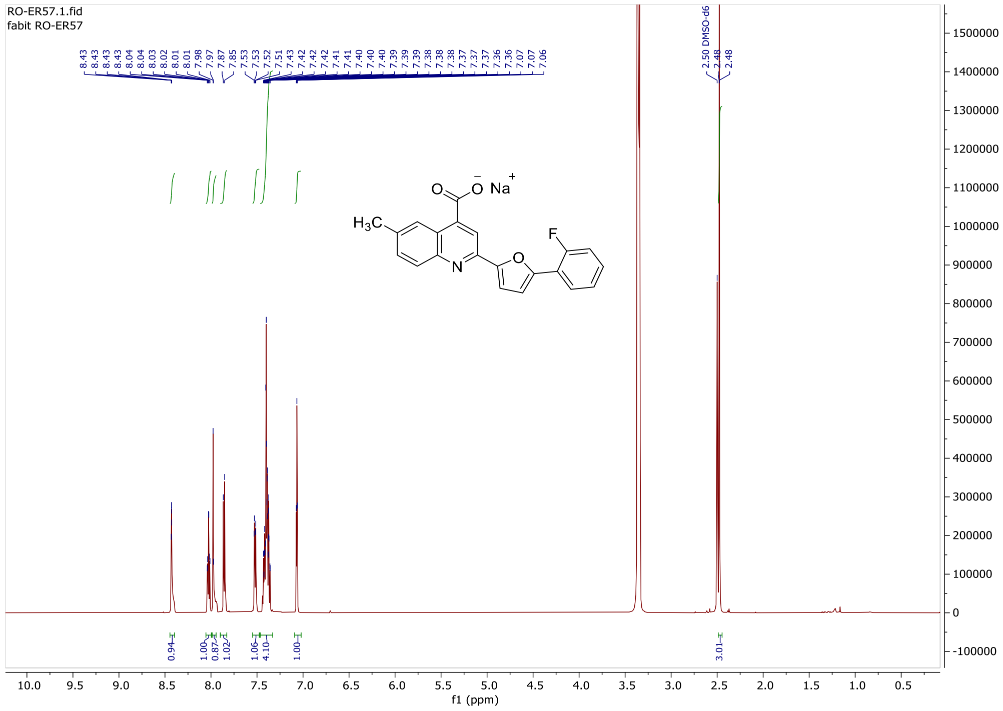

^{13}C-NMR spectrum (101 MHz, DMSO-d_6) of **8**

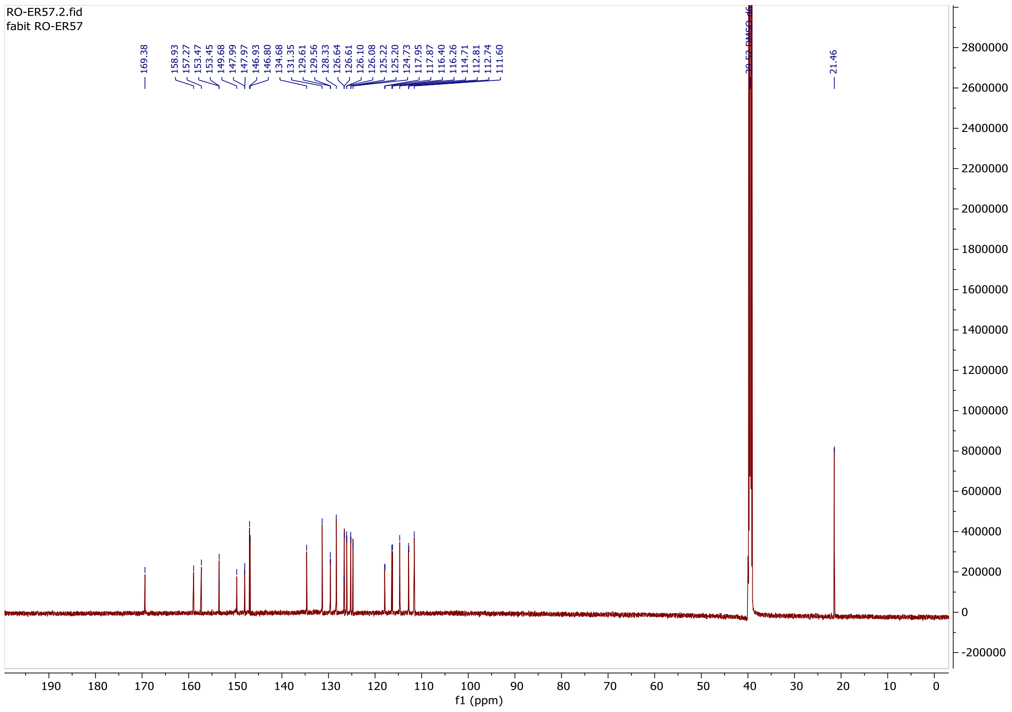

UHPLC-MS analysis of **8**

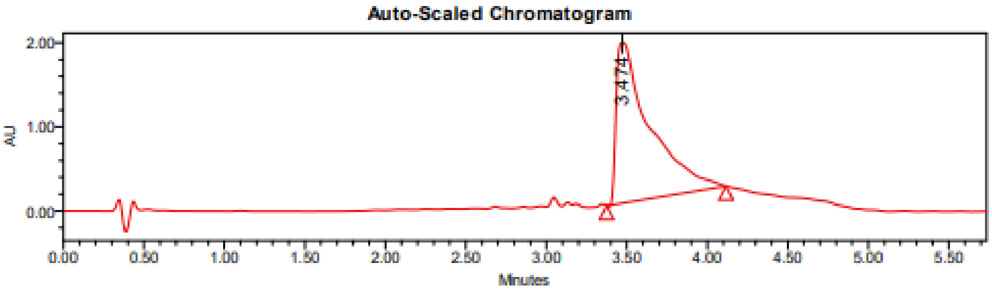

Processed Channel: W2489 ChA 215nm

	Processed Channel	Retention Time (min)	Area	% Area	Height
1	W2489 ChA 215nm	3.474	27910419	100.00	1920230

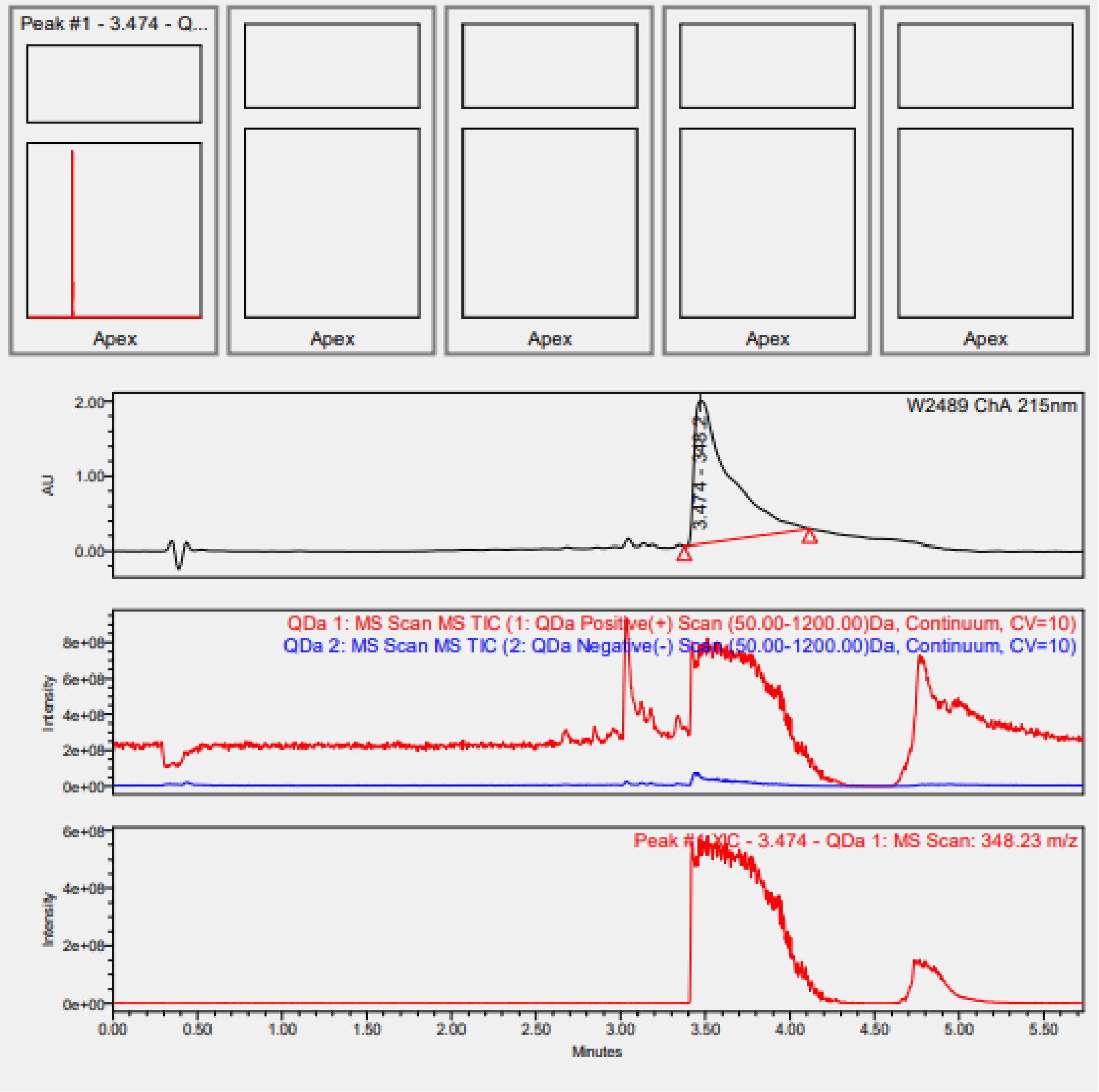

^{1}H-NMR spectrum (401 MHz, DMSO-d_6) of **9**

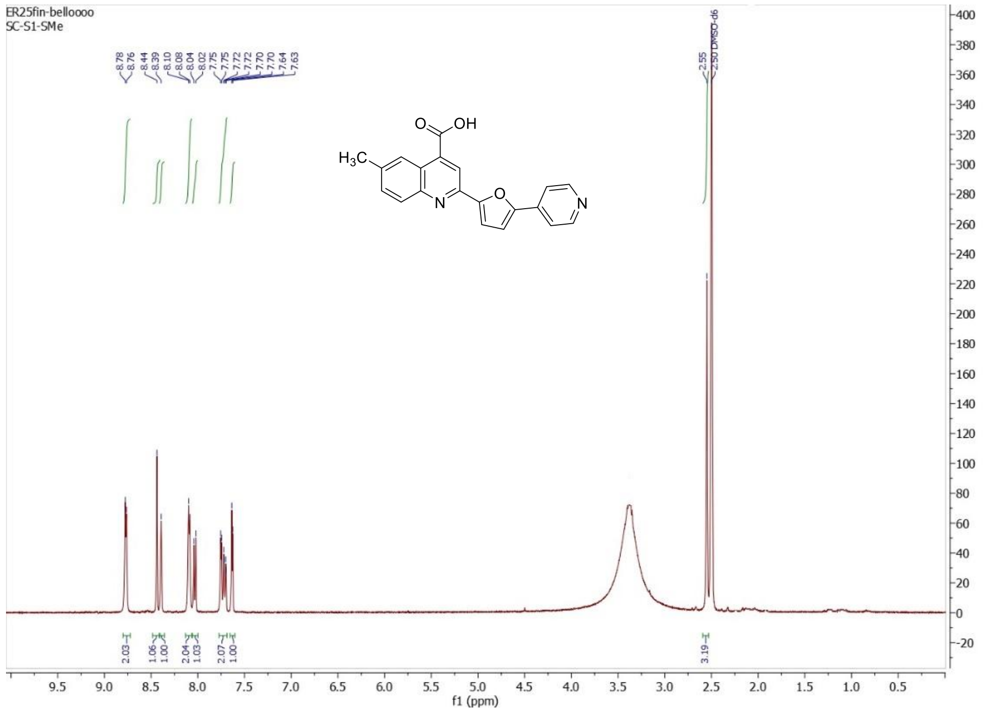

^{13}C-NMR spectrum (401 MHz, DMSO-d_6) of **9**

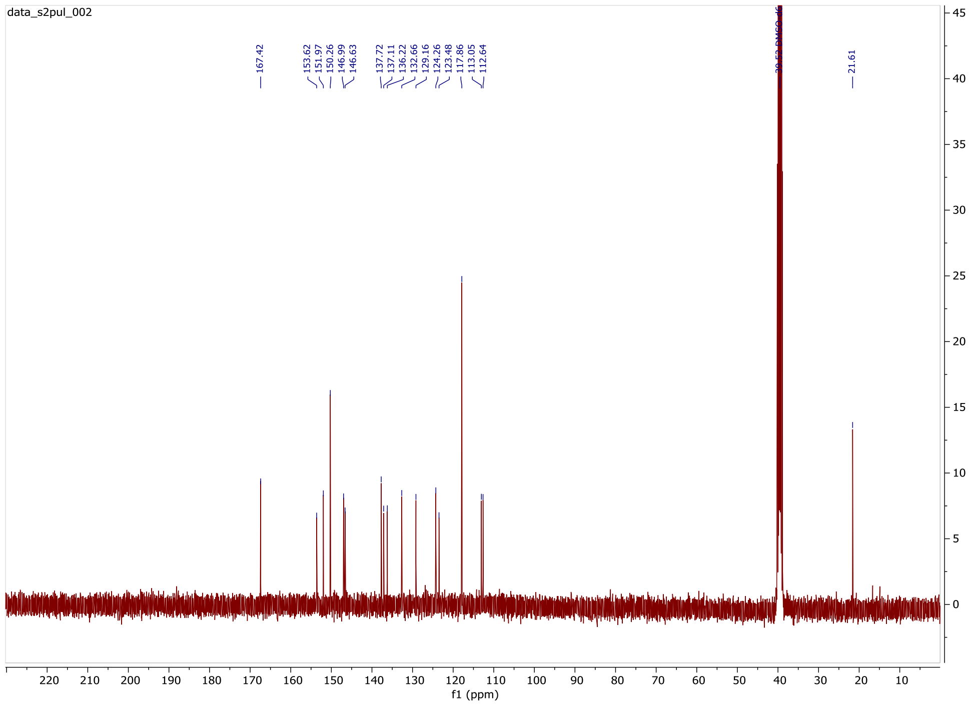

UHPCL-MS analysis of **9**

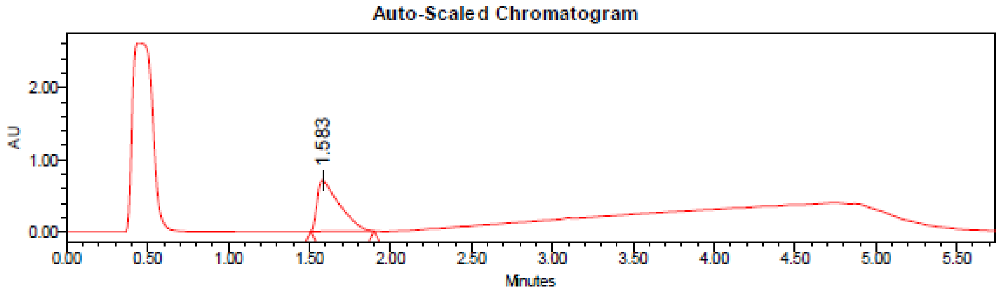

Processed Channel: W2489 ChA 215nm

	Processed Channel	Retention Time (min)	Area	% Area	Height
1	W2489 ChA 215nm	1.583	6706741	100.00	709097

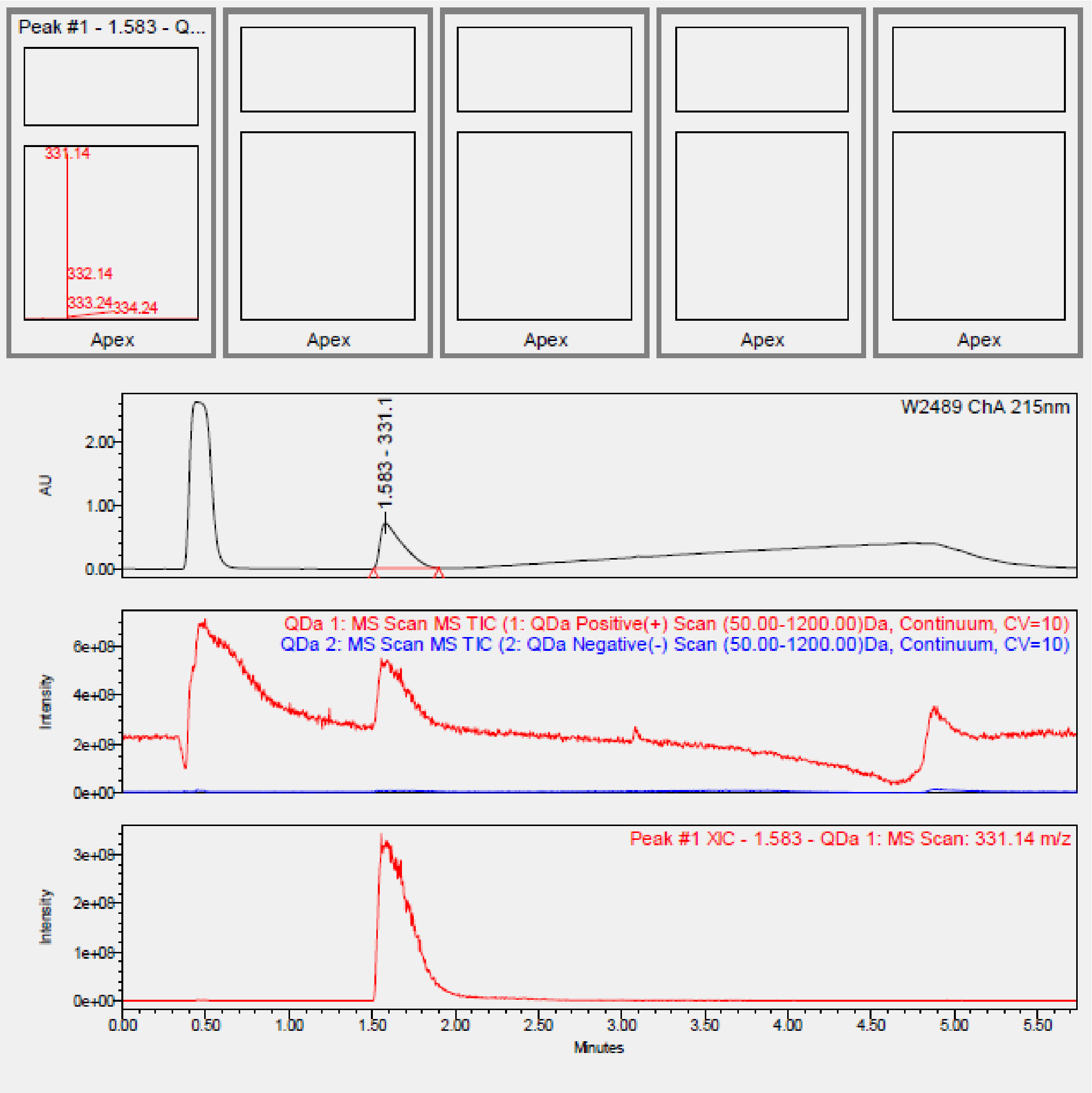

^{1}H-NMR spectrum (401 MHz, DMSO-d_6) of **10**

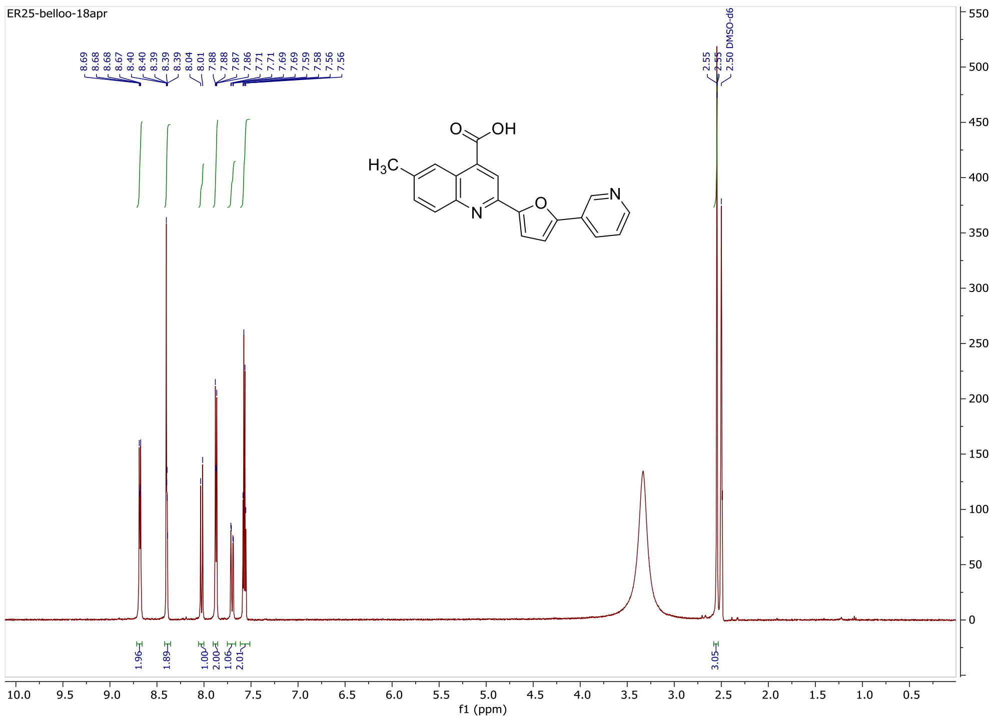

^{13}C-NMR spectrum (101 MHz, DMSO-d_6) of **10**

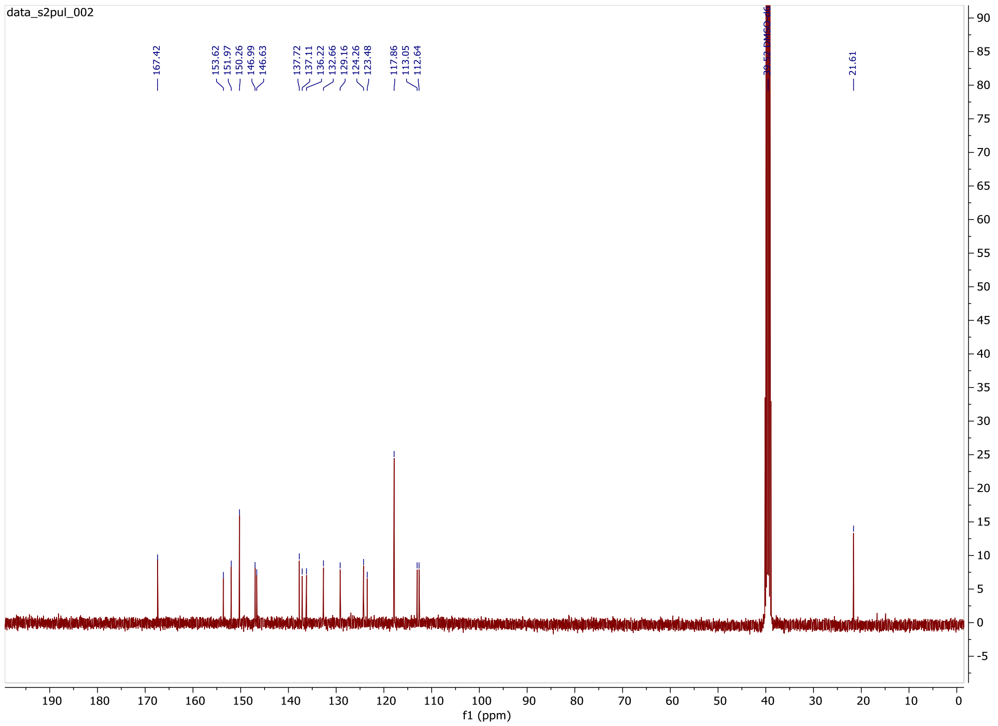

UHPLC-MS analysis of **10**

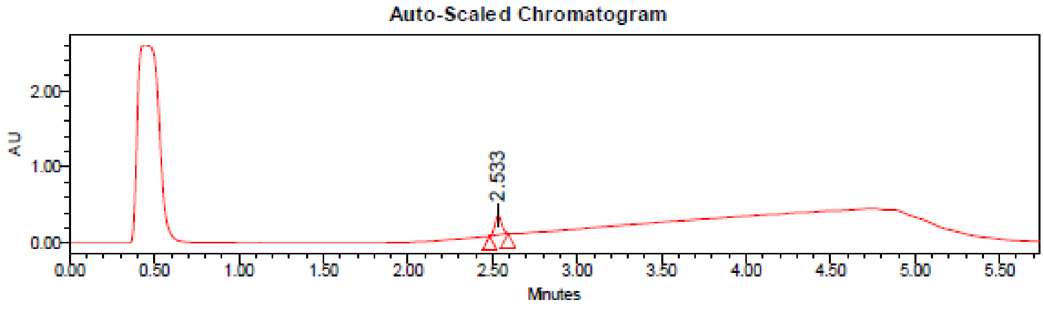

Processed Channel: W2489 ChA 215nm

	Processed Channel	Retention Time (min)	Area	% Area	Height
1	W2489 ChA 215nm	2.533	695789	100.00	255682

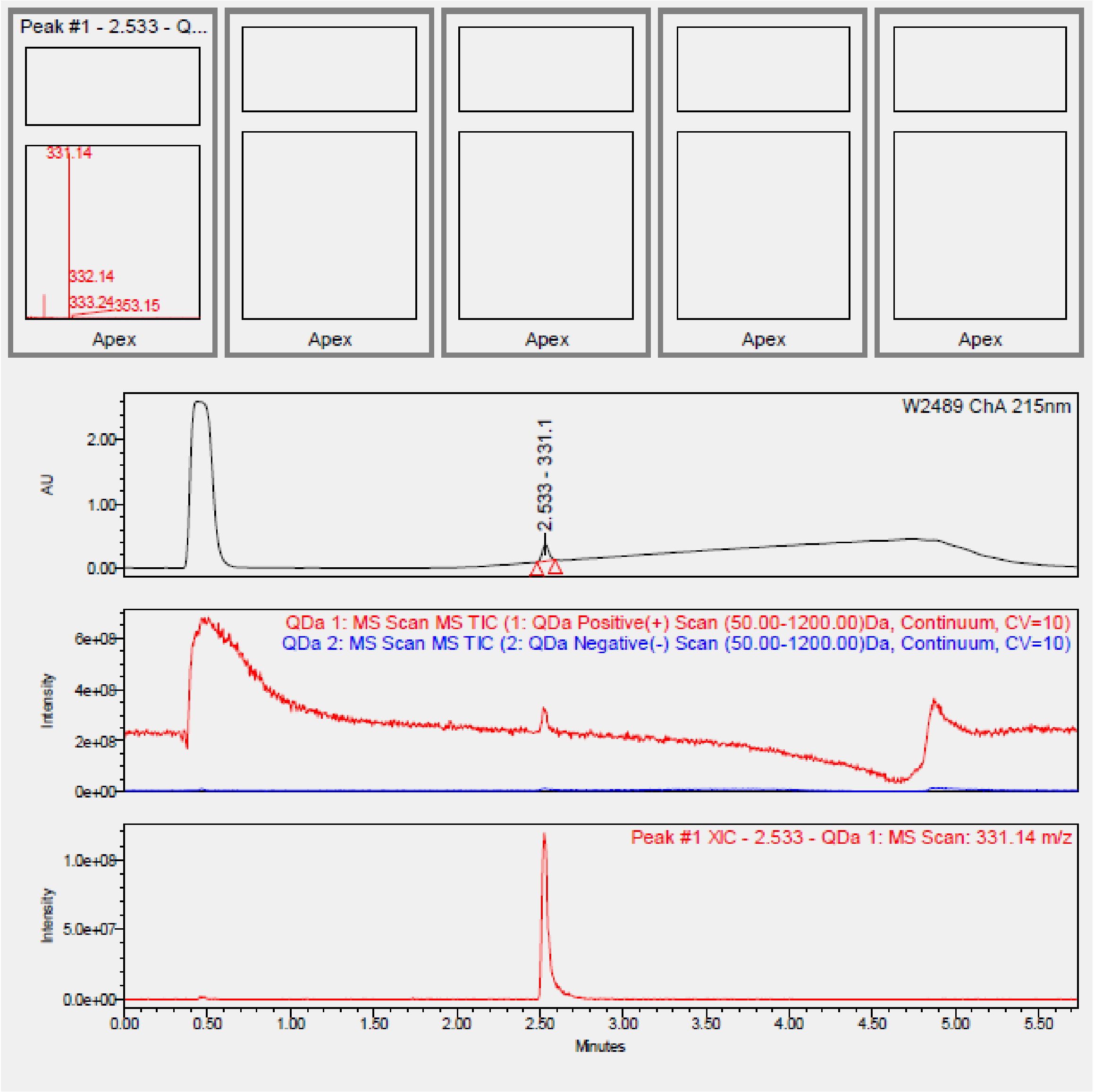

^{1}H-NMR spectrum (401 MHz, DMSO-d_6) of **11**

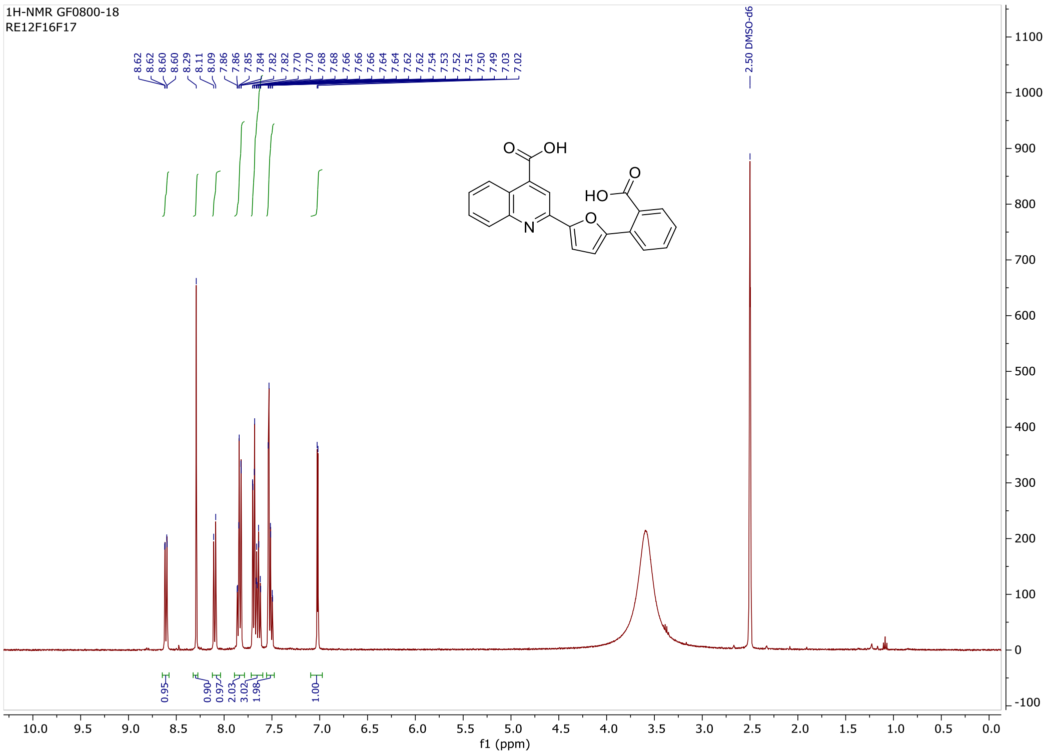

^{13}C-NMR spectrum (101 MHz, DMSO-d_6) of **11**

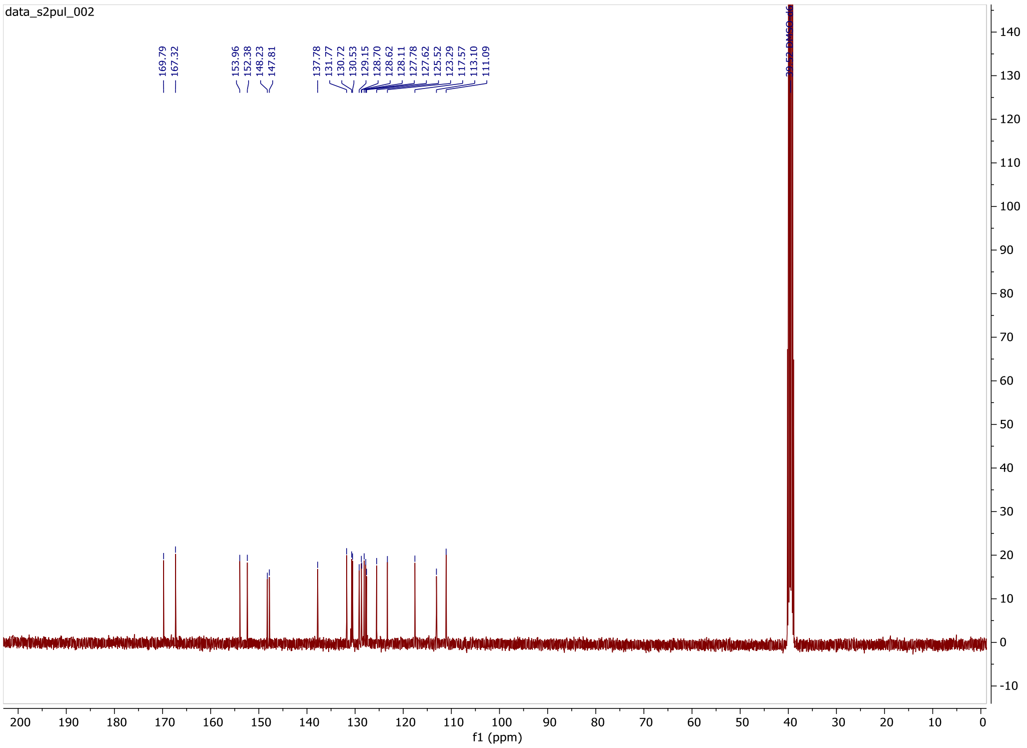

UHPLC-MS analysis of **11**

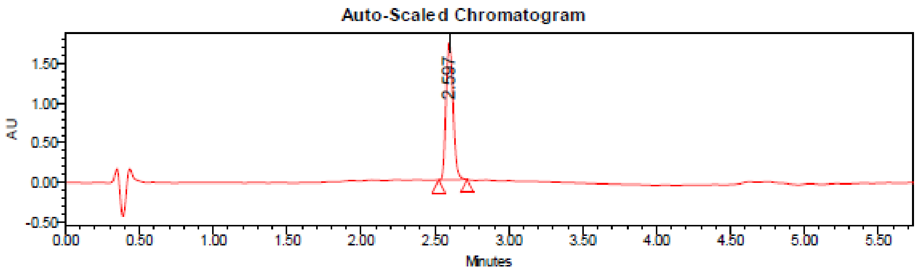

Processed Channel: W2489 ChA 215nm

	Processed Channel	Retention Time (min)	Area	% Area	Height
1	W2489 ChA 215nm	2.597	5733908	100.00	1739226

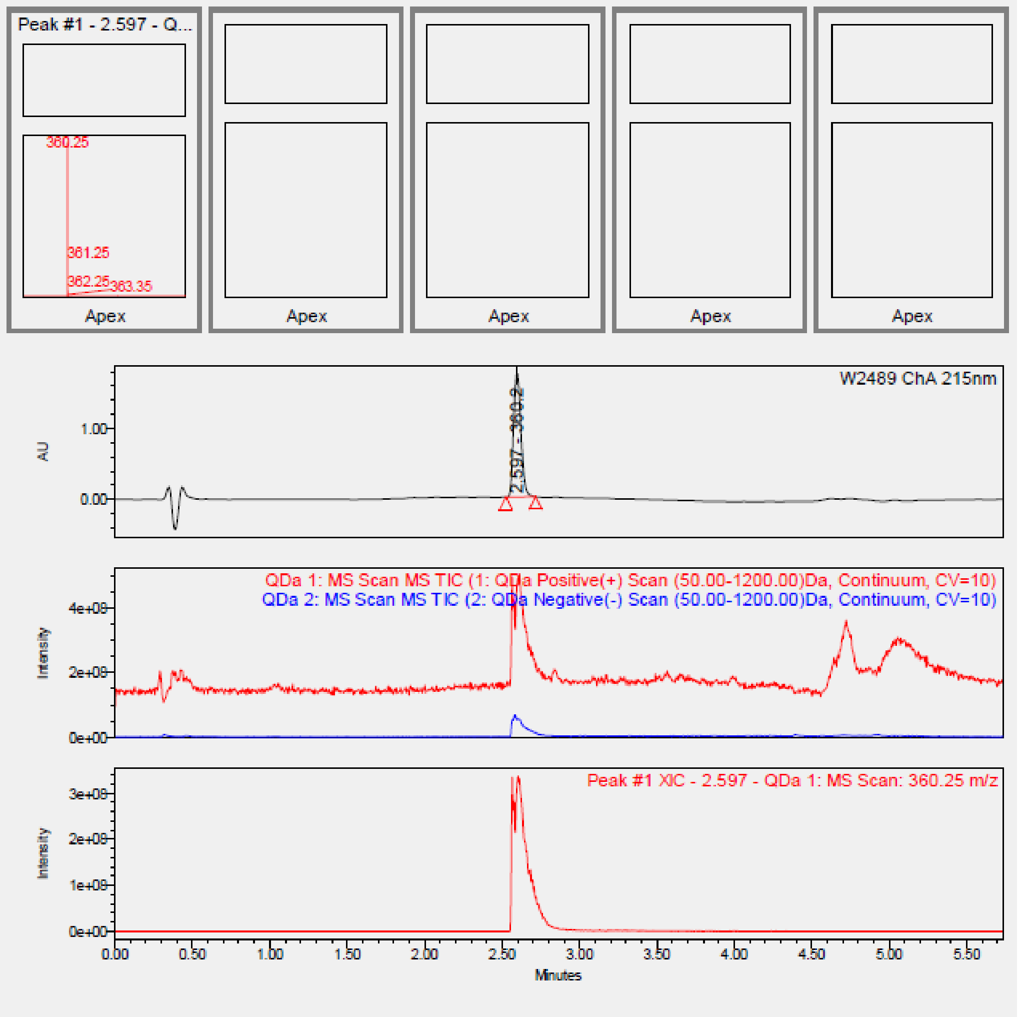

^{1}H-NMR spectrum (401 MHz, DMSO-d_6) of **12**

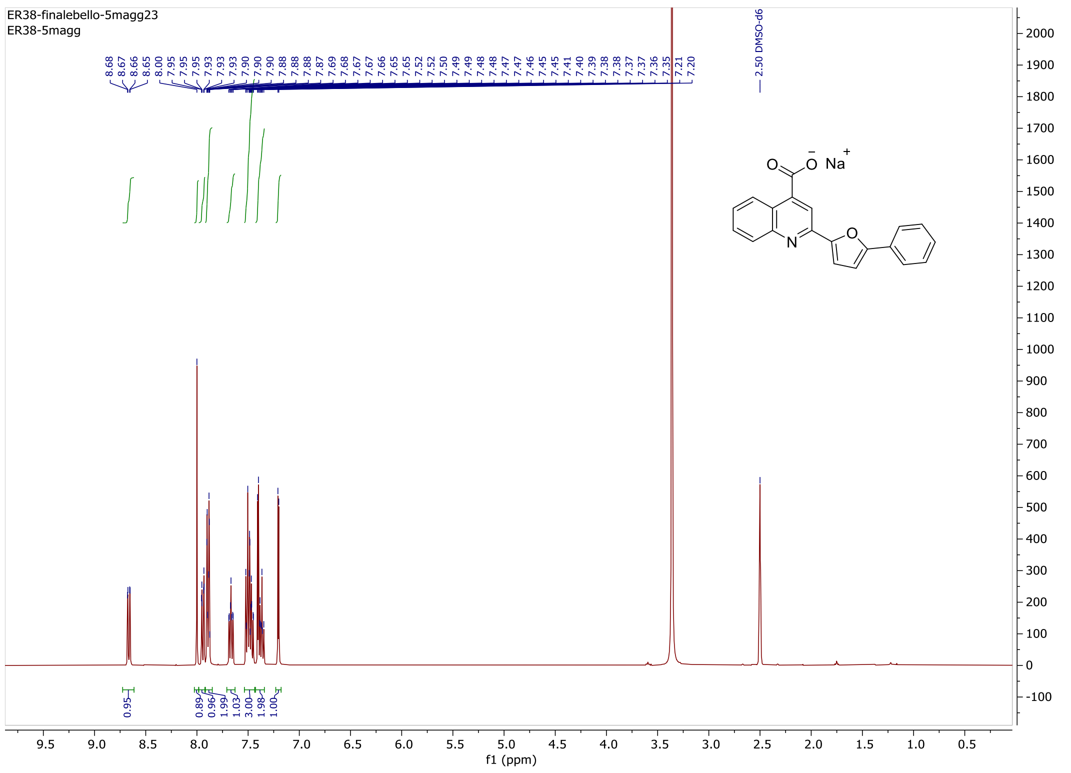

^{13}C-NMR spectrum (101 MHz, DMSO-d_6) of **12**

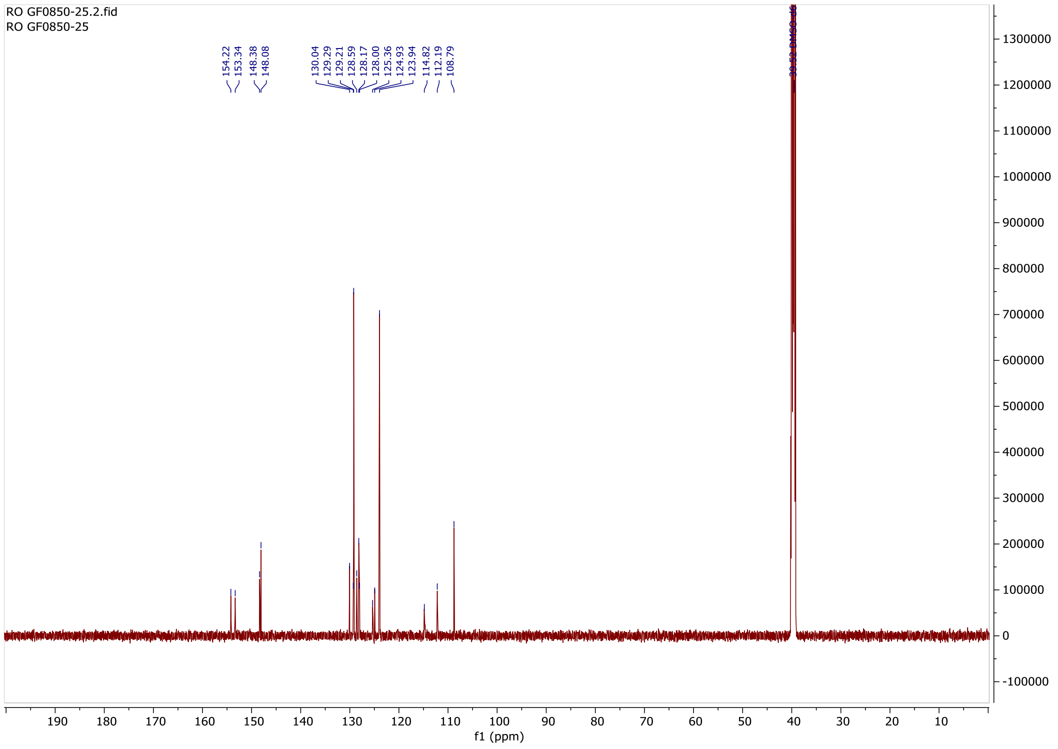

UHPLC-MS analysis of **12**

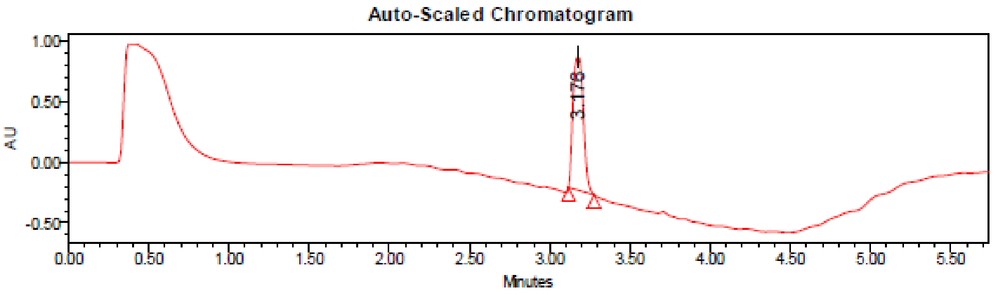

Processed Channel: W2489 ChA 215nm

	Processed Channel	Retention Time (min)	Area	% Area	Height
1	W2489 ChA 215nm	3.176	4913533	100.00	1107904

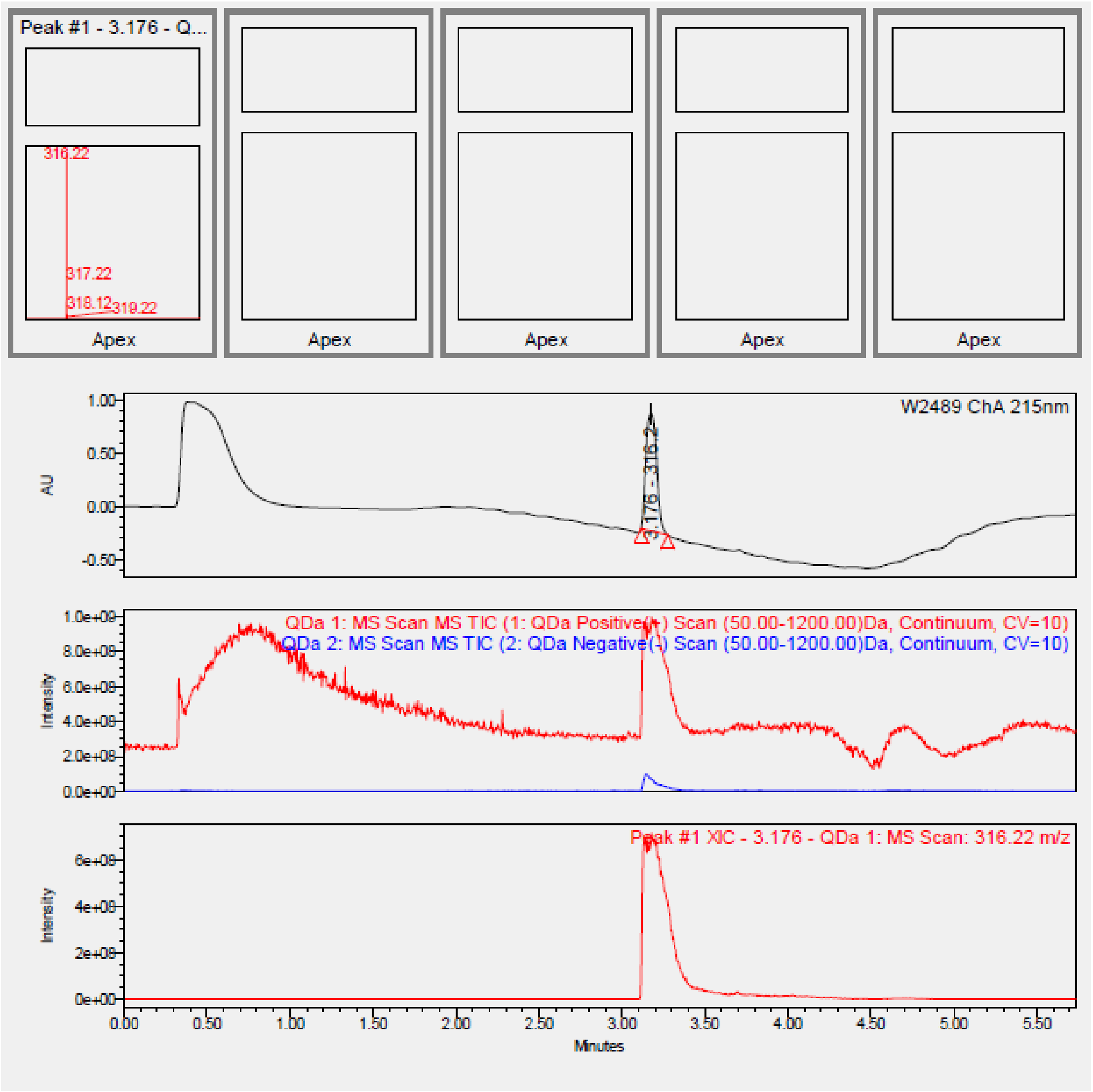

^{1}H-NMR spectrum (401 MHz, DMSO-d_6) of **13**

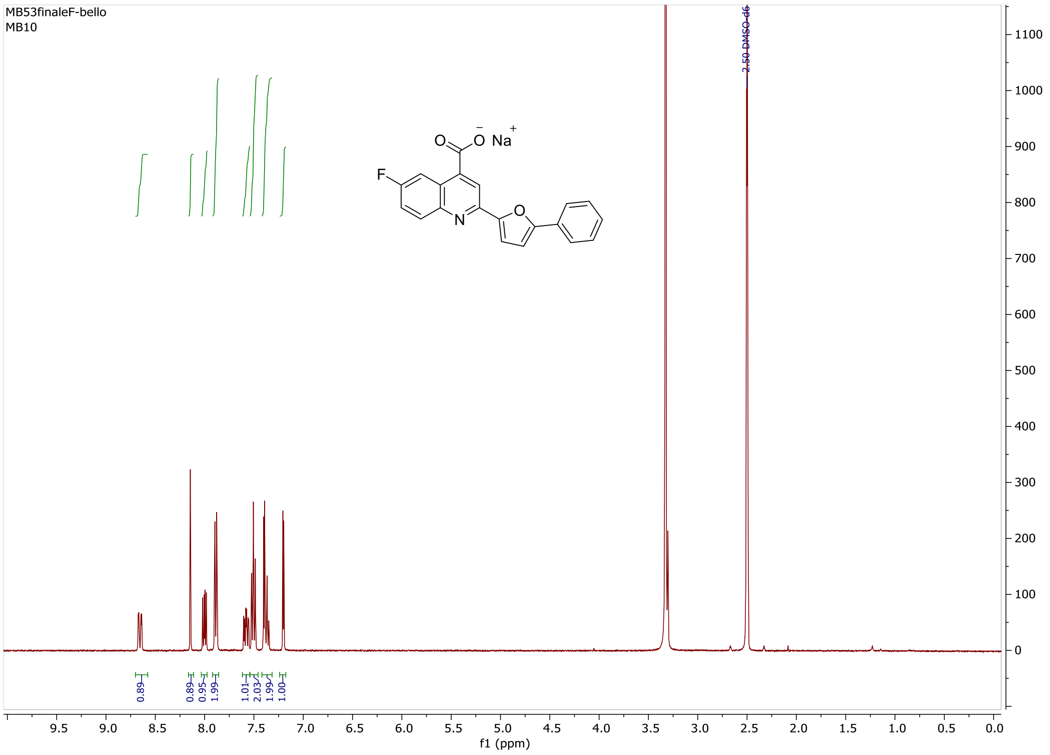

^{13}C-NMR spectrum (101 MHz, DMSO-d_6) of **13**

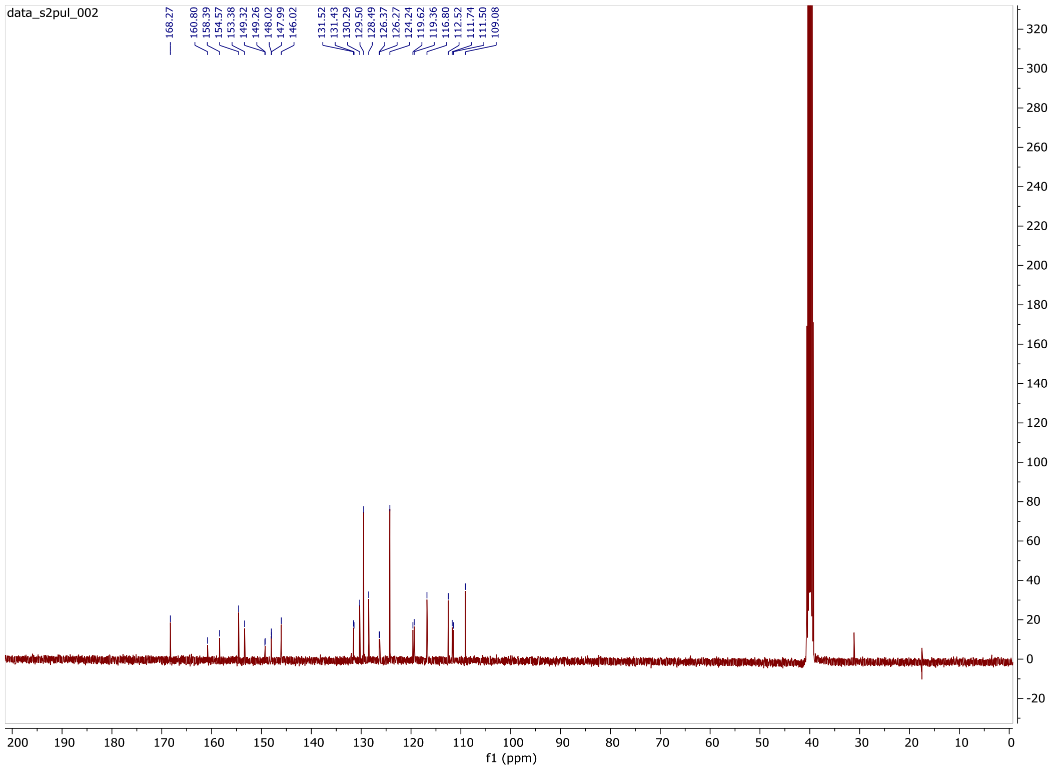

UHPLC-MS analysis of **13**

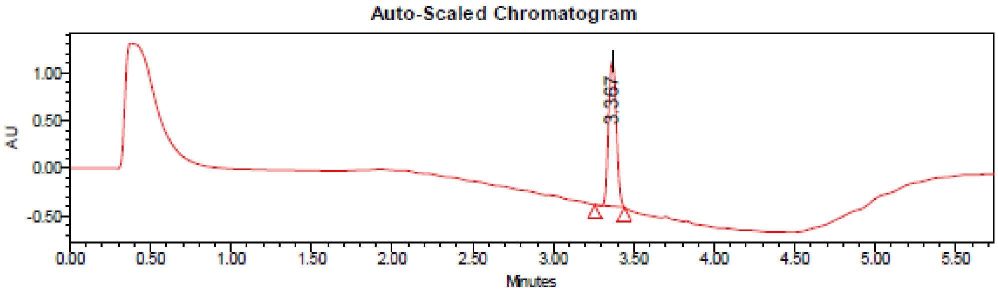

Processed Channel: W2489 ChA 215nm

	Processed Channel	Retention Time (min)	Area	% Area	Height
1	W2489 ChA 215nm	3.367	5062327	100.00	1516489

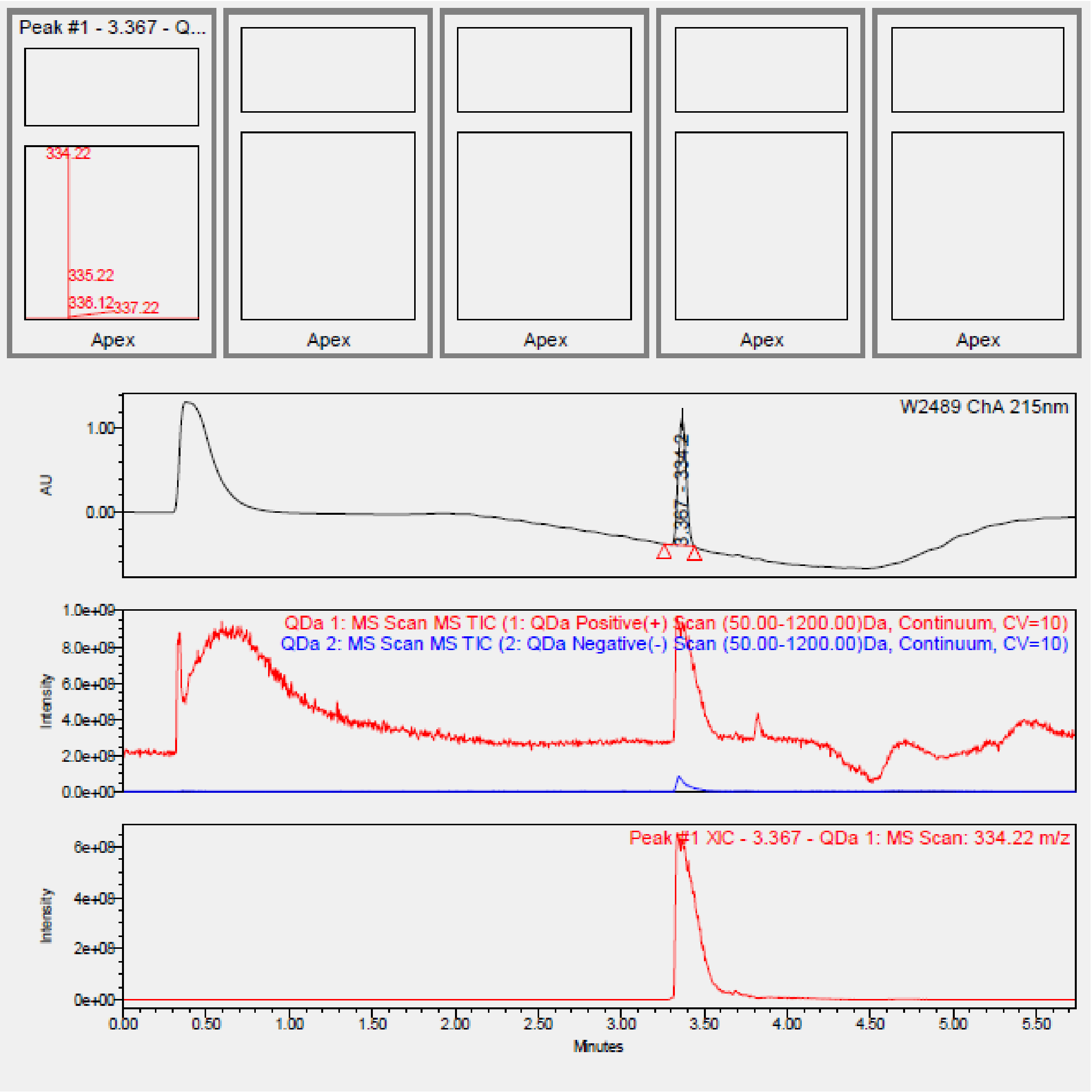

^{1}H-NMR spectrum (401 MHz, CD_3OD) of **14**

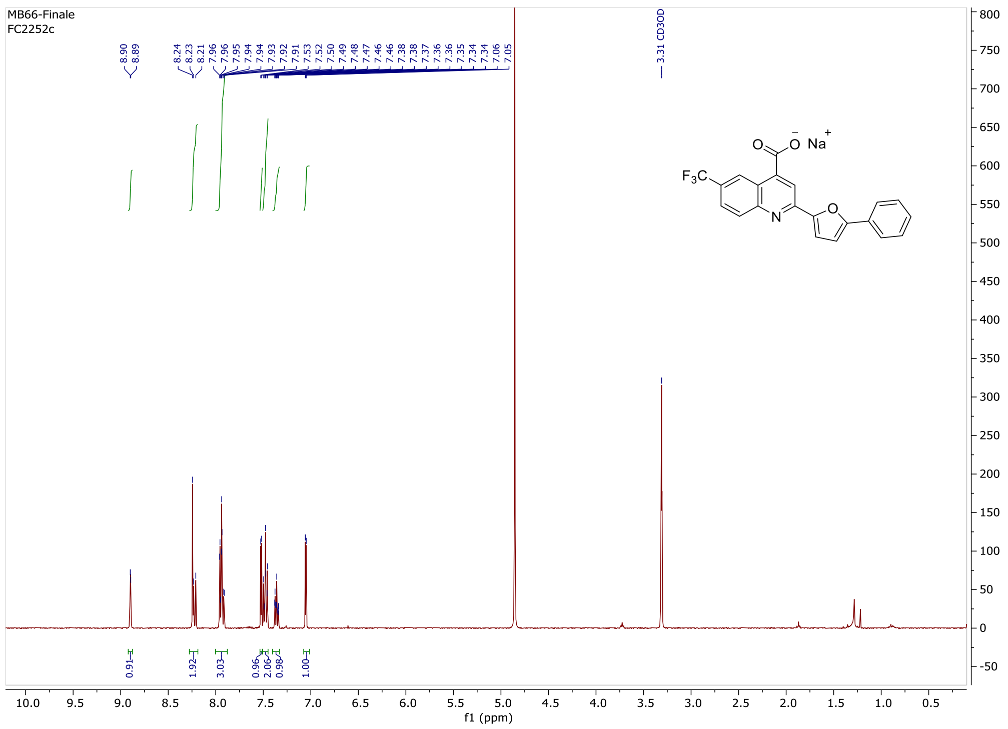

^{13}C-NMR spectrum (101 MHz, CD_3OD) of **14**

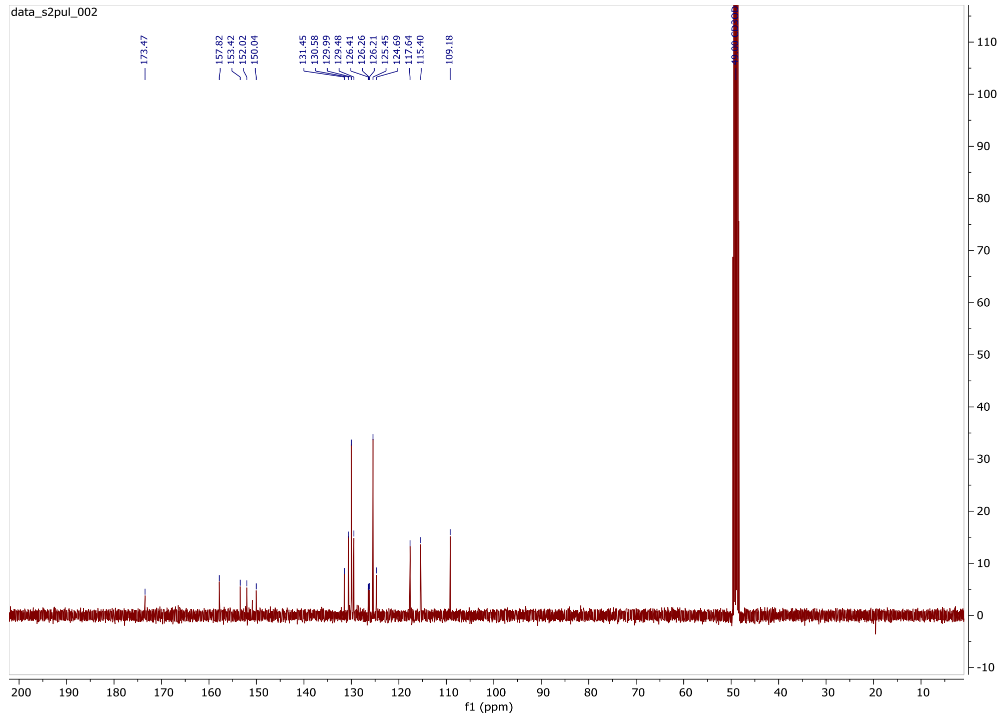

UHPLC-MS analysis of **14**

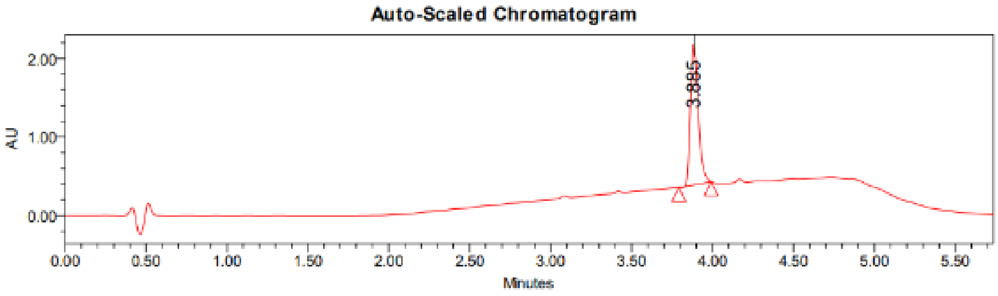

Processed Channel: W2489 ChA 215nm

	Processed Channel	Retention Time (min)	Area	% Area	Height
1	W2489 ChA 215nm	3.885	6061479	100.00	1793250

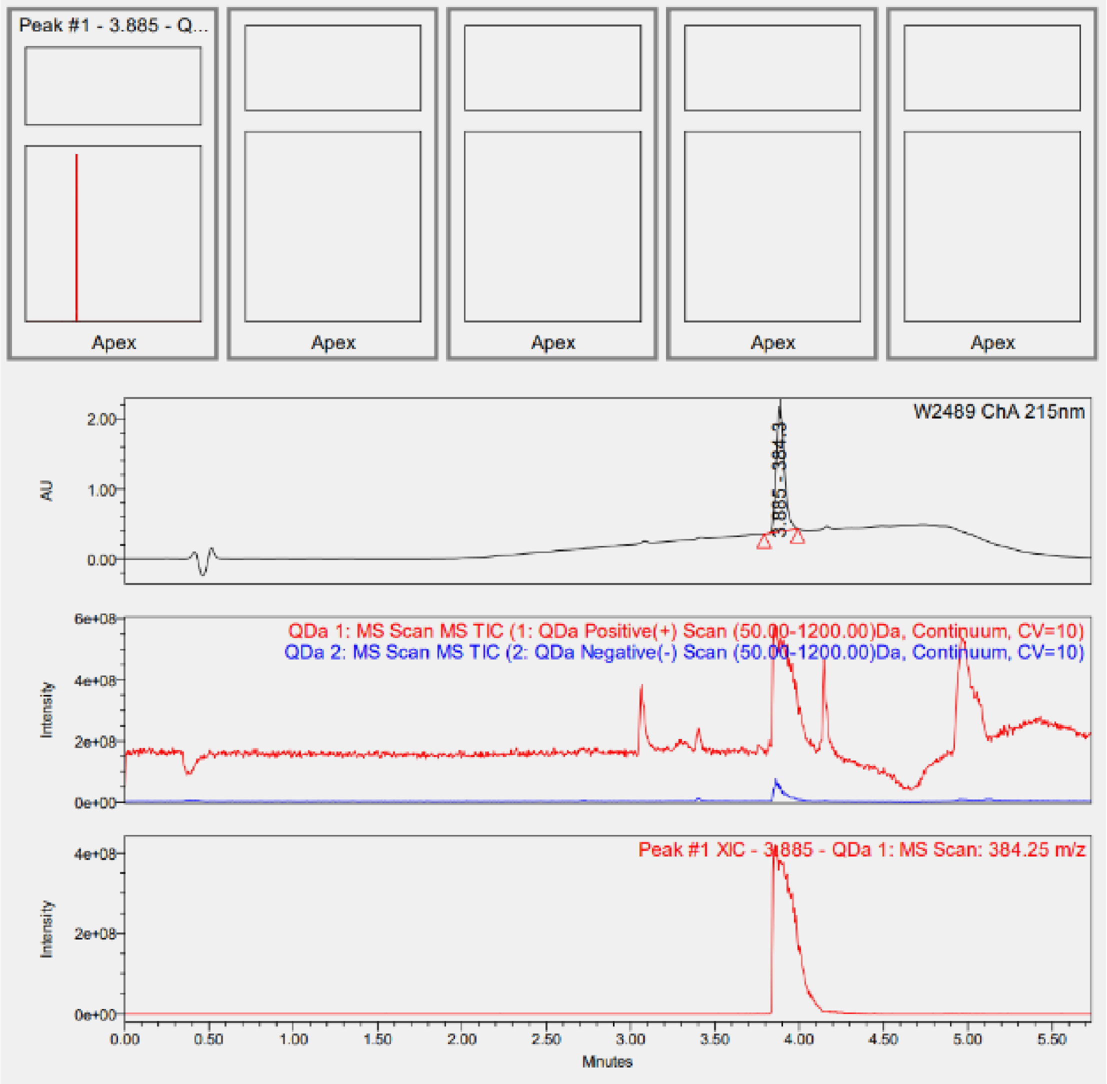

^{1}H-NMR spectrum (401 MHz, DMSO-d_6) of **15**

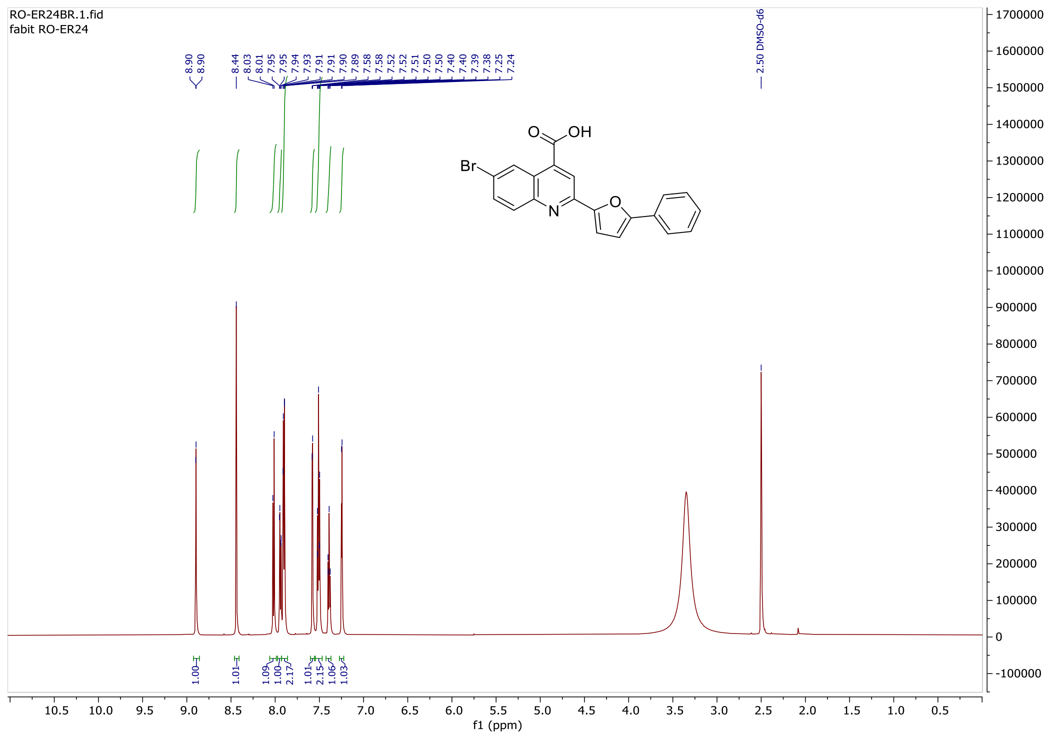

^{13}C-NMR spectrum (101 MHz, DMSO-d_6) of **15**

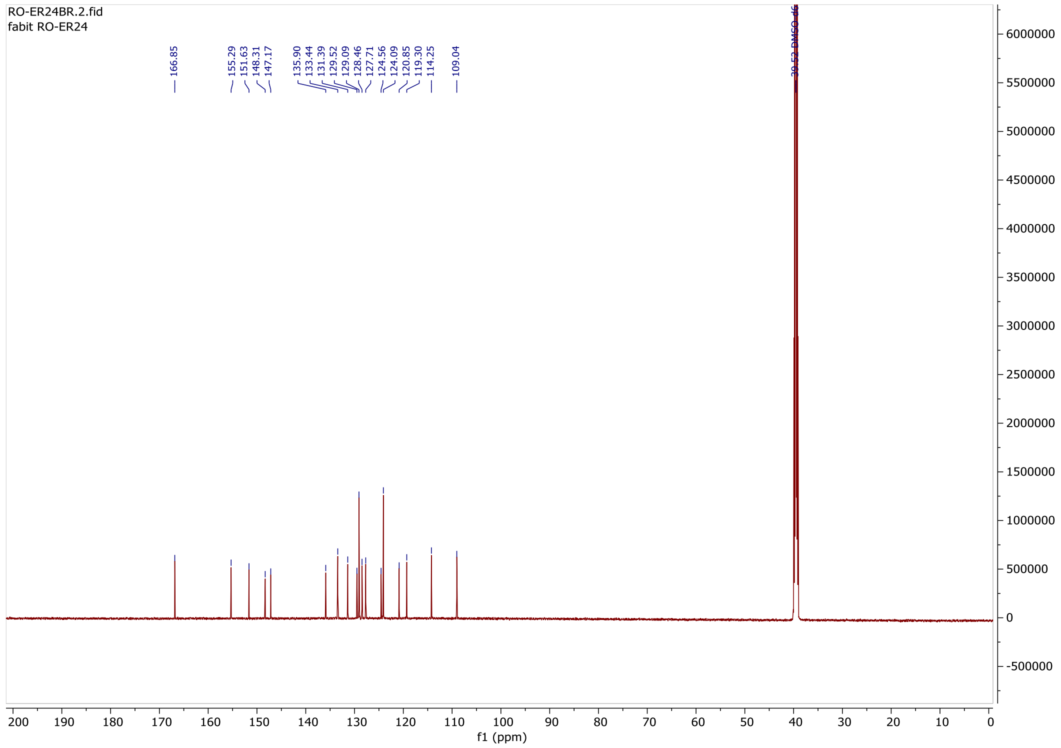

UHPLC-MS analysis of **15**

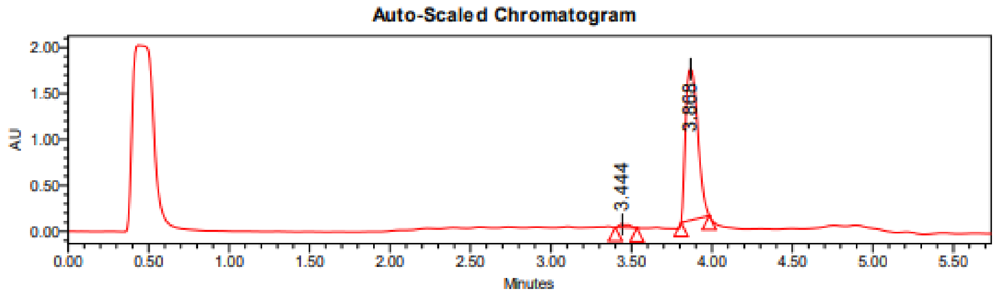

Processed Channel: W2489 ChA 215nm

	Processed Channel	Retention Time (min)	Area	% Area	Height
1	W2489 ChA 215nm	3.444	152167	1.74	34173
2	W2489 ChA 215nm	3.868	8618260	98.26	1649498

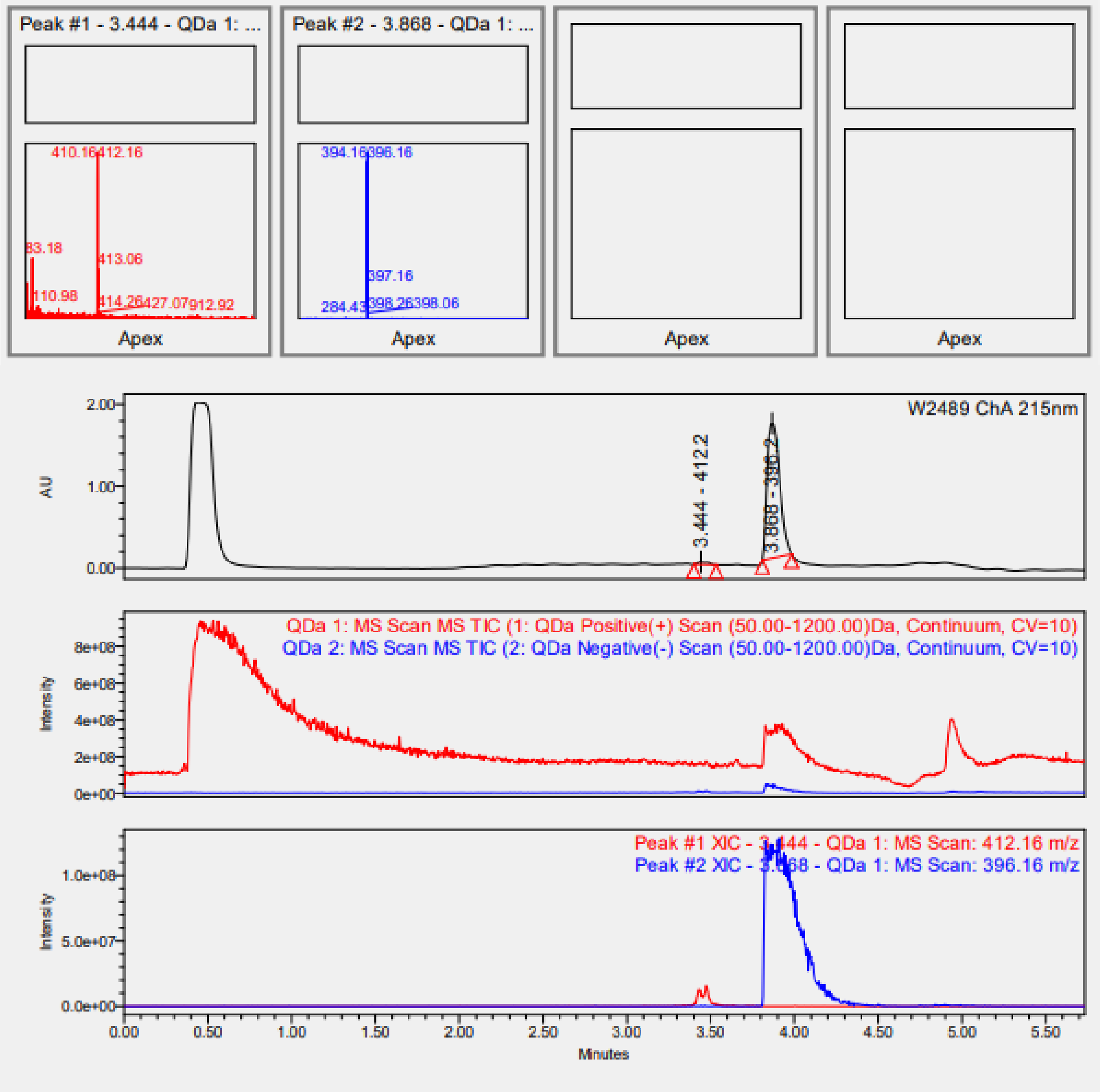

^1H-NMR spectrum (401 MHz, CD_3OD) of **16**

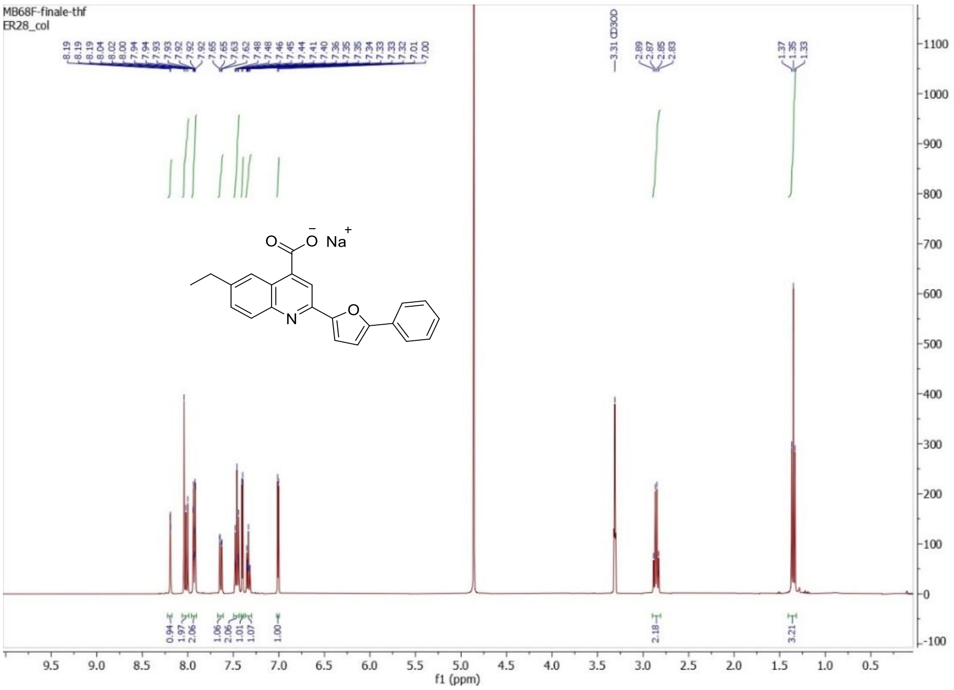

^{13}C-NMR spectrum (101 MHz, CD_3OD) of **16**

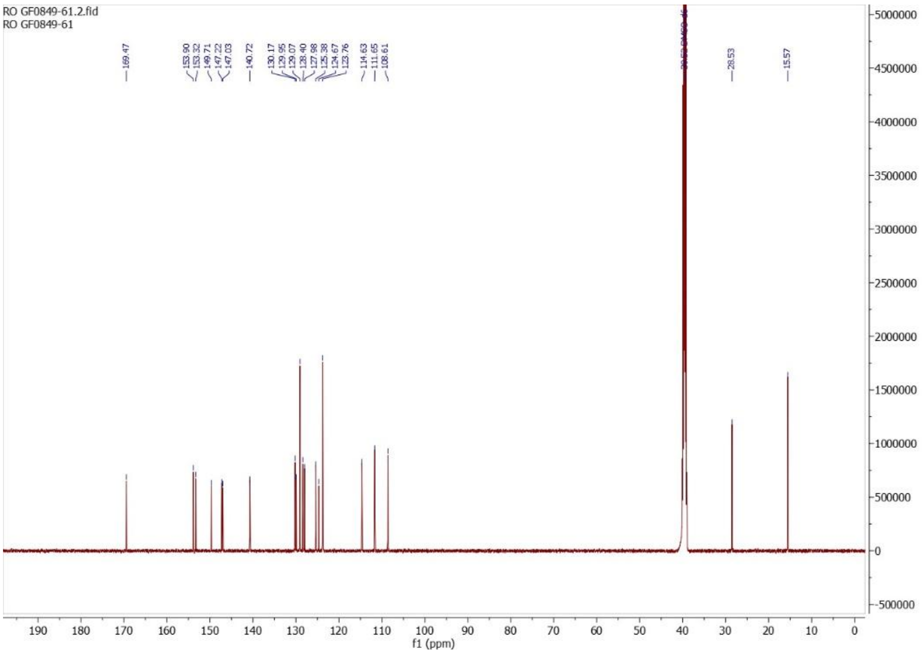

UHPLC-MS analysis of **16**

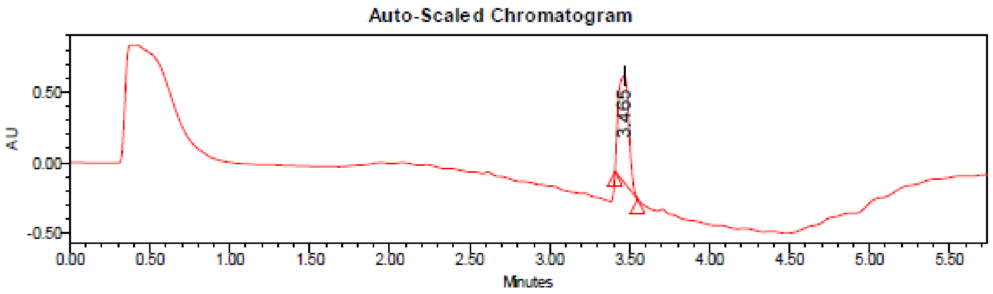

Processed Channel: W2489 ChA 215nm

	Processed Channel	Retention Time (min)	Area	% Area	Height
1	W2489 ChA 215nm	3.465	3462902	100.00	761998

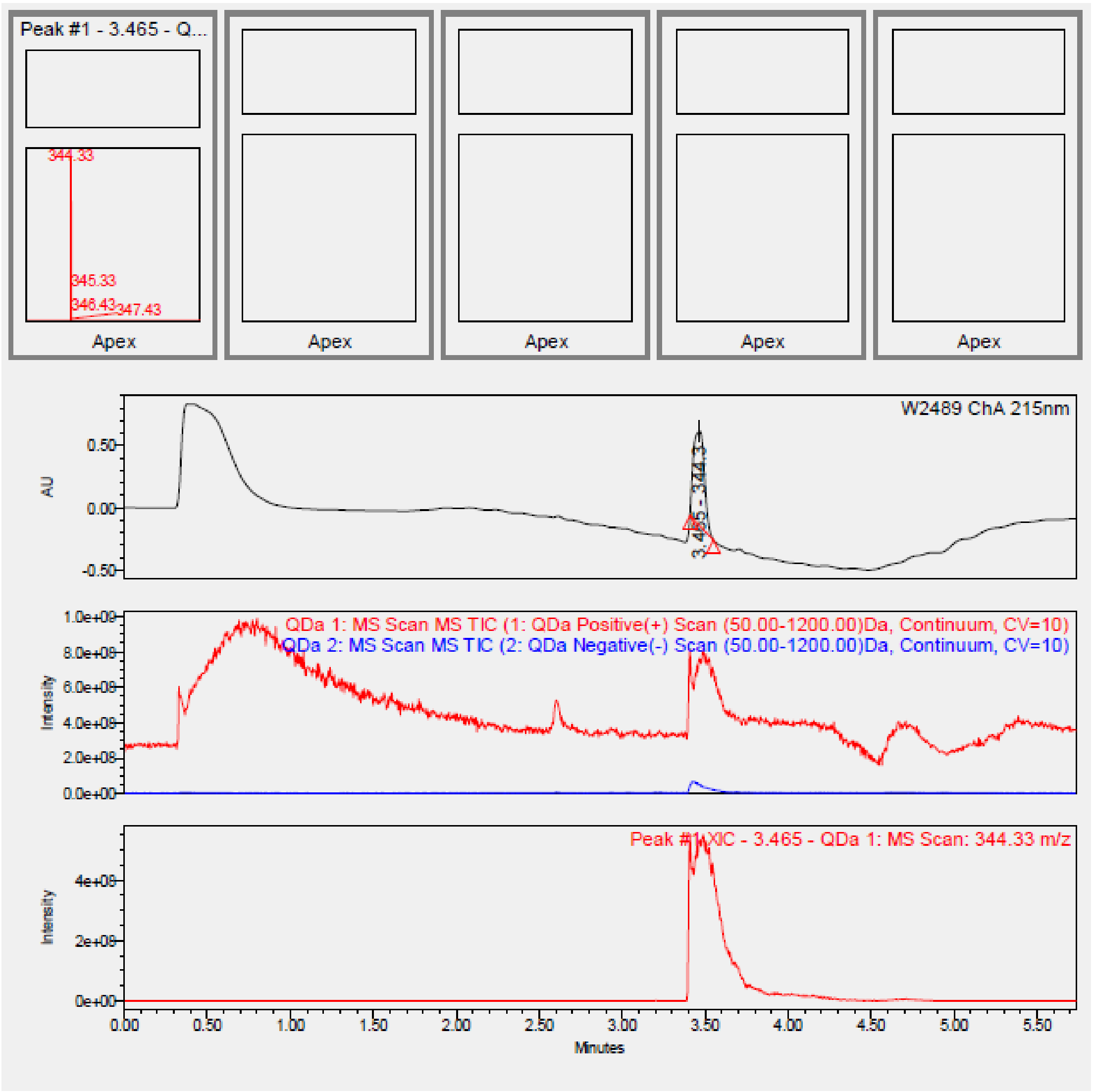

^{1}H-NMR spectrum (401 MHz, DMSO-d_6) of **17**

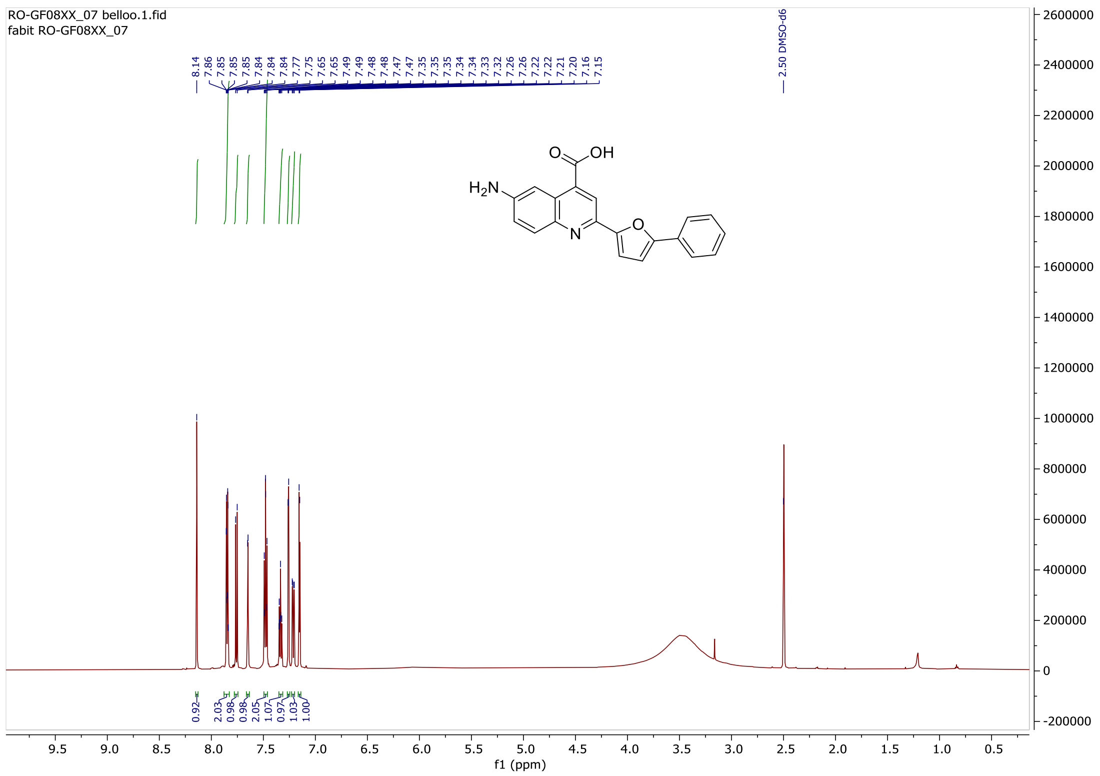

^{13}C-NMR spectrum (101 MHz, DMSO-d_6) of **17**

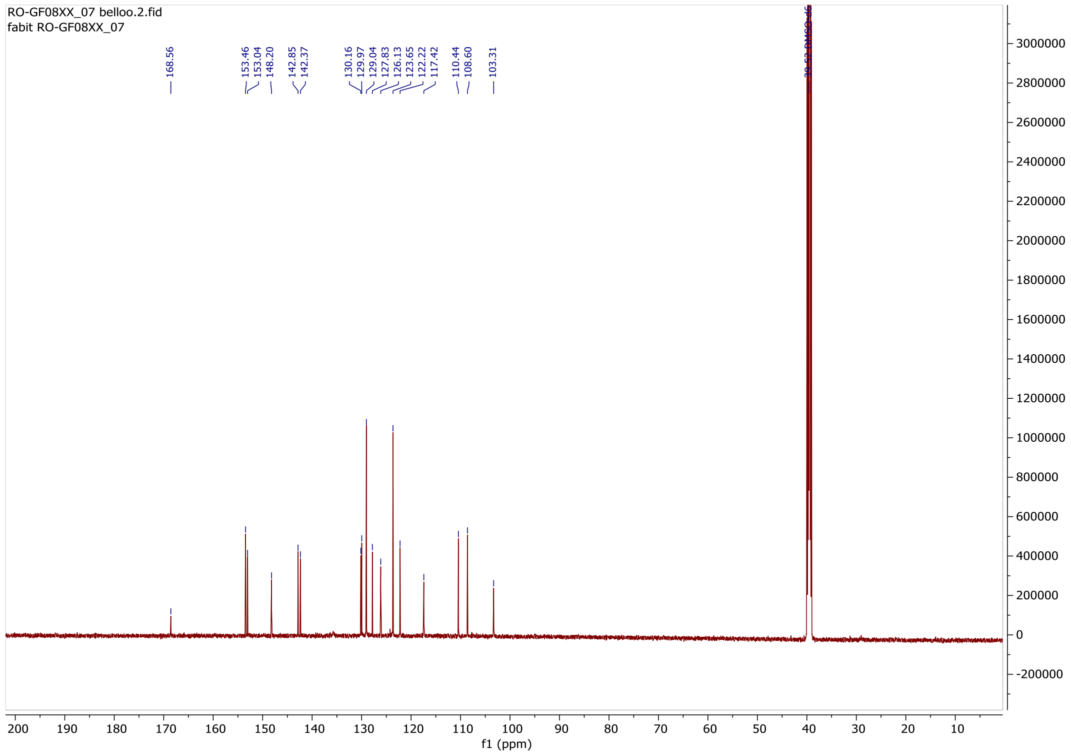

UHPLC-MS analysis of **17**

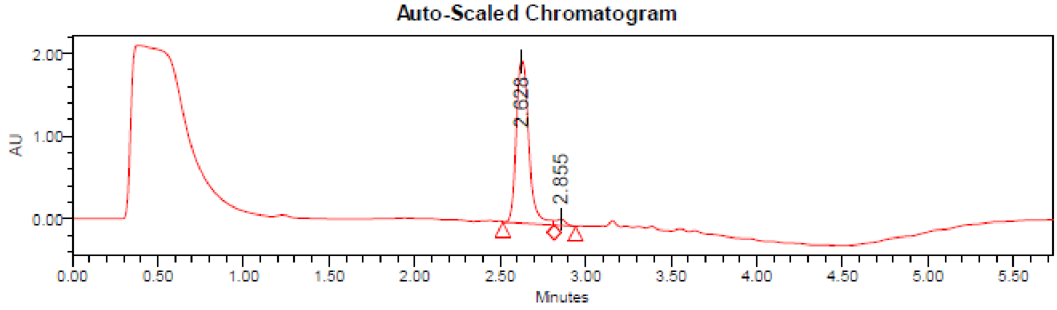

Peak Results

	Name	RT	Area	Height	% Area
1		2.628	10080030	1977545	97.23
2		2.855	287434	75756	2.77

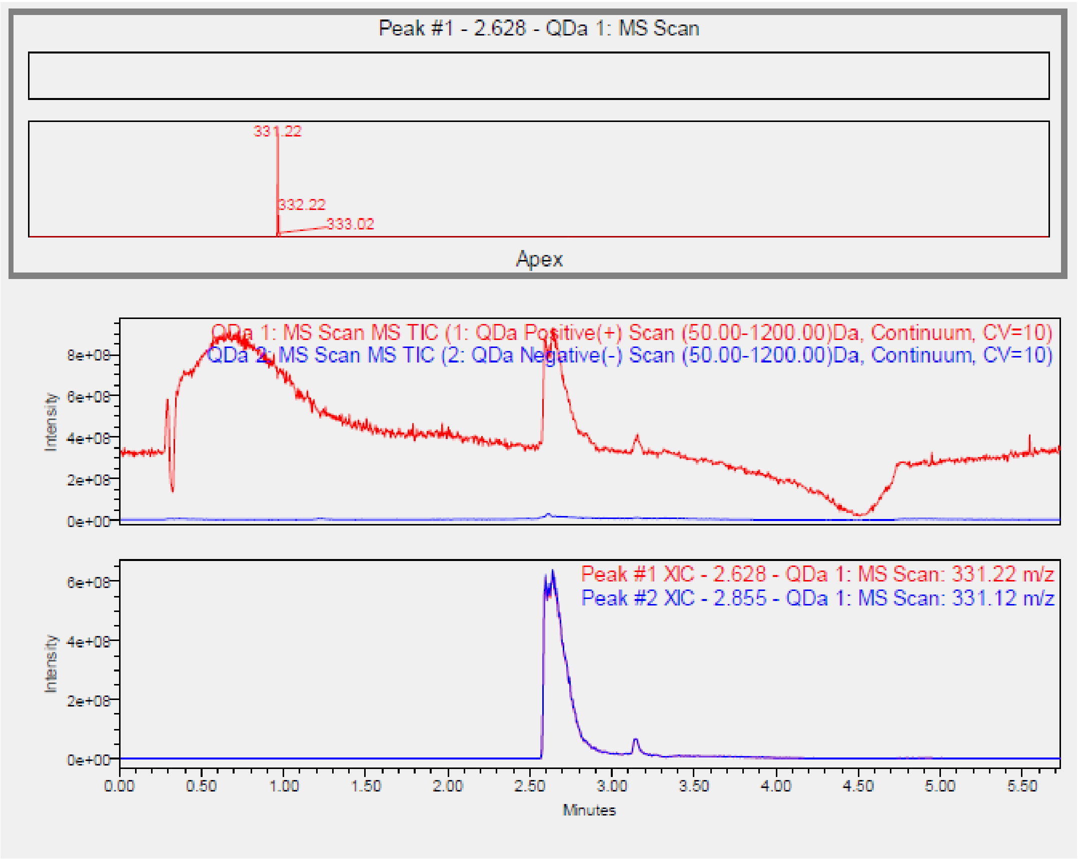

^{1}H-NMR spectrum (401 MHz, DMSO-d_6) of **18**

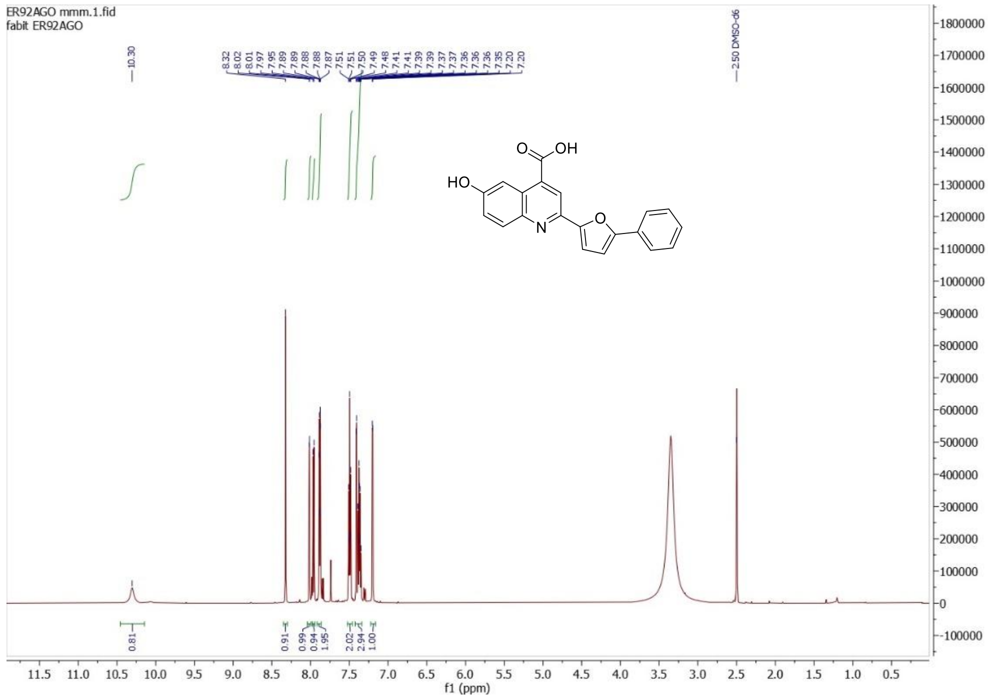

^{13}C-NMR spectrum (101 MHz, DMSO-d_6) of **18**

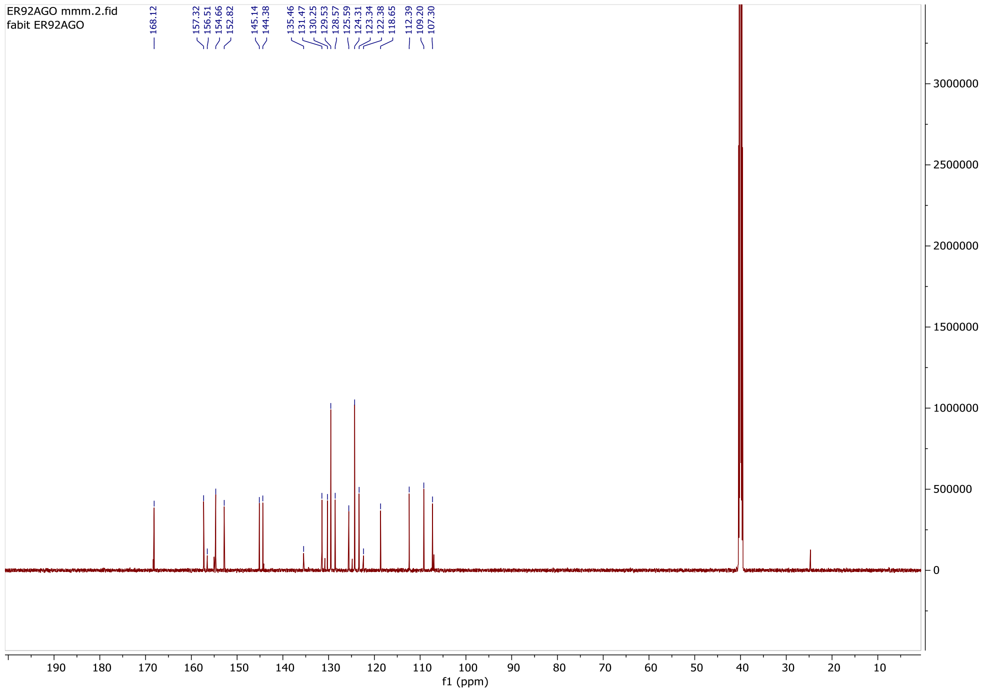

UHPLC-MS analysis of **18**

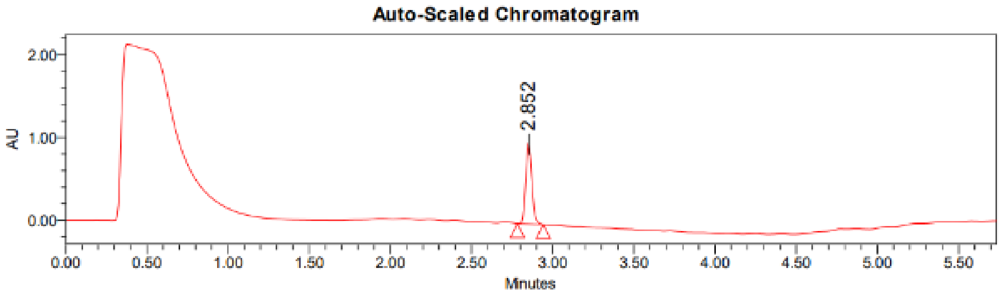

Peak Results

	Name	RT	Area	Height	% Area
1		2.852	2538808	964837	100.00

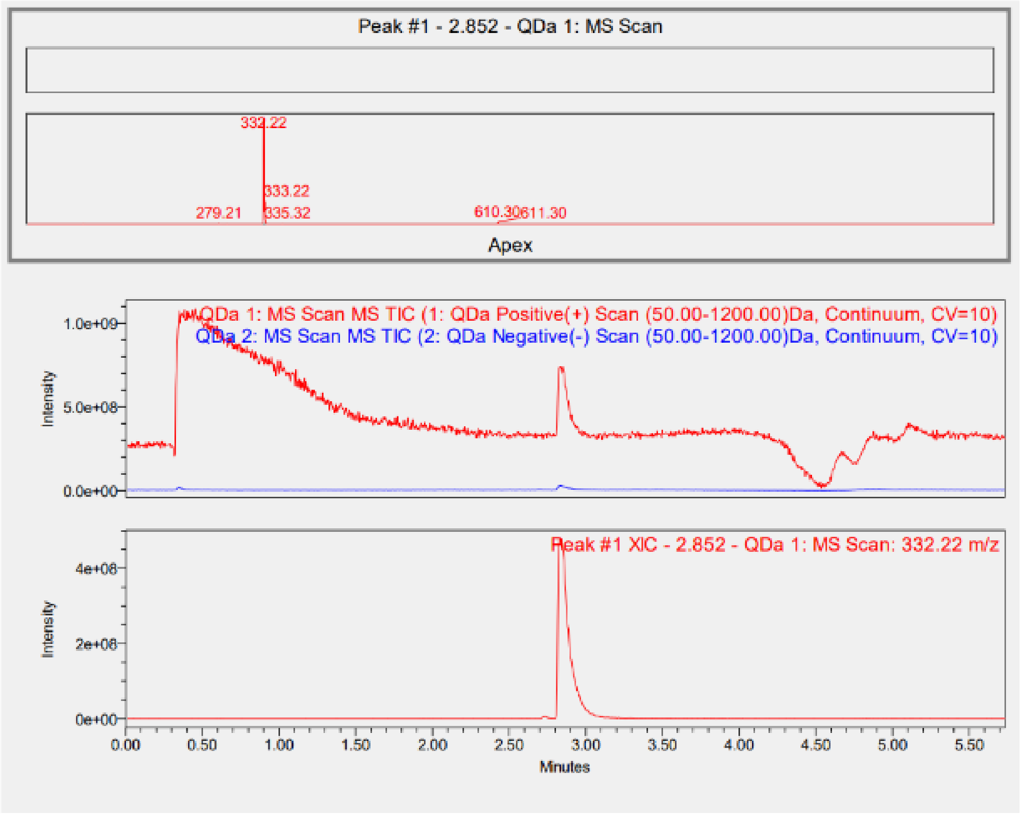

^{1}H-NMR spectrum (401 MHz, CD_3OD) of **19**

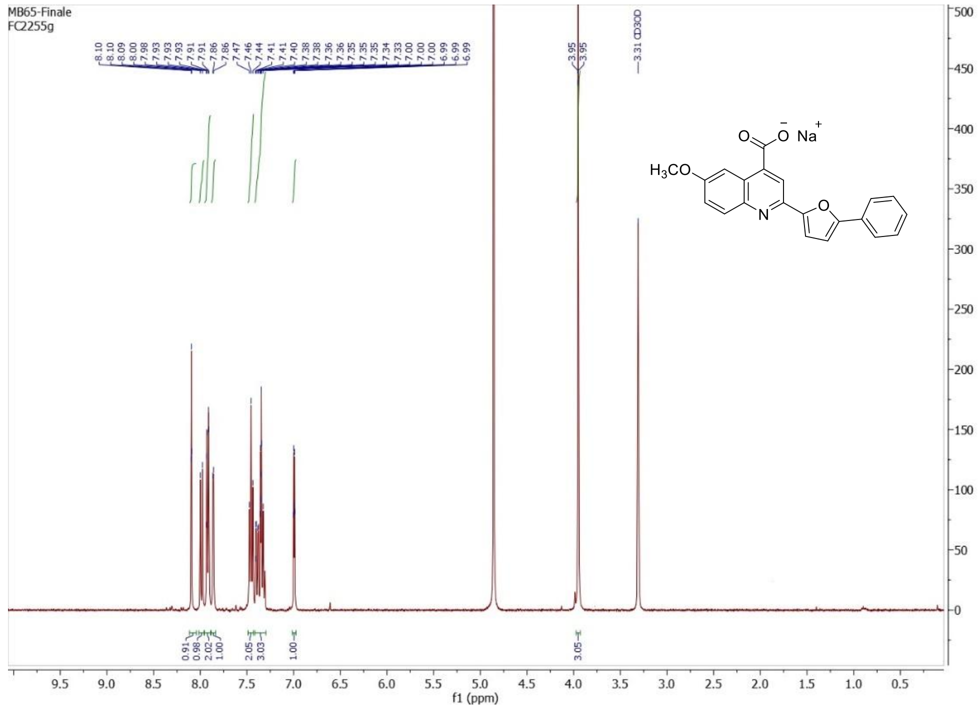

^{13}C-NMR spectrum (101 MHz, CD_3OD) of **19**

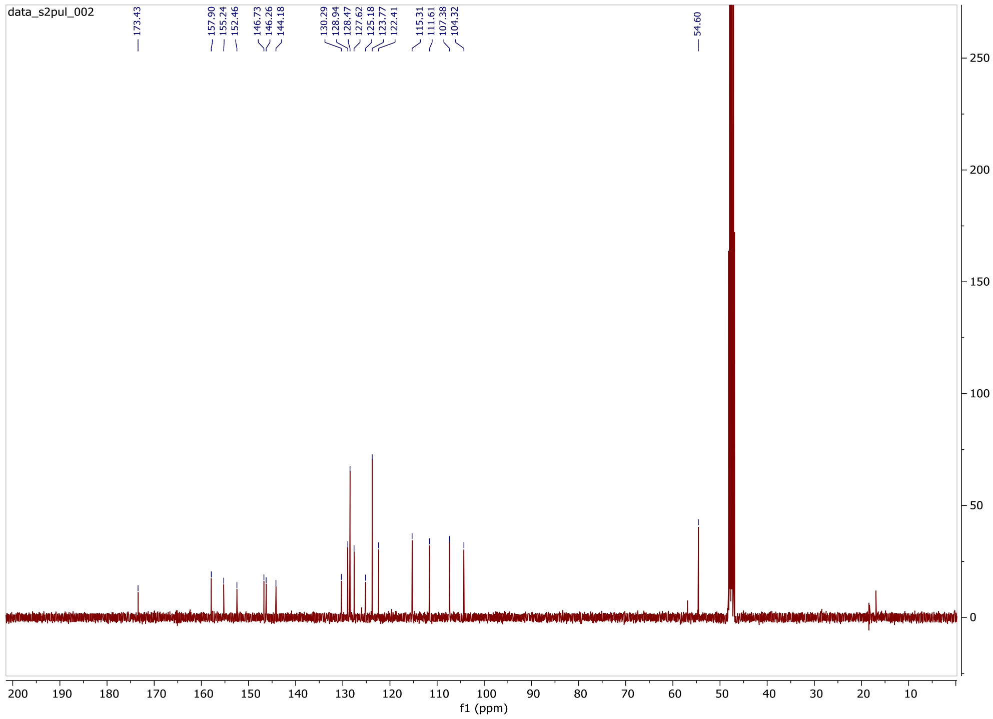

UHPLC-MS analysis of **19**

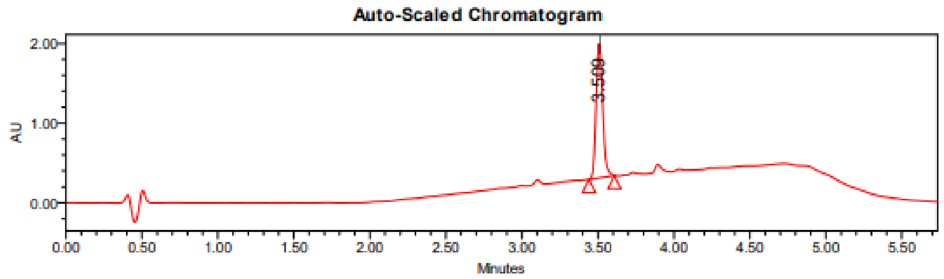

Processed Channel: W2489 ChA 215nm

	Processed Channel	Retention Time (min)	Area	% Area	Height
1	W2489 ChA 215nm	3.509	5017882	100.00	1673673

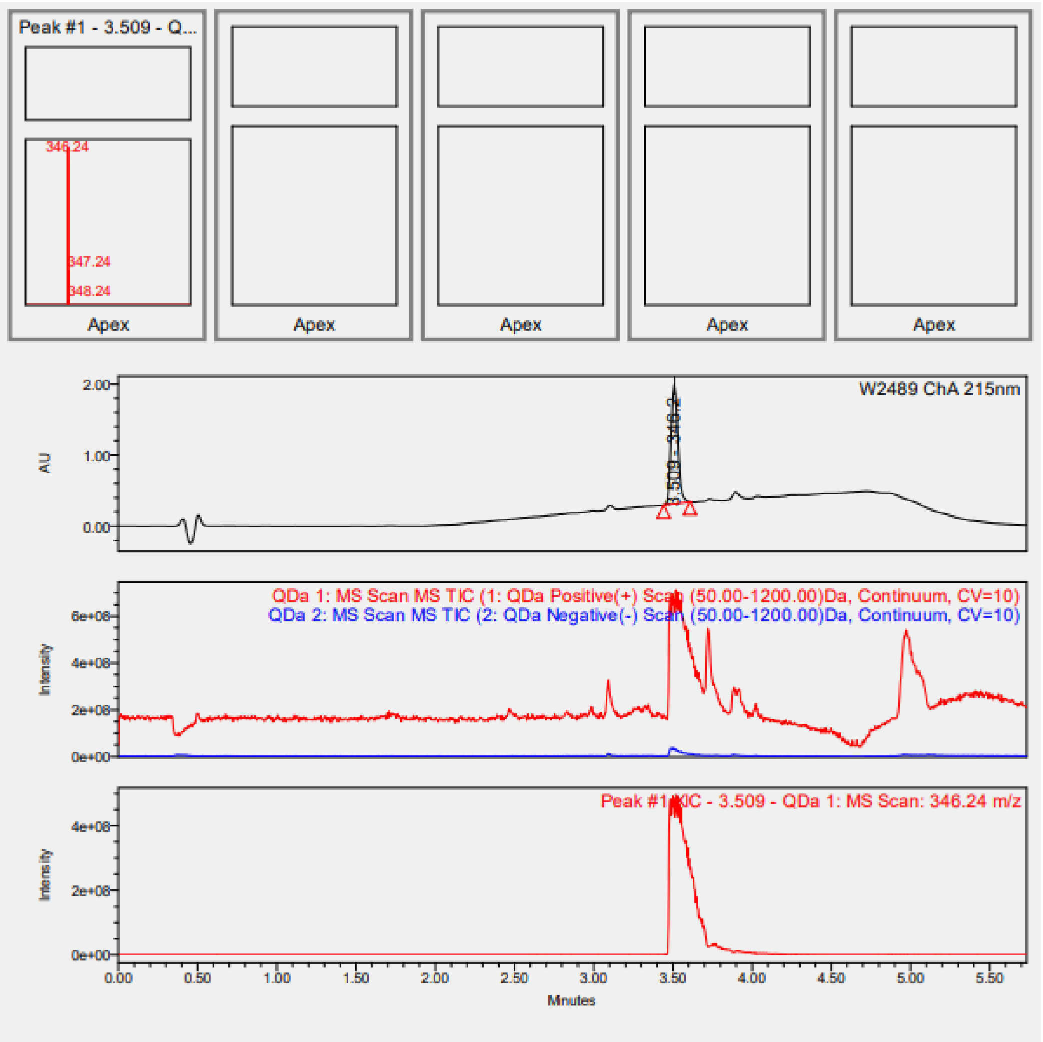

^{1}H-NMR spectrum (401 MHz, DMSO-d_6) of **20**

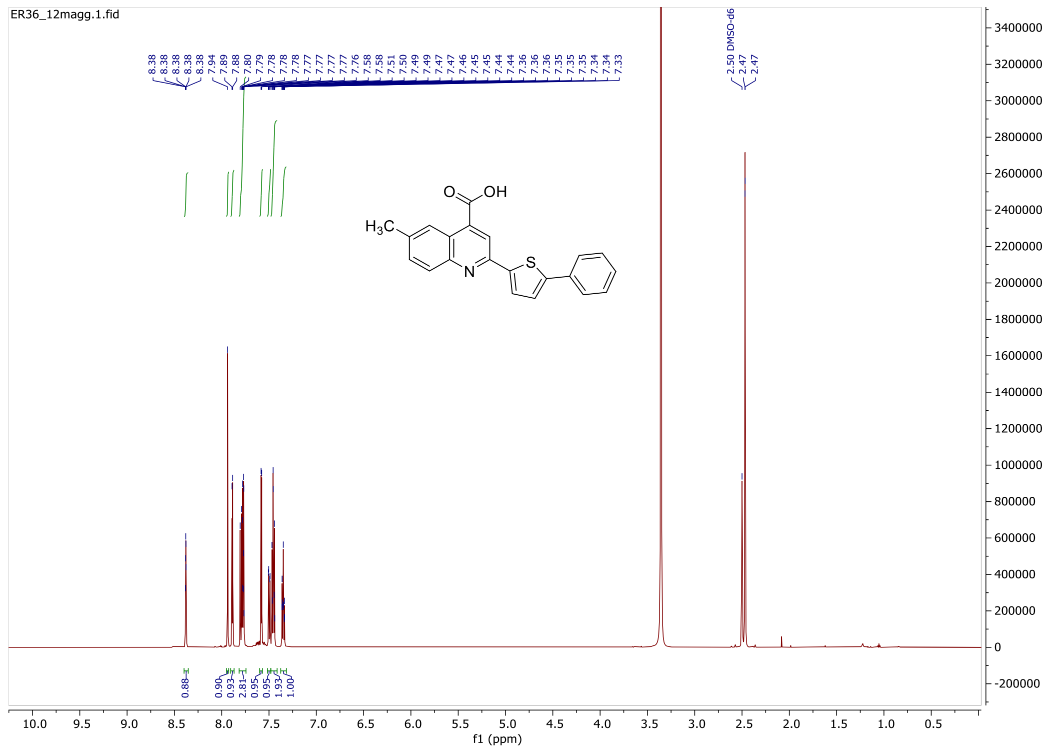

^{13}C-NMR spectrum (101 MHz, DMSO-d_6) of **20**

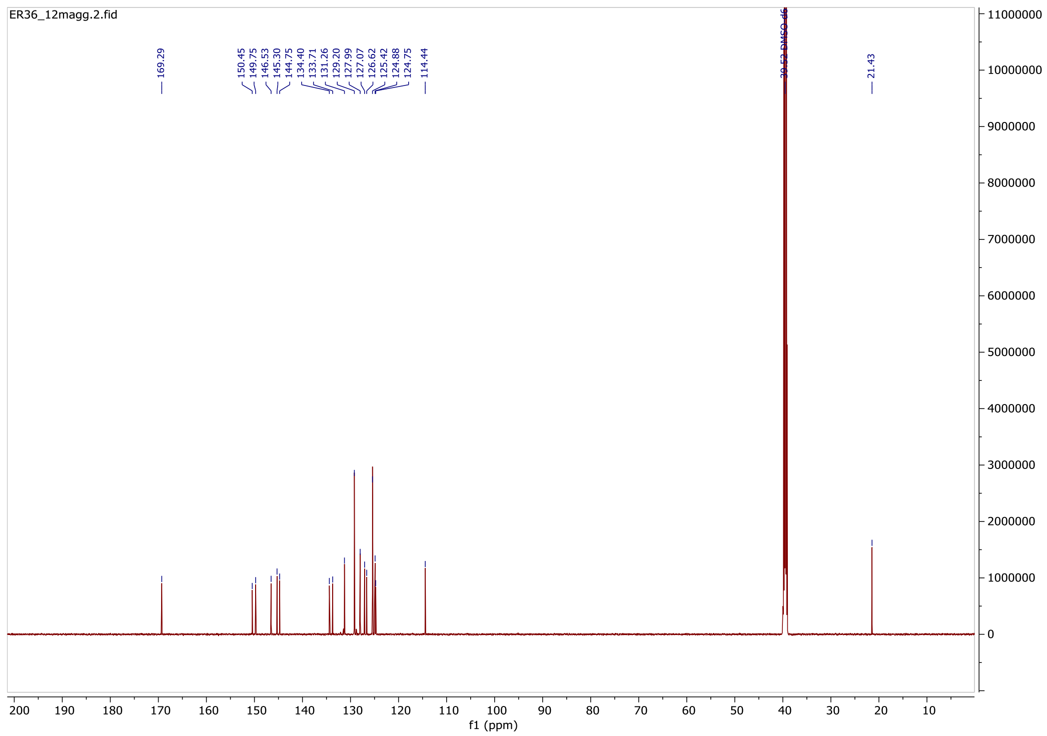

UHPLC-MS analysis of **20**

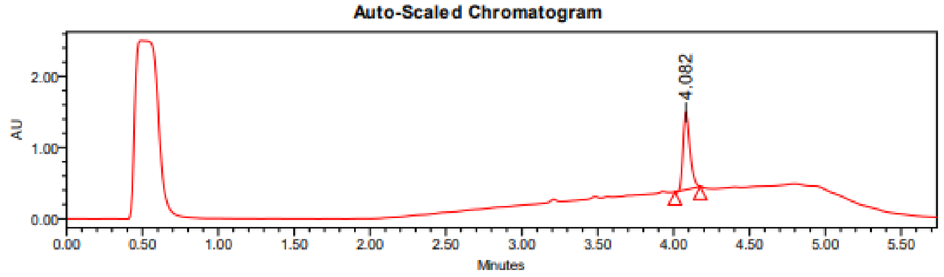

Processed Channel: W2489 ChA 215nm

	Processed Channel	Retention Time (min)	Area	% Area	Height
1	W2489 ChA 215nm	4.082	3470709	100.00	1085692

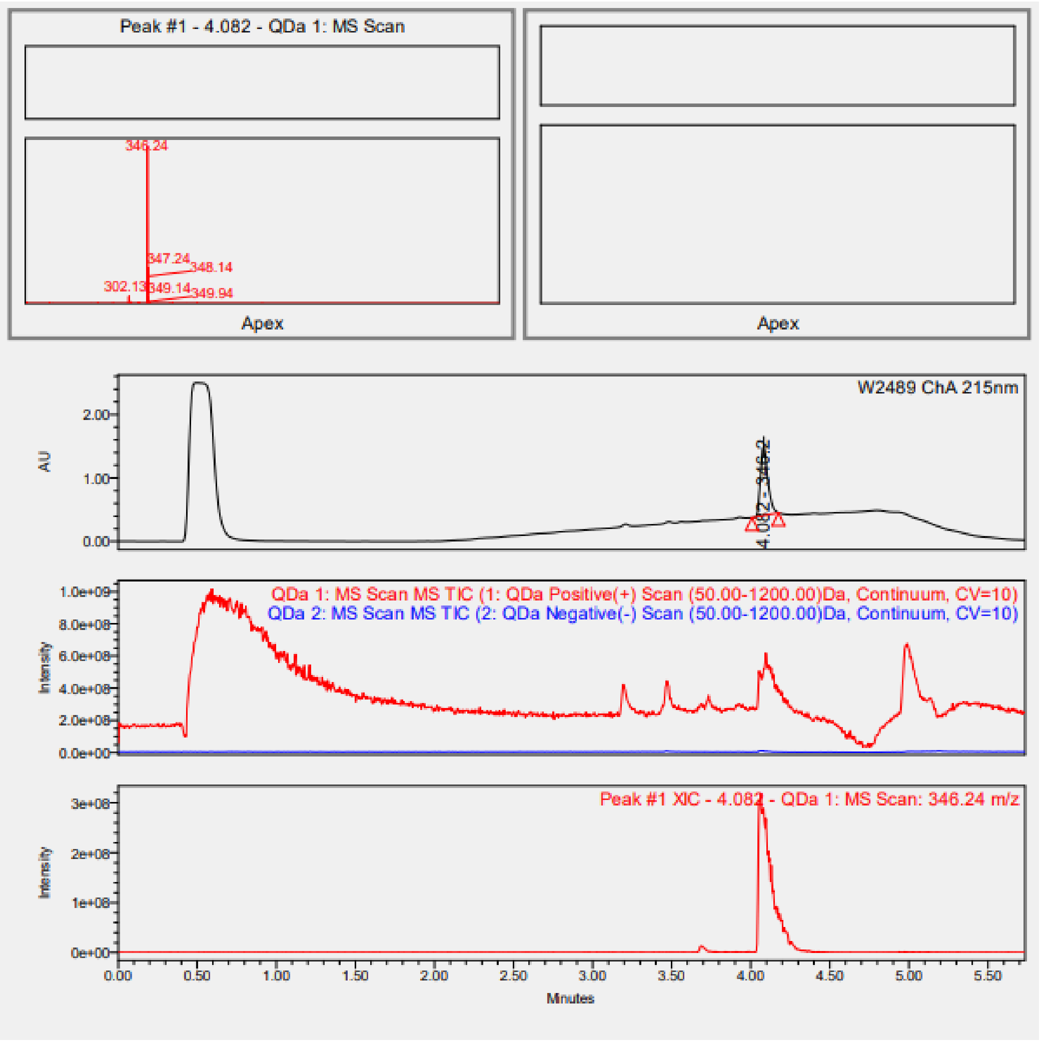

References

(1) Bagnolini, G.; Milano, D.; Manerba, M.; Schipani, F.; Ortega, J. A.; Gioia, D.; Falchi, F.; Balboni, A.; Farabegoli, F.; De Franco, F.; et al. Synthetic Lethality in Pancreatic Cancer: Discovery of a New RAD51-BRCA2 Small Molecule Disruptor That Inhibits Homologous Recombination and Synergizes with Olaparib. *J Med Chem* **2020**, *63* (5), 2588-2619. DOI: 10.1021/acs.jmedchem.9b01526 From NLM Medline.

(2) Yu. I Horak, M. D. O., R.V. Kutsyk, R.Z. Lytvyn, L.M. Kurovets. 2-(5-Aryl-2-furyl)quinolin-4-carboxylic acids and their antimicrobial activity. *Ukraina Bioorganica Acta* **2008**, *6*, 49-54.

(3) Rajendra, E.; Venkitaraman, A. R. Two modules in the BRC repeats of BRCA2 mediate structural and functional interactions with the RAD51 recombinase. *Nucleic Acids Res* **2010**, *38* (1), 82-96. DOI: 10.1093/nar/gkp873 From NLM Medline.

(4) Scott, D. E.; Francis-Newton, N. J.; Marsh, M. E.; Coyne, A. G.; Fischer, G.; Moschetti, T.; Bayly, A. R.; Sharpe, T. D.; Haas, K. T.; Barber, L.; et al. A small-molecule inhibitor of the BRCA2-RAD51 interaction modulates RAD51 assembly and potentiates DNA damage-induced cell death. *Cell Chem Biol* **2021**, *28* (6), 835-847 e835. DOI: 10.1016/j.chembiol.2021.02.006 From NLM Medline.

(5) Myers, S. H.; Poppi, L.; Rinaldi, F.; Veronesi, M.; Ciamarone, A.; Previtali, V.; Bagnolini, G.; Schipani, F.; Ortega Martinez, J. A.; Girotto, S.; et al. An (19)F NMR fragment-based approach for the discovery and development of BRCA2-RAD51 inhibitors to pursuit synthetic lethality in combination with PARP inhibition in pancreatic cancer. *Eur J Med Chem* **2024**, *265*, 116114. DOI: 10.1016/j.ejmech.2023.116114 From NLM Medline.

(6) Dalvit, C.; Caronni, D.; Mongelli, N.; Veronesi, M.; Vulpetti, A. NMR-based quality control approach for the identification of false positives and false negatives in high throughput screening. *Curr Drug Discov Technol* **2006**, *3* (2), 115-124. DOI: 10.2174/157016306778108875 From NLM Medline.

(7) Dalvit, C.; Fasolini, M.; Flocco, M.; Knapp, S.; Pevarello, P.; Veronesi, M. NMR-Based screening with competition water-ligand observed via gradient spectroscopy experiments: detection of high-affinity ligands. *J Med Chem* **2002**, *45* (12), 2610-2614. DOI: 10.1021/jm011122k From NLM Medline.

(8) Roberti, M.; Schipani, F.; Bagnolini, G.; Milano, D.; Giacomini, E.; Falchi, F.; Balboni, A.; Manerba, M.; Farabegoli, F.; De Franco, F.; et al. Rad51/BRCA2 disruptors inhibit homologous recombination and synergize with olaparib in pancreatic cancer cells. *Eur J Med Chem* **2019**, *165*, 80-92. DOI: 10.1016/j.ejmech.2019.01.008 From NLM Medline.

(9) Dos Santos Ferreira, A. C.; Fernandes, R. A.; Kwee, J. K.; Klumb, C. E. Histone deacetylase inhibitor potentiates chemotherapy-induced apoptosis through Bim upregulation in Burkitt's lymphoma cells. *J Cancer Res Clin Oncol* **2012**, *138* (2), 317-325. DOI: 10.1007/s00432-011-1093-y From NLM.

(10) Falchi, F.; Giacomini, E.; Masini, T.; Boutard, N.; Di Ianni, L.; Manerba, M.; Farabegoli, F.; Rossini, L.; Robertson, J.; Minucci, S.; et al. Synthetic Lethality Triggered by Combining Olaparib with BRCA2-Rad51 Disruptors. *ACS Chem Biol* **2017**, *12* (10), 2491-2497. DOI: 10.1021/acschembio.7b00707 From NLM Medline.

(11) Rossi, V.; Hochkoeppler, A.; Govoni, M.; Di Stefano, G. Lactate-Induced HBEGF Shedding and EGFR Activation: Paving the Way to a New Anticancer Therapeutic Opportunity. *Cells* **2024**, *13* (18). DOI: 10.3390/cells13181533 From NLM Medline.

(12) Masi, M.; Biundo, F.; Fiou, A.; Racchi, M.; Pascale, A.; Buoso, E. The Labyrinthine Landscape of APP Processing: State of the Art and Possible Novel Soluble APP-Related Molecular Players in Traumatic Brain Injury and Neurodegeneration. *Int J Mol Sci* **2023**, *24* (7). DOI: 10.3390/ijms24076639 From NLM.

(13) Buoso, E.; Masi, M.; Limosani, R. V.; Fagiani, F.; Oliviero, C.; Colombo, G.; Cari, L.; Gentili, M.; Lusenti, E.; Rosati, L.; et al. Disruption of Epithelial Barrier Integrity via Altered GILZ/c-Rel/RACK1 Signaling in Inflammatory Bowel Disease. *Journal of Crohn's and Colitis* **2024**, *19* (1). DOI: 10.1093/ecco-jcc/jjae191 (acccessed 1/31/2025).

(14) Bresciani, G.; Hofland, L. J.; Dogan, F.; Giamas, G.; Gagliano, T.; Zatelli, M. C. Evaluation of Spheroid 3D Culture Methods to Study a Pancreatic Neuroendocrine Neoplasm Cell Line. *Front Endocrinol (Lausanne)* **2019**, *10*, 682. DOI: 10.3389/fendo.2019.00682 From NLM.

(15) Baumann, K. W.; Baust, J. M.; Snyder, K. K.; Baust, J. G.; Van Buskirk, R. G. Characterization of Pancreatic Cancer Cell Thermal Response to Heat Ablation or Cryoablation. *Technol Cancer Res Treat* **2017**, *16* (4), 393-405. DOI: 10.1177/1533034616655658 From NLM.
(16) Nunes, J. P. S.; Dias, A. A. M. ImageJ macros for the user-friendly analysis of soft-agar and wound-healing assays. *Biotechniques* **2017**, *62* (4), 175-179. DOI: 10.2144/000114535 From NLM.
(17) Chen, W.; Wong, C.; Vosburgh, E.; Levine, A. J.; Foran, D. J.; Xu, E. Y. High-throughput image analysis of tumor spheroids: a user-friendly software application to measure the size of spheroids automatically and accurately. *J Vis Exp* **2014**, (89). DOI: 10.3791/51639 From NLM.
(18) Masi, M.; Poppi, L.; Previtali, V.; Nelson, S. R.; Wynne, K.; Varignani, G.; Falchi, F.; Veronesi, M.; Albanesi, E.; Tedesco, D.; et al. Investigating synthetic lethality and PARP inhibitor resistance in pancreatic cancer through enantiomer differential activity. *Cell Death Discov* **2025**, *11* (1), 106. DOI: 10.1038/s41420-025-02382-3 From NLM PubMed-not-MEDLINE.
